# Patient, Caregiver, and Clinician Participation in Prioritization of Research Questions in Pediatric Hospital Medicine

**DOI:** 10.1001/jamanetworkopen.2022.9085

**Published:** 2022-04-26

**Authors:** Peter J. Gill, Ann Bayliss, Aubrey Sozer, Francine Buchanan, Karen Breen-Reid, Kim De Castris-Garcia, Mairead Green, Michelle Quinlan, Noel Wong, Shelley Frappier, Katherine Cowan, Carol Chan, Dana Arafeh, Mohammed Rashid Anwar, Colin Macarthur, Patricia C. Parkin, Eyal Cohen, Sanjay Mahant

**Affiliations:** 1 Division of Paediatric Medicine, The Hospital for Sick Children, Toronto, Ontario, Canada; 2 Department of Pediatrics, University of Toronto, Toronto, Ontario, Canada; 3 Child Health Evaluative Sciences, SickKids Research Institute, Toronto, Ontario, Canada; 4 Institute of Health Policy, Management and Evaluation, University of Toronto, Toronto, Ontario, Canada; 5 Trillium Health Partners, Department of Pediatrics, University of Toronto, Mississauga, Ontario, Canada; 6 Research Family Advisory Committee, SickKids Research Institute, The Hospital for Sick Children, Toronto, Ontario, Canada; 7 Lawrence Bloomberg Faculty of Nursing, University of Toronto, Toronto, Ontario, Canada; 8 Parent Advocate, The Hospital for Sick Children, Toronto, Ontario, Canada; 9 Department of Pediatrics, University of Ottawa, Children’s Hospital of Eastern Ontario (CHEO), Ottawa, Ontario, Canada; 10 Learning Institute, The Hospital for Sick Children, Toronto, Ontario, Canada; 11 James Lind Alliance, Southampton, United Kingdom; 12 Department of Family and Community Medicine, University of Toronto, Toronto, Ontario, Canada

## Abstract

**Importance:**

The research agenda in pediatric hospital medicine has seldom considered the perspectives of young people, parents and caregivers, and health care professionals. Their perspectives may be useful in identifying questions on topics for research.

**Objective:**

To prioritize unanswered research questions in pediatric hospital medicine from the perspectives of young people, parents/caregivers, and health care professionals.

**Design, Setting, and Participants:**

Between August 4, 2020, and August 19, 2021, two online surveys and a virtual workshop were conducted, using modified Delphi technique and nominal group technique. Young people, parents/caregivers, and health care professionals with experiences in pediatric hospital medicine in Canada were included.

**Interventions:**

The established James Lind Alliance Priority Setting Partnership method was used. In phase 1, a survey collected unanswered questions regarding pediatric hospital medicine via 3 open-ended questions. Survey responses were used to develop summary questions that went through an evidence-checking process. Unanswered questions were brought to a phase 2 interim prioritization survey. The top 10 unanswered research questions in pediatric hospital medicine were established at the final priority setting workshop.

**Main Outcomes and Measures:**

Survey responses, top 10 research questions.

**Results:**

The phase 1 survey was completed by 188 participants (148 of 167 [89%] females; 17 of 167 [10%] males; mean [SD] age, 39.5 [12.4] years) and generated 495 unanswered research questions and comments, of which 58 were deemed out of scope. The remaining 437 responses were grouped into themes (eg, communication, shared decision-making, health service delivery, and health service management) and then refined to 75 unanswered research questions. Of these 75, only 4 questions had sufficient evidence. To make the number of questions in phase 2 manageable, 21 questions submitted by only 1 respondent were eliminated. Fifty unanswered research questions were included in the phase 2 survey, which was completed by 201 participants (165 of 186 [89%] females; 19 of 186 [10%] males; mean [SD] age, 40.0 [11.0] years). A short list of 16 questions—the top 10 questions from patient partners (youths, parents/caregivers) and clinicians—was presented at the final priority setting workshop and the top 10 questions were prioritized. The top 10 questions focused on the care of special inpatient populations (eg, children with medical complexity), communication, shared decision-making, support strategies in the hospital, mental health supports, shortening length of stay, and supporting Indigenous patients, parents/caregivers, and families.

**Conclusions and Relevance:**

This patient-oriented pediatric hospital medicine priority setting partnership identified the most important unanswered research questions focused on the care of children in the hospital. These questions provide a possible roadmap for research on areas deemed important to young people, parents/caregivers, and clinicians.

## Key Points

**Question:**

What are the highest-priority unanswered research questions in pediatric hospital medicine from the perspective of young people, parents/caregivers, and health care professionals?

**Findings:**

This study, which included 2 surveys and a final consensus meeting using nominal group technique, gathered the perspectives of youths, parents/caregivers, and clinicians. The top 10 questions identified focused on the care of special inpatient populations (eg, children with medical complexity), communication, shared decision-making, support strategies, mental health supports, reducing time in the hospital, and supporting Indigenous families.

**Meaning:**

The findings of this study suggest that the most important research questions in pediatric hospital medicine focus on processes and models of care, communication, and hospitalization outcomes.

## Teaser

This survey study develops questions from the perspectives of youths, parents/caregivers, and health care professionals on areas to address in pediatric hospital medicine research.

## Introduction

Admission to the hospital can be a challenging time for children and families because of the physical effects of serious illness and the psychological effects of illness and hospitalization.^1^ There are also economic strains on families because of work absences and other indirect costs of hospitalization (eg, transportation).^2^ In Canada and the US, most children cared for in hospitals have medical conditions managed in general pediatric inpatient units (GPIUs).^3,4^ Children admitted to GPIUs may have been previously healthy and are hospitalized for an acute, common illness (eg, gastroenteritis) or have chronic conditions (eg, asthma) and are hospitalized for an acute, common illness or exacerbation of the chronic disease. The specialty dedicated to caring for these hospitalized children in the GPIU is pediatric hospital medicine. Over the past decade, seminal research has contributed to improvements in the care of hospitalized children in select areas, such as bronchiolitis, and handoff bundles (ie, medical handover).^5,6,7,8^ Yet, compared with other hospital-based clinical areas, such as oncology^9^ and critical care,^10,11^ there remains a relative lack of high-quality research in pediatric hospital medicine, particularly robust randomized clinical trials.^12,13^

Although there have been health systems level analyses identifying high-priority conditions for research in pediatric hospital medicine based on volume, costs, and variation in care in the US and Canada,^4,14,15,16^ little is known about which topics should be prioritized for research from the perspective of patients, families, and clinicians. Prioritization processes can help minimize avoidable research waste^17^ by mitigating the mismatch between research that is conducted and research that is relevant to patients and clinicians.^18^ National research bodies, such as the Patient-Centered Outcomes Research Institute in the US^19^ and the Strategy for Patient-Oriented Research in Canada,^20^ suggest engaging patients, caregivers, and stakeholders in defining research priorities. James Lind Alliance (JLA) Priority Setting Partnerships (PSPs) bring together patients, caregivers, and clinicians to identify important unanswered questions in clinical areas. Priority Setting Partnerships have been used for a range of conditions and care settings,^21^ such as emergency care^22^ and intensive care.^23^ The JLA method facilitates high levels of patient engagement, with patients involved in all steps of the research process.^21,24^ Engaging patients and families as coresearchers has been reported to have several benefits, including increased response rates,^25,26,27,28,29^ credibility of results,^26,27^ credibility of dissemination,^29^ and quality of results and rigor of research.^30,31^

To establish meaningful relationships and maximize research results, patients and caregivers should be engaged early in research design, particularly in the research question conceptualization phase.^30^ To our knowledge, no previous studies have identified the most important research questions in pediatric hospital medicine from the perspective of patients, caregivers, and clinicians. Patient and family involvement in pediatric research prioritization studies have focused on preventive care,^32^ children with neurodevelopmental problems,^33^ emergency care,^34^ and patient safety in the hospital.^35^ Therefore, the primary aim of our project was to conduct a research priority setting study using a JLA PSP to identify the most important unanswered clinical management questions in pediatric hospital medicine that are meaningful to patients, caregivers, and clinicians.

## Methods

### Study Design

We conducted a JLA PSP^36^ using a modified Delphi technique along with a nominal group technique from March 1, 2020, to September 28, 2021.^37^ Our protocol is available in eMethods 1 in Supplement 1. The study followed the Reporting Guideline for Health Research Priority Setting with Stakeholders (REPRISE). The project was reviewed by the Hospital for Sick Children’s Research Ethics Board and deemed to not require approval because its activity fell in the domain of preresearch/information gathering. The entire study, including each survey and the workshop, was explicitly about identifying research questions to be used for future research projects.

### Scope

The research priority setting study focused on clinical management questions relevant to the care of children (age 0-18 years) hospitalized in a GPIU in Canada. We used the American Academy of Pediatrics definition of pediatric hospital medicine: “patients with acute and/or serious complications of common problems, multiple comorbidities and/or injuries, complex chronic diseases, acute mental health problems, special health care needs, technology-dependent conditions, and those needing palliative care.”^38^ Clinical management was broadly defined as the diagnosis and treatment of conditions, including processes of care (eg, how to promote better sleep in the hospital) as well as specific conditions (eg, interventions for asthma). The PSP focused on conditions in which clinical management was typically led by general pediatricians and did not distinguish between GPIUs located in a children’s hospital or community hospital.

Given the breadth of childhood mental health conditions, we excluded questions related to the in-hospital management of known mental health problems, such as depression, that would require specialist input (eg, psychiatry). We also excluded questions related to chronic or preexisting mental health problems of parents and caregivers. Questions that focused on specialized settings of care, such as the pediatric or neonatal intensive care unit, medical subspecialty units (eg, oncology), and psychiatric and surgical inpatient units, were also excluded. In addition, we excluded questions related to the cause of the disease or prognosis and those related to health care organization and/or health care delivery (eg, staffing levels).

### Steering Group

A steering group was formed, cochaired by a JLA advisor (K.C.) and the project lead (P.J.G.), which included 1 young person (M.B.), 3 parents/caregivers, 3 pediatricians, and 2 nurses. The steering group met monthly to organize the PSP activities, including defining the scope, recruiting participants, developing and disseminating surveys, overseeing analysis and interpretation of results, determining questions to be discussed at the final priority setting workshop, and conducting activities for knowledge translation.

### Phase 1: Initial Survey

An initial survey (available in English and French) was developed by the steering group and pilot tested before wide distribution via email and social media to study partners and networks, including professional societies, research organizations, knowledge users, patient-oriented research organizations, and patient family advisory groups. The survey was active from August 4 to November 9, 2020. The anonymous survey was open to patients, parents/caregivers, and clinicians with experiences in the GPIU.

Multiple recruitment approaches were used to ensure the diversity of respondents, including targeting vulnerable groups, specifically Indigenous communities and high social risk groups. We developed a video explaining the priority setting process,^39^ ensured that the graphics in communication materials captured a diverse range of patients and parents/caregivers (eg, graphics included individuals using a wheelchair and those with different ethnicities), used colors that met accessibility standards, and provided a paper version of the survey to facilitate participation of those who did not have access to online technology.

Anonymous study data were collected and managed using Research Electronic Data Capture (REDCap),^40^ hosted at The Hospital for Sick Children. The survey (eMethods 3 in Supplement 1) asked participants the following question: What concerns, comments or questions do you have about the care of children in the hospital on the general pediatric ward that you would like answered by research? There were also optional demographic questions on occupation, age, gender, ethnicity, place of residence (province/territory), and setting of residence (urban/rural) to track respondent types and target dissemination efforts toward underrepresented groups. Descriptive statistics were used to summarize participant demographic characteristics and survey responses.

### Forming Summary Questions

Phase 1 survey data were analyzed in Microsoft Excel, version 15 (Microsoft Corp). Following removal of out-of-scope questions, the raw questions were reviewed iteratively by the PSP coordinator (D.A.) with regular discussions with co-chairs of the steering group (P.J.G. and K.C.). Each response was coded into broad categories, developed iteratively after reviewing the responses several times. Similar or duplicate questions were combined where appropriate. To assist, steering group members were paired into dyads comprising clinicians and youths and/or parents/caregivers; each dyad was responsible for reviewing up to 5 categories of responses and tasked with forming summary questions. The steering group provided oversight to ensure appropriate interpretation of the raw data through open discussion in group meetings to ensure that questions were understandable to all audiences. Examples of raw data for all final prioritized questions can be found on the JLA website.^41^

### Evidence Checking

Highly focused and targeted literature searches were conducted to determine whether the summary questions had already been answered. Given the broad scope, questions were considered unanswered if there was no systematic review, if a recent systematic review indicated insufficient evidence, or if there was insufficient evidence outlined in position statements from the Canadian Paediatric Society, American Academy of Pediatrics, or National Institute for Health and Care Excellence. The searches were conducted by an information specialist (M.R.A.) who worked with an experienced librarian (Q.M.); a detailed description of the search strategy is available online (eMethods 2 in Supplement 1).^42^ To ensure retrieval of the most recent literature, searches were restricted to 2010 onward, were limited to material published in English, and were conducted from December 13, 2020, to April 4, 2021. Questions deemed to be answered by earlier research were categorized as answered. The remaining unanswered questions were brought forward to phase 2. Initial categorization of the evidence was completed by one of us (M.R.A.) and was then reviewed (P.J.G.); final decisions were made by consensus, with input of the steering group as required.

### Phase 2: Interim Prioritization Survey

A phase 2 interim prioritization survey (available in English and French) was administered online from May 3 to July 12, 2021. This survey consisted of 2 parts: part 1 asked participants to select randomly ordered unanswered questions they thought were important and part 2 presented the previously selected questions and asked participants to select up to 10 questions perceived to be the most important. The survey was distributed as in phase 1 and included the same optional demographic data.

Phase 2 data were analyzed in Microsoft Excel. Each time a question was selected, it was assigned 1 point; if a participant chose more than 10 questions, each point was divided by the total number of questions selected. To ensure equal weighting between the perspectives of participants with lived experience (patients and parents/caregivers) and that of clinicians, points for each participant category were tallied separately, generating a total for lived experience and for clinician perspectives for each question. Within each group, the total points for each question were ranked from high to low and given a new score according to their ranking. These scores were summed to calculate a total combined score for each question and sorted by combined ranked order. The steering group reviewed the rankings and reached consensus on which questions to bring forward to phase 3.

### Phase 3: Final Priority Setting Workshop

Two half-day (August 18-19, 2021) virtual (Zoom Technologies Inc) workshop sessions were held to generate consensus on the top 10 unanswered questions in pediatric hospital care. In-person meetings were not possible because of the COVID-19 pandemic; therefore, the JLA adapted its workshop delivery method to be conducted online, and learnings from other JLA PSPs were applied.^43^ The steering group, with guidance from the JLA, recruited 24 youths, parents/caregivers, and clinicians from across Canada for the workshop. Workshop documentation and guidance was sent to participants 3 weeks before the workshop. Childcare reimbursement was offered, and compensation was provided to patients and/or caregivers according to published guidelines.^20^

The workshop was chaired by one of us (K.C.) and used a modified nominal group technique,^44,45^ which is a well-established structured, multistep facilitated group meeting technique. Three additional JLA facilitators managed the small-group discussions to ensure balanced contributions from youths, parents/caregivers, and clinicians. We evaluated the extent of patient engagement in the final workshop via a survey.

## Results

### Phase 1: Initial Survey

The phase 1 survey was completed by 188 participants (Table 1), with 54 responses (29%) from those with lived experience and 134 responses (71%) from clinicians. Most respondents were female (148 of 167 [89%] vs 17 of 167 [10%] male]; mean [SD] age, 39.5 [12.4] years). Self-reported ethnicities were African (n = 3), Asian (eg, Middle East and South, East, and Southeast Asian; n = 28), Caribbean (n = 2), European (n = 5), Indigenous (First Nations, Metis, Inuit; n = 2); Latin, Central, or South American (n = 3); White (North American or European; n = 136); and prefer not to say (n = 5) or other (n = 2). There was representation from most regions of Canada, primarily Ontario (86 of 167 [52%]).

**Table 1.** Characteristics of Respondents to First-Phase and Second-Phase Surveys

Characteristic	No. (%)	
	Survey 1 (n = 188)	Survey 2 (n = 201)
Which of the following best describes you?		
Child/young person	9	10
Parent/caregiver	41	33
Friend/family member	4	1
Nurse	41	42
Physician	65	59
Dietician	3	8
Child life specialist	3	13
Respiratory therapist	5	3
Pharmacist	1	0
Social worker	2	1
Physiotherapist	2	3
Occupational therapist	3	7
Other	9	21
What is your current age in years? mean (SD)^a^	39.5 (12.4)	40.0 (11.0)
How do you identify?^a^		
Male	17 (11)	19 (10)
Female	148 (89)	165 (89)
Other	1 (0.5)	0
Prefer not to say	1 (0.5)	2 (1)
What is your ethnic/cultural background?^b^		
African	3	4
Asian (eg, Middle East and South, East, and Southeast Asian)	28	21
Caribbean	2	5
European	5	6
Indigenous (First Nations, Metis, Inuit)	2	4
Latin, Central, or South American	3	1
White (North American or European)	136	165
Prefer not to say	5	5
Other	2	2
Language spoken at home^a^		
English	160	173
French	26	35
Other	9	13
What province or territory do you live in?^a^		
British Columbia	12	11
Prairies^c^	39	28
Ontario	86	92
Quebec	22	34
Maritimes^d^	6	15
Newfoundland and Labrador	1	2
Territories^e^	1	1
What best describes where you live?^a^		
Urban	147	168
Rural	22	27
How many times have you/your child been hospitalized?^a^		
1-2	34	46
3-5	21	29
>5	26	22
NA	78	89
What was age of you/your child when admitted to hospital?^a^		
<3 mo	18	19
3 mo-2 y	16	16
2-5 y	11	13
5-9 y	9	8
10-14 y	9	16
15-18 y	10	11
Prefer not to say	1	4
NA	80	93
For health and/or social care practitioners, what setting do you practice in?^a^		
Community hospital in urban/suburban area	30	24
Community hospital in rural area	8	7
Academic children’s hospital	89	122
Prefer not to say	0	2
Other	5	2
Not applicable	18	13

Abbreviation: NA, not applicable.

^a^ Denotes that question was optional and responses do not sum to total number of respondents.

^b^ Based on self-report. Other refers to Black of Afro-Caribbean descent (n = 1), Canadian (n = 1), Lebanese (n = 1), mixed background (n = 1). European included as separate category from White (North American or European) for those who identified as European but not White.

^c^ Prairies includes Alberta, Manitoba, and Saskatchewan.

^d^ Maritimes includes Nova Scotia, New Brunswick, and Prince Edward Island.

^e^ Territories includes Yukon and Northwest Territories.

In total, the 188 participants provided 495 questions/statements. Of the 495 responses, 58 were deemed out of scope, for example, because they focused on surgery, intensive care, or resource allocation (Figure). The remaining 437 questions/statements were sorted into 22 categories (eTable 1 in Supplement 1); the 4 categories with the largest number of responses were treatment (n = 73), communication (n = 56), management (n = 37), and family experience and support (n = 37). With steering group oversight, 75 summary questions were drafted.

**Figure.**
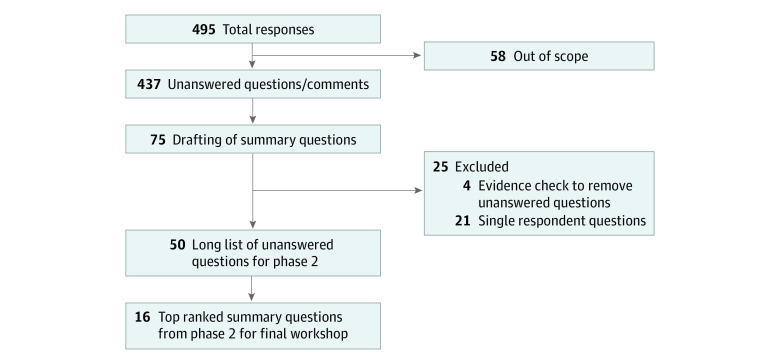
Flowchart of Responses by Survey Participants

### Evidence Checking

Of the 75 questions, 4 were deemed to have sufficient evidence to mark as answered (Table 2). The steering group reviewed the remaining 71 questions and identified 21 that were supported by a single respondent (eTable 2 in Supplement 1); these 21 questions were discussed by the steering group to ensure that they did not represent issues of concern to groups who were underrepresented in the survey and then were pragmatically removed to make the phase 2 survey more manageable for respondents. Of the original 75 questions, 50 went forward to phase 2; all 71 questions are listed in eTable 3 in Supplement 1, with correspondence evidence identified in eTable 4 in Supplement 1.

**Table 2.** Questions With Sufficient Evidence

Question No.	Question
2	What comfort care and pain management techniques, including medical, holistic, and nonmedical, are effective in hospitalized children on the general pediatric inpatient unit?
20	What are the most effective and safe intravenous fluids to use on children and youths hospitalized in the general pediatric inpatient unit?
48	What are evidence-based protocols for the safe management and discharge of hospitalized infants with jaundice on the general pediatric inpatient unit?
59	Does including interpreters for all communications between health care professionals and families/patients improve care for hospitalized children on the general pediatric inpatient unit?

### Phase 2: Interim Prioritization Survey

In total, 201 participants completed the phase 2 survey (Table 1), with 44 responses (22%) from those with lived experience and 157 responses (78%) from clinicians. Most respondents were women (165 of 186 [89%] vs 19 of 186 [10%] men; mean [SD] age, 40.0 [11.0] years). Self-reported ethnicities were African (n = 4), Asian (eg, Middle East and South, East, and Southeast Asian; n = 21), Caribbean (n = 5), European (n = 6), Indigenous (First Nations, Metis, Inuit; n = 4); Latin, Central, or South American (n = 1); White (North American or European; n = 165); and prefer not to say (n = 5) or other (n = 2). Most participants were from Ontario (92 of 183 [50%]). European was included as a separate category from White (North American or European) for those who identified as European but not White.

Survey responses were sorted by overall rank, lived experience responses, and clinician responses (Table 3). The steering group reviewed the overall ranking and unanimously decided to include all questions that fell into either group’s (lived experience and clinician) top 10 items for the workshop. As such, a total of 16 questions were brought forward for discussion in phase 3 (Figure).

**Table 3.** Summary of Combined Ranking of the Top 20 Questions From the Phase 2 Survey by 201 Participants

Combined rank^a^	Summary question	Lived experience rank	Health care professional rank
1	What is the most effective way to conduct medical rounds, including how to involve caregivers and patients in the decision-making while on the GPIU?	= 1	3
2	What mental health supports can be provided to parents, families, and children and youths while hospitalized on the GPIU?	3	2
3	What methods of communication are most effective between patients, caregivers, and health care providers on a GPIU?	4	9
= 4	How can we ensure that health care delivery in the hospital meets the needs of children and youths with developmental disabilities on the GPIU?	7	7
= 4	What are effective support strategies for parents, families, and children and youths hospitalized on the GPIU? (eg, support groups, private rooms/sleeping arrangements, breastfeeding support, physical activity, making the ward more adolescent-friendly, screen time)	= 1	13
6	What are best practices and support strategies for Indigenous parents, families, and children and youths on the GPIU?	= 15	1
7	What best practices and/or care models exist for inpatient care for children and youths with medical complexity on the GPIU?	= 11	6
8	What are effective methods (eg, education) to prepare families for discharge from the GPIU?	6	12
= 9	What are the most effective communication methods (eg, handover, rounds) between health care providers on a GPIU?	8	11
= 9	What are effective alternatives to shorten length of stay for hospitalized children and youths on the GPIU? (eg, hospitalization at home, early discharge with close and regular follow-up)	14	5
11	When is it appropriate to involve allied health care professionals (eg, OT, PT, child life specialists) in the care of children/youths hospitalized on the GPIU?	= 17	4
12	What is the impact of the patient’s room/environment on health outcomes on the GPIU? (eg, noise, lights, private/shared room, window/no window)	5	19
= 13	What is the most effective way to obtain and maintain IV access in children and youths hospitalized on the GPIU?	= 17	8
= 13	What are effective strategies to mitigate the impacts of prolonged inpatient hospitalizations on GPIUs? (eg, addressing unmet needs, prolonged separation from family)	= 15	10
15	Are ongoing mental health assessments for patients admitted to a GPIU beneficial?	= 9	22
16	Do communication systems or tools that connect health care providers on the GPIU with community providers improve care of patients?	= 21	14
17	What best practices and/or care models exist around discharge for children and youths with medical complexity on the GPIU?	= 11	26
18	What are ways to structure multiple clinical assessments and consultations to minimize discomfort and reduce unnecessary tests or treatments on children hospitalized on the GPIU?	= 21	18
19	What are effective ways to incorporate shared decision-making with parents and children/youths hospitalized on the GPIU? (effectiveness defined as length of stay, caregiver confidence)	= 9	31
20	What are ways to support breastfeeding mothers when their breastfed infant is hospitalized on the GPIU?	= 29	15

Abbreviations: GPIU, general pediatric inpatient unit; IV, intravenous; OT, occupational therapy; PT, physical therapy.

^a^ Use of the = symbol denotes equal rank.

### Phase 3: Final Priority Setting Workshop

The workshop involved 24 participants: 12 clinicians (4 pediatricians, 5 nurses, 2 child life specialists, and 1 occupational therapist), 10 parents/caregivers, and 2 youth patients from across Canada. In addition, 4 experienced JLA PSP facilitators (including K.C.) and 3 observers were present. The workshop participants engaged in thought-provoking discussions over 2 half-day sessions before reaching consensus on the top 10 unanswered research questions (Table 4).

**Table 4.** List of the Top 10 Unanswered Research Questions Focused on Pediatric Hospital Medicine

Rank	Question
1	What best practices and/or care models exist for inpatient care for children and youths with medical complexity on the GPIU?
2	What methods of communication are most effective between patients, caregivers, and health care providers on a GPIU?
3	What are best practices and support strategies for Indigenous parents, families, and children and youths on the GPIU?
4	How can we ensure that health care delivery in the hospital meets the needs of children and youths with developmental disabilities on the GPIU?
5	What are effective support strategies for parents, families, and children and youths hospitalized on the GPIU? (eg, support groups, private rooms/sleeping arrangements, breastfeeding support, physical activity, making the ward more adolescent-friendly, screen time)
6	What mental health supports can be provided to parents, families, and children and youths while hospitalized on the GPIU?
7	What are effective ways to incorporate shared decision-making with parents and children/youths hospitalized on the GPIU? (effectiveness defined as length of stay, caregiver confidence)
8	What are effective strategies to mitigate the impacts of prolonged inpatient hospitalizations on GPIUs? (eg, addressing unmet needs, prolonged separation from family)
9	What are effective alternatives to shorten length of stay for hospitalized children and youths on the GPIU? (eg, hospitalization at home, early discharge with close and regular follow-up)
10	What are the most effective communication methods (eg, handover, rounds) between health care providers on a GPIU?

Abbreviation: GPIU, general pediatric inpatient unit.

In total, 23 workshop attendees (96%) completed the feedback survey. Overall, 100% of participants strongly agreed or agreed that small groups were a good method of discussion, 100% agreed (84% strongly agreed) that all participants were encouraged to join in discussions equally, and 94% believed they had an opportunity to learn a great deal from others.

## Discussion

Using the approach recommended by national research bodies,^19,20^ this PSP brought together youths, patients, parents/caregivers, and clinicians to identify the top 10 research priorities for pediatric hospital medicine. To our knowledge, this project is the first to examine major knowledge gaps related to the in-hospital care of children by integrating the perspectives of those with lived experience and clinicians. By involving patients and parents/caregivers in the process, we provided a voice to important stakeholders, including those who may only have a brief stay in the hospital or who do not fall within a specific disease entity for research. The top 10 research priorities lay the foundation for researchers and research networks to focus on improving outcomes in pediatric hospital medicine. The long list of 71 questions (eTable 3 in Supplement 1) identifies several important clinical areas in need of further research.^41^

Using the Krumholz framework of outcomes research,^46^ most of the top questions focus on the patient perspective, rather than comparative effectiveness or health systems improvement. Although there were specific research questions identified in the survey (eg, effective use of heated high-flow nasal canula; eTable 3 in Supplement 1), the top research questions were quite broad compared with other PSPs, particularly those addressing a specific condition or clinical area,^33,47,48,49^ which reflects the comprehensive clinical area covered. Our scope encompassed clinical management, which comprised diagnosis and treatment, including processes of care (eg, insertion of an intravenous cannula) and specific conditions (eg, bronchiolitis). Other PSPs that have focused on broad areas, such as occupational therapy,^43^ intensive care,^23^ or emergency medicine,^22^ had similarly broad questions yet were able to generate focused research questions that were funded.^50,51^ For example, a top unanswered question in intensive care was on supportive families after discharge, leading to a multicenter longitudinal study exploring the outcomes of children and families in the first year after discharge from intensive care.^51^ The themes identified in the top 10 questions are a salient reminder that families are less concerned about minor differences regarding which test is ordered or which treatment is given compared with whether shared decision-making was conducted^52^ or how supported they felt during hospitalization.^53^ These sentiments were not only highlighted by those with lived experience but were also ranked highly by clinicians given the significant overlap in phase 2 survey rankings (Table 3). Although many questions are broad, this implies that subsequent research can potentially have a large influence on clinical care. For example, research to increase communication between families and clinicians or on integrated shared decision-making can be broadly applicable to multiple conditions in the hospital.

The top research priority focused on best practices and care models for hospitalized children with medical complexity. These children often have functional limitations, substantial family-identified needs, and a reliance on technologies for activities of daily living.^54^ Diskin et al^55^ conducted a 3-stage modified Delphi study, including clinicians and family caregivers, to identify research priorities for children with neurological impairment and medical complexity. Most top-ranked questions focused on the management of specific clinical issues, such as feeding and irritability, and none focused on in-hospital care. There are knowledge gaps on the optimal models of care (eg, primary inpatient care, consultative or episode-based care model)^56^ and on how best to support parents/caregivers^57^ addressing, for example, whether in-hospital care should be led by specialized complex care teams, with or without trainees; the role of the outpatient clinicians in the inpatient setting; whether a different care model should be implemented at children’s vs community hospitals; and how family perspectives on patient care models should be evaluated.

Specific populations were explicitly addressed in 2 of the other top 10 questions. The first focused on how to meet the needs of children and youths with developmental disabilities on the GPIU. Although there is an overlap with children with medical complexity, final workshop participants felt strongly that children and youths with developmental disabilities face unique issues in the hospital. One earlier Canadian JLA PSP identified research gaps for children with neurodevelopmental disorders, but primarily focused on outpatient care.^58^ There were concerns about the lack of hospital-friendly initiatives or resources for these children (eg, strategies to support a child with autism spectrum disorder admitted with an asthma exacerbation while using inhalers). The second focused on Indigenous parents, families, and children and youths—a community whose children have disproportionately higher hospitalization rates—compared with children of other ethnic groups.^59^ Despite this increased burden, few studies have examined the care and outcomes of Indigenous children on the GPIU or evaluated how to ensure meaningful participation of Indigenous communities in health research. Given the failure of past research endeavors, including causing harm,^60^ initiatives to improve care in this population will require building reciprocal, trusting relationships with Indigenous stakeholders.

The outcome associated with hospitalization was another important theme. Prioritizing alternatives to shorten the length of hospital stay reflects that 65% of all hospital encounters in children are at community and general hospitals.^3,4^ Although there has been extensive research in adult care about alternatives to inpatient care, such as hospital-at-home interventions,^61^ there are limited examples in pediatrics, such as home phototherapy for neonatal jaundice^62^ or home oxygen therapy for bronchiolitis.^63^ There was also recognition of the need to mitigate the outcomes of prolonged inpatient hospitalization, such as excessive screen time for children and separation from families.^64^ The mental health outcome of hospitalization was also a prominent discussion point in the final workshop because the COVID-19 pandemic has exacerbated mental health concerns among children and youths as well as parents/caregivers. A priority was family support, such as counseling, recognizing the influence of parental mental health on children.

### Limitations and Strengths

Strengths of our project include applying the JLA PSP approach, which is a well-used, rigorous, validated methodological approach to identify research priorities. We also had sufficient representation from a range of health care professionals from across Canada and had 100% attendance at the final workshop. The number of respondents to both surveys was consistent with several other JLA PSPs.^47,49^ The top priorities for pediatric hospital medicine are also likely relevant for hospitalized children in other care settings, such as in oncology or surgical inpatient units. Furthermore, the priority setting method can inform other research groups who want to identify priorities for similarly broad care settings, such as adult internal medicine.

The project has limitations. First, the scope of the PSP was broad, which introduced challenges in communicating relevance to families in the survey. The project also required that the evidence-checking progress was restricted to systematic reviews and guidelines that may have missed important, high-quality research related to the summary questions. Second, there was limited representation from allied health care workers from community hospitals in both surveys, and from individuals who work in rural and remote regions. Third, although demographic information on survey participants was optional, most of the participants identified as White (North American or European), with few participants identifying as Indigenous. Despite this limitation, the third ranked question was focused on Indigenous parents, families, and children and youths. There were also few respondents who identified as Latin, Caribbean, and African. Fourth, the top 10 questions reflect broad areas of clinical care and may prove challenging to address with research. However, early work with our research network^65^ has already identified multiple specific questions to be pursued.

## Conclusions

Generation of the most important unanswered clinical management questions in pediatric hospital medicine, which reflect the priorities of patients, parents/caregivers, and clinicians, provides a strategic, patient-oriented research agenda on the treatment of children hospitalized in GPIUs. The key partnerships developed through this work may provide a foundation for patient-oriented research projects to improve the evidence base. Research networks are uniquely positioned to operationalize these research priorities, ranging from studies focused on service delivery to large, multicenter trials conducted in academic and community hospitals.

## Notes

Supplement 1. **eTable 1.** Summary of Categories Used to Classify Responses (n = 437) **eTable 2.** List of Unanswered Questions Submitted by a Single Respondent (n = 21) **eTable 3.** Long List of All Unanswered Research Questions in Pediatric Hospital Medicine (n = 71) **eTable 4.** Data Management Spreadsheet Used by Pediatric Hospital Care Priority Setting Partnership **eMethods 1.** Pediatric Hospital Care Priority Setting Partnership Protocol May 25, 2020, Version 2 **eMethods 2.** Pediatric Hospital Care Priority Setting Partnership Question Verification Form **eMethods 3.** Phase 1 Initial Survey Used in Pediatric Hospital Care Priority Setting Partnership

Supplement 2. **Nonauthor Collaborators.** The Canadian Pediatric Inpatient Research Network (PIRN)
