# Supplementary material for: Patient, Caregiver, and Clinician Participation in Prioritization of Research Questions in Pediatric Hospital Medicine
Source: JAMA Netw Open. 2022 Apr 26;5(4):e229085. doi: 10.1001/jamanetworkopen.2022.9085 (PMC9044112; doi:10.1001/jamanetworkopen.2022.9085)
Supplement: Supplement 1. — eTable 1. Summary of Categories Used to Classify Responses (n = 437) eTable 2. List of Unanswered Questions Submitted by a Single Respondent (n = 21) eTable 3. Long List of All Unanswered Research Questions in Pediatric Hospital Medicine (n = 71) eTable 4. Data Management Spreadsheet Used by Pediatric Hospital Care Priority Setting Partnership eMethods 1. Pediatric Hospital Care Priority Setting Partnership Protocol May 25, 2020, Version 2 eMethods 2. Pediatric Hospital Care Priority Setting Partnership Question Verification Form eMethods 3. Phase 1 Initial Survey Used in Pediatric Hospital Care Priority Setting Partnership [file jamanetwopen-e229085-s001.pdf]

Supplemental Online Content

Gill PJ, Bayliss A, Sozer A, et al; Canadian Pediatric Inpatient Research Network (PIRN). Patient, caregiver, and clinician participation in prioritization of research questions in pediatric hospital medicine. *JAMA Netw Open.* 2022;5(4):e229085. doi:10.1001/jamanetworkopen.2022.9085

eTable 1. Summary of Categories Used to Classify Responses (n = 437)

eTable 2. List of Unanswered Questions Submitted by a Single Respondent (n = 21)

eTable 3. Long List of All Unanswered Research Questions in Pediatric Hospital Medicine (n = 71)

eTable 4. Data Management Spreadsheet Used by Pediatric Hospital Care Priority Setting Partnership

eMethods 1. Pediatric Hospital Care Priority Setting Partnership Protocol May 25, 2020, Version 2

eMethods 2. Pediatric Hospital Care Priority Setting Partnership Question Verification Form

eMethods 3. Phase 1 Initial Survey Used in Pediatric Hospital Care Priority Setting Partnership

This supplemental material has been provided by the authors to give readers additional information about their work.

© 2022 Gill PJ et al. *JAMA Network Open*.

eTable 1. Summary of categories used to classify responses (n=437).

**Categories**	**Number of Responses**
Communication	56
Care coordination	6
Diagnosis	10
Discharge planning	18
Education	10
Efficiency	7
Environment	5
Family experience and support	37
Health services, delivery and management	34
Holistic care	23
Hospital policy	16
Management	37
Mental health	14
Monitoring	2
Patient centered outcomes	1
Practice variation & evidence	7
Quality	18
Shared decision making	13
Staff roles	10
Tests	35
Treatment	73
Virtual care	5

© 2022 Gill PJ et al. *JAMA Network Open*.

eTable 2. List of unanswered questions submitted by a single respondent (n=21)

1.	What is the most effective way to wean respiratory support (e.g. oxygen, heated high flow nasal cannula) in hospitalized children and youth with respiratory illnesses (e.g. bronchiolitis, asthma) on a general pediatric inpatient unit?
2.	Are additional doses of corticosteroids effective in hospitalized children and youth with croup on a general pediatric inpatient unit?
3.	What is the utility of routine blood cultures for children and youth hospitalized with common condition on a general pediatric inpatient unit?
4.	What are effective interventions for children and youth hospitalized with cyclic vomiting on the general pediatric inpatient unit?
5.	Does collecting and assessing patient-reported and patient-important outcomes for all hospitalized children/youth on a general pediatric inpatient unit improve outcomes?
6.	What are best practices when using intravenous immune globulin (IVIG) in hospitalized children/youth on the general pediatric inpatient unit?
7.	What are effective uses of albumin infusions in hospitalized children/youth hospitalized on the general pediatric inpatient unit?
8.	When should physicians complete nasopharyngeal swabs for respiratory viral infections in children/youth hospitalized on a general pediatric inpatient unit?
9.	What are the adverse effects of sedation on children/youth hospitalized on a general pediatric ward?
10.	What are the most effective diagnostic tests to diagnose meningitis in children/youth hospitalized on a general pediatric inpatient unit?
11.	What are effective treatments for children/youth with acute kidney disease hospitalized on a general pediatric inpatient unit?
12.	What is the most effective treatment for children/youth with aspiration pneumonia hospitalized on a general pediatric inpatient unit?
13.	What are the adverse effects of nebulized epinephrine on patients, caregivers and healthcare professionals on the general pediatric inpatient unit?
14.	What is the effectiveness of oseltamivir for hospitalized children/youth with influenza on a general pediatric ward?
15.	What is the effectiveness of procalcitonin in diagnosing a serious bacterial infections in children/youth hospitalized in a general pediatric inpatient unit?
16.	Can a national network improve care for children/youth on the general pediatric inpatient unit?
17.	What are non-antimicrobial therapies for hospitalized children/youth with chronic otitis media on the general pediatric inpatient unit?
18.	What tools or techniques can increase the success rate of lumbar punctures in children and youth hospitalized on a general pediatric inpatient unit?
19.	What are risk factors for serious underlying illnesses in children who are hospitalized with a brief resolved unexplained event (BRUE) on the general pediatric inpatient unit?
20.	How should children and youth with non-cancer related febrile neutropenia be managed on the general pediatric inpatient unit?
21.	What are effective non-sedate (eg. feed and sleep) diagnostic tests (eg. CT scans) for children and youth admitted on a general pediatric inpatient unit?

© 2022 Gill PJ et al. *JAMA Network Open*.

eTable 3. Long list of all unanswered research questions in pediatric hospital medicine (n=71)

1. Are ongoing mental health assessments for patients admitted to a general pediatric inpatient unit beneficial?
2. What are effective methods (e.g. education) to prepare families for discharge from the general pediatric inpatient unit?
3. What educational content provided at discharge can support families and reduce hospital re-admissions to the general pediatric inpatient unit?
4. What best practices and/or care models exist for inpatient care for children and youth with medical complexity on the general pediatric inpatient unit?
5. What best practices and/or care models exist around discharge for children and youth with medical complexity on the general pediatric inpatient unit?
6. What methods of communication are most effective between patients, caregivers and health care providers on a general pediatric inpatient unit?
7. Do communication systems or tools that connect healthcare providers on the general pediatric inpatient unit with community providers improve care of patients?
8. What are the most effective communication methods (e.g. handover, rounds, etc.) between healthcare providers on a general pediatric inpatient unit?
9. What communication or information sharing methods are effective at explaining tests to patients and caregivers on the general pediatric inpatient unit?
10. What is the most effective route (i.e. IV vs PO) and duration of antibiotic therapy for children and youth with bacteremia (of known origin e.g. UTI vs unknown source) on a general pediatric inpatient unit?
11. What is the most effective route (i.e. IV vs PO) and duration of antibiotic therapy for urinary tract infections (UTIs) with and without a negative blood culture?
12. What is the most effective way to use oxygen monitoring (e.g. intermittent, continuous) for hospitalized children with common respiratory illnesses (e.g. bronchiolitis) on the general pediatric inpatient unit?
13. What is the most effective way to wean respiratory support (e.g. oxygen, heated high flow nasal cannula) in hospitalized children and youth with respiratory illnesses (e.g. bronchiolitis, asthma) on a general pediatric inpatient unit?
14. What is the most effective way (i.e. population, illness, initiation, weaning) to utilize heated high flow nasal cannula (HHFNC) in hospitalized children and youth on the general pediatric inpatient unit?
15. What is the most appropriate way to provide fluid therapy (e.g. IV, nasogastric tube) to children and youth hospitalized with bronchiolitis on a general pediatric inpatient unit?
16. What is the most effective route (i.e. IV vs PO), optimal indications, duration of antibiotic therapy for children and youth hospitalized with cellulitis on a general pediatric inpatient unit?
17. What are effective treatments for children and youth hospitalized with an acute asthma exacerbation on a general pediatric inpatient unit?
18. Are additional doses of corticosteroids effective in hospitalized children and youth with croup on a general pediatric inpatient unit?
19. What are the most effective and safe intravenous fluids to use on children and youth hospitalized in the general pediatric inpatient unit?
20. What is the utility of routine blood cultures children and youth in hospitalized with common condition on a general pediatric inpatient unit?
21. What is the most effective way to obtain and maintain IV access in children and youth hospitalized on the general pediatric inpatient unit?

© 2022 Gill PJ et al. *JAMA Network Open*.

22. What are potential uses of point of care ultrasound in the general pediatric inpatient unit?
23. What are effective strategies to mitigate the impacts of prolonged inpatient hospitalizations on general pediatric inpatient units? e.g. addressing unmet needs, prolonged separation from family
24. What warning systems/monitoring tools are effective at improving outcomes for hospitalized children and youth on a general pediatric inpatient unit?
25. What are effective interventions for children and youth hospitalized with cyclic vomiting on the general pediatric inpatient unit?
26. What are the most effective interventions in infants, children and youth with gastroesophageal reflux disease (GERD) on the general peadiatric inpatient unit?
27. What is the most effective way to standardize care and reduce unnecessary variation in care delivered to hospitalized children and youth on the general pediatric inpatient unit?
28. What is the most effective treatment strategy for hospitalized children and youth with neurologic impairment who suffer from chronic respiratory illness on the general pediatric inpatient unit?
29. How can we ensure that healthcare delivery in hospital meets the needs of children and youth with developmental disabilities on the general pediatric inpatient unit?
30. What are effective support strategies for parents, families and children/youth hospitalized on the general pediatric inpatient unit? E.g. support groups, private rooms/sleeping arrangements, breastfeeding support, physical activity, making the ward more adolescent-friendly, screen time, Indigenous communities and spiritual care
31. Can the nutritional needs of children/youth and their families be addressed as an essential part of the therapeutic care plan on a general pediatric inpatient unit and what are effective methods to support children and families' nutritional needs?
32. When it is appropriate to involve allied health care professionals (e.g. OT, PT, child life specialists) in the care of hospitalized children/youth on the general pediatric inpatient unit?
33. What are best practices and support strategies for Indigenous parents, families and children/youth on the general pediatric inpatient unit?
34. What are ways to support breastfeeding mothers when their breastfed infant is hospitalized on the general pediatric inpatient unit?
35. What are effective ways to incorporate patients and families unique values, goals, and socio-demographic factors to optimize care on the general pediatric inpatient unit?
36. What is the impact of the patient's room/environment on health outcomes on the general pediatric inpatient unit? E.g. noise, lights, private/shared room, window/no window
37. What are ways to structure multiple clinical assessments and consultations to minimize discomfort and reduce unnecessary tests or treatments on children hospitalized on the general pediatric inpatient unit ?
38. What are effective ways to incorporate shared decision-making with parents and children/youth hospitalized on the general pediatric inpatient unit? "Effectiveness" defined as length of stay, caregiver confidence
39. What is the most effective way to conduct medical rounds, including how to involve caregivers and patients in the decision making while on the general pediatric inpatient unit?
40. How does the integration of virtual care on the general pediatric inpatient unit help to limit communication barriers affecting the outcome in patient and family experience?
41. Does collecting and assessing patient-reported and patient-important outcomes for all hospitalized children/youth on a general pediatric inpatient unit improve outcomes?
42. What are best practices when using intravenous immune globulin (IVIG) in hospitalized children/youth on the general pediatric inpatient unit?

© 2022 Gill PJ et al. *JAMA Network Open*.

43. Does the implementation of quality indicators lead to improved safety and reduced medical errors in the general pediatric inpatient unit? (e.g. communication with medical team, medication errors, patient safety reviews, etc.)
44. What are effective alternatives to shorten length of stay for hospitalized children/youth? Eg, hospitalization at home, early discharge with close and regular follow-up), in the general pediatric in-patient unit
45. What is the most effective way to perform vital sign measurements while ensuring patient and family comfort on the general pediatric inpatient unit?
46. What mental health supports can be provided to parents, families and children/youth while hospitalized on the general pediatric inpatient unit?
47. What are effective uses of albumin infusions in hospitalized children/youth hospitalized on the general pediatric inpatient unit?
48. What are best practices during respiratory viral season to reduce spread of nosocomial infections amongst and prevent outbreaks hospitalized children/youth on the general pediatric inpatient unit?
49. When should physicians complete nasopharyngeal swabs for respiratory viral infections in children/youth hospitalized on a general pediatric inpatient unit?
50. Can a national EMR system improve care on a general pediatric ward?
51. Does ensuring continuity of care of healthcare professionals improve the care and outcomes of hospitalized children/youth on the general pediatric inpatient unit?
52. What are the adverse effects of sedation on children/youth hospitalized on a general pediatric ward?
53. What are the most effective diagnostic tests to diagnose meningitis in children/youth hospitalized on a general pediatric inpatient unit?
54. What are effective treatments for children/youth with acute kidney disease hospitalized on a general pediatric inpatient unit?
55. What is the most effective treatment for children/youth with complicated pneumonia hospitalized on a general pediatric inpatient unit?
56. What is the most effective treatment for children/youth with aspiration pneumonia hospitalized on a general pediatric inpatient unit?
57. What are adverse effects of nebulized epinephrine on patients, caregivers and healthcare professionals on the general pediatric inpatient unit?
58. How are ethical principles and guidelines influencing the care of hospitalized children on the general pediatric inpatient unit?
59. What is the effectiveness of oseltamivir for hospitalized children/youth with influenza on a general pediatric ward?
60. What are effective tools to increase adherence with recommended therapies for hospitalized children/youth on a general pediatric ward?
61. What is the effectiveness of procalcitonin in diagnosing a serious bacterial infection in children/youth hospitalized in a general pediatric inpatient unit?
62. What are medication-related information management system or tool improves outcomes of children/youth hospitalized in a general pediatric inpatient unit?
63. Can a national network improve care for children/youth on the general pediatric inpatient unit?
64. What are the most effective methods for oral and nasal suctioning (i.e aggressive vs minimalist, cough assist vs deep suctioning) of hospitalized children/youth on a general pediatric inpatient unit?
65. What are non-antimicrobial therapies for hospitalized children/youth with chronic otitis media on the general pediatric inpatient unit?

© 2022 Gill PJ et al. *JAMA Network Open*.

66. What are effective treatments for children/youth and adolescents hospitalized with eating disorders on the general pediatric inpatient unit?
67. What are risk factors for serious underlying illnesses in children who are hospitalized with a brief resolved unexplained event (BRUE) on the general pediatric inpatient unit?
68. What are effective interventions for hospitalized children and youth with sickle cell disease on the general pediatric inpatient unit? E.g. Hyperhydration, oxygen, antibiotics, pain medication
69. How should children and youth with non-cancer related febrile neutropenia be managed on the general pediatric inpatient unit?
70. What are evidence-based guidelines on the management of infants under 1 month / 3 month with fever without a source?
71. What are effective non-sedate (eg. feed and sleep) diagnostic tests (eg. CT scans) for children and youth admitted on a general pediatric inpatient unit?

© 2022 Gill PJ et al. *JAMA Network Open*.

eTable 4. Data management spreadsheet used by Pediatric Hospital Care Priority Setting Partnership

Project ID	Uncertainty (PICO formatted)	Original uncertainty	Evidence	Source of Uncertainty
1	Are ongoing mental health assessments for patients admitted to a general paediatric inpatient unit beneficial?	What is the awareness of mental health in patients and any training to support the language/interactions they have with children to support their whole care mind, body and spirit while hospitalized<br>How does an admission to a general paediatric ward affect patients' overall mood and mental health?<br>What is the impact of isolation precautions on clinical outcomes and child/family coping?<br>Involving a mental health assessment with every pediatric patient admitted to hospital (and knowing how to do this) What kind of psychological impact do hospital procedures have on kids post admission?<br>What kind of psychological impact do hospital procedures have on kids post admission?	CPS statement: A wide range of tools are available for screening. Nothing specific to general pediatric inpatient unit;<br>https://www.cps.ca/en/mental-health-screening-tools;<br>AAP guideline: List of tools available; not specific to inpatient unit<br>https://downloads.aap.org/AAP/PDF/Mental_Health_Tools_for_Pediatrics.pdf<br>NICE guideline: No guidelines<br><br>Newton, A. S., Soleimani, A., Kirkland, S. W., & Gokiert, R. J. (2017). A systematic review of instruments to identify mental health and substance use problems among children in the emergency department. Academic emergency medicine, 24(5), 552-568.<br>Carter, T., Walker, G. M., Aubeeluck, A., & Manning, J. C. (2019). Assessment tools of immediate risk of self-harm and suicide in children and young people: A scoping review. Journal of child health care, 23(2), 178-199.<br>Kirkland, S. W., Soleimani, A., Gokiert, R., & Newton, A. S. (2017). MP33: A systematic review of the psychometric properties and diagnostic performance of instruments to identify mental health and substance use problems among children in the emergency department. Canadian Journal of Emergency Medicine, 19(S1), S76-S76.<br>Manning, J. C., Walker, G. M., Carter, T., Aubeeluck, A., Witchell, M., & Coad, J. (2018). Children and Young People-Mental Health Safety Assessment Tool (CYP-MH SAT) study: Protocol for the development and psychometric evaluation of an assessment tool to identify immediate risk of self-harm and suicide in children and young people (10–19 years) in acute paediatric hospital settings. BMJ open, 8(4), e020964.<br>Song, K., Lorber, S., Mian, I., Leslie, K., Crosbie, J., & Munns, C. (2018, October). Brief Screening Tools for Pediatric Patients With Depressive or Anxiety Disorders: A Scoping Review. In 65th Annual Meeting. AACAP.<br><br>Verdict: There are lots of validated assessment tools as identified in CPS but their application to the GPIU is unclear. The systematic review by Newton et al, also identifies some tools but those are specific to ED. **This question has been partially addressed in the evidence base.**	2 x parent/caregiver<br>3 x nurse<br>1 x youth
2	What comfort care and pain management techniques, including medical, holistic and non-medical, are effective in hospitalized children on the general paediatric inpatient unit?	What comfort care and pain management tactics are being used in pediatric care?<br>What is the best way to care for patients with chronic pain? Or patients with chronic illness that have pain related to this.<br>Is Tylenol and Advil alternated every 3 hours more effective for pain then giving together?<br>All types of procedural pain control and anxiety relief in the paediatric patient and underutilization<br>Can we do everything to make these stressful painful procedures less so for kids?<br>Effect of non-medical pain management.<br>morphine seems to negatively affect teenage boys, aren't there other options for pain management?<br>How to minimize the pain of hospital tests<br>Pain relief in hospital pediatrics.<br>Distractions that can be used to help minimize pain or anxiety,<br>How we can improve patient care through the usage of language	CPS statement: Guidelines are available;<br>Trottier ED, Doré-Bergeron MJ, Chauvin-Kimoff L, Baerg K, Ali S. Managing pain and distress in children undergoing brief diagnostic and therapeutic procedures. Paediatrics & child health. 2019 Dec 9;24(8):509-21.<br>AAP guideline: Guidelines are available:<br>PAC A. Pain Management in Infants, Children, Adolescents and Individuals with Special Health Care Needs. https://www.aapd.org/research/oral-health-policies--recommendations/pain-management-in-infants-children-adolescents-and-individuals-with-special-health-care-needs/[Specific to dental cases];<br>Lee GY, Yamada J, Shorkey A, Stevens B. Pediatric clinical practice guidelines for acute procedural pain: a systematic review. Pediatrics. 2014 Mar 1;133(3):500-15.<br>NICE guideline: No guidelines<br><br>Andersen, R. D., Olsson, E., & Eriksson, M. (2020). The Evidence Supporting the Association Between the Use of Pain Scales and Outcomes in Hospitalized Children: a Systematic Review. International Journal of Nursing Studies, 103840.<br>Balice-Bourgois, C., Zumstein-Shaha, M., Vanoni, F., Jaques, C., Newman, C. J., &	3 x parent/caregiver<br>5 x physician<br>1 x nurse<br>1 x respiratory therapist<br>1 x friend/family member<br>1 x physical therapist

© 2022 Gill PJ et al. *JAMA Network Open*.

		when discussing pain with children, what other methods are there compared to the 1-10 scale, what other options are available to teaching children about their pain or chronic condition?<br>education of patients how to assess pain and opioid	Simonetti, G. D. (2020). A systematic review of clinical practice guidelines for acute procedural pain on neonates. The Clinical journal of pain, 36(5), 390-398.<br>Ballard, A., Khadra, C., Adler, S., Doyon-Trottier, E., & Le May, S. (2018). Efficacy of the Buzzy® device for pain management of children during needle-related procedures: a systematic review protocol. Systematic reviews, 7(1), 1-7.<br>Dancel, R., Liles, E. A., & Fiore, D. (2017). Acute pain management in hospitalized children. Reviews on recent clinical trials, 12(4), 277-283.<br>Lavallée, A., Khadra, C., Ballard, A., Colson, S., & Aita, M. (2020). Systematic review and meta-analysis of olfactive stimulation interventions to manage procedural pain in preterm and full-term neonates. International Journal of Nursing Studies, 110, 103697-103697.<br>Dunlop, R., & Bennett, K. C. (2006). Pain management for sickle cell disease in children and adults. Cochrane Database of Systematic Reviews, (2).<br>Fernandes, A. M., De Campos, C., Batalha, L., Perdigão, A., & Jacob, E. (2014). Pain assessment using the Adolescent Pediatric Pain Tool: a systematic review. Pain Research and Management, 19(4), 212-218.<br>Liossi, C., Johnstone, L., Lilley, S., Caes, L., Williams, G., & Schoth, D. E. (2019). Effectiveness of interdisciplinary interventions in paediatric chronic pain management: a systematic review and subset meta-analysis. British journal of anaesthesia, 123(2), e359-e371.<br>Saramba, M. I., Shakya, S., & Zhao, D. (2020). Analgesic management of uncomplicated acute sickle-cell pain crisis in pediatrics: a systematic review and meta-analysis. Jornal de pediatria, 96(2), 142-158.<br>Tan, L. M. L., Leong, K. S. P., & Yip, W. K. (2014). Effective pain management during painful procedures in children with cancer: a systematic review. JBI Evidence Synthesis, 12(4), 430-484.<br>Yasmeen, I., Krewulak, K. D., Zhang, C., Stelfox, H. T., & Fiest, K. M. (2020). The effect of caregiver-facilitated pain management interventions in hospitalized patients on patient, caregiver, provider and health system outcomes: A systematic review. Journal of Pain and Symptom Management.<br><br>Verdict: Systematic review by Balice-Bourgois, et. al is the latest and one of the strongest evidences. The CPS statement also covers various aspects of pain management. **This question has been addressed in the evidence base, with a large number of high quality reviews and clinical practice guidelines.**	
3	What are effective methods (e.g. education) to prepare families for discharge from the general paediatric inpatient unit?	When can we go home?<br>What role would a step down unit for a rehab unit play in the transition home for a family in which their child acquired a significant disability - ie prior to discharge being in a near by apartment like setting in which the parent take full responsibility for care and the acute rehab team simulate community therapy and support.<br>need research and QI to minimize LOS and promote best practice in care and safe d/c home.<br>brain injury or spinal cord to ease the transition home.<br>What issues do they encounter after discharge that we should be addressing better during the inpatient stay?<br>waiting for family services to find a safe home for discharge etc...<br>-<br>All items above and communicating when rounds will be. follow up post release from hospital.<br>What are the perceptions of patients and families of their inpatient experience after they have settled at home?<br>What issues do they encounter after discharge that we should be addressing better during the inpatient stay?	CPS statement: CPS guidelines on educating parents prior to discharge for healthy infants;<br>Lemyre B, Jefferies AL, O'Flaherty P. Facilitating discharge from hospital of the healthy term infant. Paediatrics & child health. 2018 Nov 19;23(8):515-22.<br>Jefferies AL, Canadian Paediatric Society, Fetus and Newborn Committee. Going home: facilitating discharge of the preterm infant. Paediatrics & child health. 2014 Jan 10;19(1):31-6.<br>AAP guideline: Discusses the discharge process for high risk neonates only;<br>Committee on Fetus and Newborn. Hospital discharge of the high-risk neonate. Pediatrics. 2008 Nov;122(5):1119-26.<br>NICE guideline: Guideline on preparing for discharge for mental health practitioners for adults. No guidelines specific to children.<br>NICE. Transition between inpatient mental health settings and community or care home settings.<br><br>Hall, K. K., Petsky, H. L., Chang, A. B., & O'grady, K. F. (2018). Caseworker-assigned discharge plans to prevent hospital readmission for acute exacerbations in children with chronic respiratory illness. Cochrane Database of Systematic Reviews, (11).<br>Ronan, S., Brown, M., & Marsh, L. (2020). Parents' experiences of transition from	4 x parent/caregiver<br>1 x recreation therapist<br>6 x physician<br>7 x friend/family member<br>5 x nurse

© 2022 Gill PJ et al. *JAMA Network Open*.

		Readmission rates for respiratory illnesses like asthma, bronchiolitis.<br>Are there barriers to discharge that can be improved?<br>Optimal management of most common conditions, bronchiolitis, asthma, etc while maintaining efficiency, safety, patient satisfaction<br>It could have been better if they knew it was so urgent to get us a date when we were leaving so we could have planned as a family.<br>When will the next follow up be?<br>Transition to Home Discharge (Are patients really following our recommendations?<br>Lack of discharge planning.<br>Admission could have possibly been avoided had more education been provided to parents regarding intake/output/breastfeeding and topping up<br>My concerns on my childs stay after being discharged were recognizing the symptoms of an attack and knowing when to seek help again or when it was a situation we could control. My child had been admitted for pneumonia and asthma and after being discharged for few hours he was re-admitted.<br>nurses ability/preparedness to teach patients and families (assessing health literacy, adult learning principles))<br>Did they show up for follow as scheduled/recommended? family and patient education formats<br>Poor discharge planning<br>Best practices for providing patient education before discharge.<br>Prior to being put on the general ward one time my son had been in the icu and after no longer being sedated he was able to talk. Then after being put on a new med all of a sudden he couldn't talk and he lost his ability to use the toilet and had to wear pull ups. I was concerned about it on the general ward and they said it was just a side effect and it should go away. He got a referral to an outpatient clinic at medical day treatment to get checked over a few days after being discharged but still couldn't talk or use the toilet. They ended up diagnosing him with psychosis from the med through the neuro clinic but I had to deal with a giggling crazy kid that was laughing and throwing stuff and peeing on the cat. I don't think he should have been discharged. If he couldn't talk or lost his ability tobuse the toilet that should have been a red flag. It is also a golden opportunity to educate about a healthy lifestyle, once they are discharged, including the 4 Pillars of Health: sleep, nutrition, mental health and exercise.<br>When can we go home?<br>when we can safely bring her home?<br>When we left hospital they said they wanted to do surgery sometime in fall and now we are in fall but I haven heard anything even after several follow ups.<br>We have other kids also in care and no family here an specific date would have been better for arranging things.<br>Very little information about follow up and how that would work.	hospital to home of a child with complex health needs: A systematic literature review. Journal of Clinical Nursing, 29(17-18), 3222-3235.<br>Spittle, A., Orton, J., Anderson, P. J., Boyd, R., & Doyle, L. W. (2015). Early developmental intervention programmes provided post hospital discharge to prevent motor and cognitive impairment in preterm infants. Cochrane Database of Systematic Reviews, (11).<br><br>Verdict: Importance of discharge preparation has been raised in the systematic reviews, but little evidence has been found on the effective methods specific to the GPIU. The CPS and AAP discuss discharge planning but related to specific pediatric populations (e.g. term or pretern newborn infants). **This question has been partially addressed in the evidence base.**	
4	What educational content provided at discharge can support families and reduce hospital re-admissions	All items above and communicating when rounds will be.<br>Admission could have possibly been avoided had more education been provided to parents regarding intake/output/breastfeeding and topping up<br>Best practices for providing patient education before discharge.	CPS statement: No relevant guidelines.<br>AAP guideline: No relevant guidelines.<br>NICE guideline: No relevant guidelines.<br><br>Auger, K. A., Kenyon, C. C., Feudtner, C., & Davis, M. M. (2014). Pediatric hospital	3 x friend/family member<br>2 x nurse

© 2022 Gill PJ et al. *JAMA Network Open*.

	to the general paediatric inpatient unit?	Prior to being put on the general ward one time my son had been in the icu and after no longer being sedated he was able to talk. Then after being put on a new med all of a sudden he couldn't talk and he lost his ability to use the toilet and had to wear pull ups. I was concerned about it on the general ward and they said it was just a side effect and it should go away. He got a referral to an outpatient clinic at medical day treatment to get checked over a few days after being discharged but still couldn't talk or use the toilet. They ended up diagnosing him with psychosis from the med through the neuro clinic but I had to deal with a giggling crazy kid that was laughing and throwing stuff and peeing on the cat. I don't think he should have been discharged. If he couldn't talk or lost his ability tobuse the toilet that should have been a red flag. It is also a golden opportunity to educate about a healthy lifestyle, once they are discharged, including the 4 Pillars of Health: sleep, nutrition, mental health and exercise.<br>when we can safely bring her home?	discharge interventions to reduce subsequent utilization: a systematic review. Journal of hospital medicine, 9(4), 251-260.<br>Belanger, R., Leroux, D., & Lefebvre, P. (2021). Supporting caregivers of children born prematurely in the development of language: A scoping review. Paediatrics & Child Health, 26(1), e17-e24.<br>Coca, K. P., Pinto, V. L., Westphal, F., Mania, P. N. A., & Abrão, A. C. F. D. V. (2018). Bundle of measures to support Intrahospital exclusive breastfeeding: evidence of systematic reviews. Revista Paulista de Pediatria, 36(2), 214-220.<br>Curran, J. A., Gallant, A. J., Zemek, R., Newton, A. S., Jabbour, M., Chorney, J., ... & Campbell, S. G. (2019). Discharge communication practices in pediatric emergency care: a systematic review and narrative synthesis. Systematic reviews, 8(1), 83.<br>Hall, K. K., Petsky, H. L., Chang, A. B., & O'grady, K. F. (2018). Caseworker-assigned discharge plans to prevent hospital readmission for acute exacerbations in children with chronic respiratory illness. Cochrane Database of Systematic Reviews, (11).<br>Hamline, M. Y., Speier, R. L., Dai Vu, P., Tancredi, D., Broman, A. R., Rasmussen, L. N., ... & Li, S. T. T. (2018). Hospital-to-home interventions, use, and satisfaction: a meta-analysis. Pediatrics, 142(5).<br>Ronan, S., Brown, M., & Marsh, L. (2020). Parents' experiences of transition from hospital to home of a child with complex health needs: A systematic literature review. Journal of Clinical Nursing, 29(17-18), 3222-3235.<br><br>Verdict: Systematic review by Auger et al. is the strongest evidence that highlights the benefits of educational content, but it is related to asthma. Educational content at discharge have been identified by some studies, but it is limited to certain pediatric population (e.g. children with chronic illnesses). **This question has been partially addressed in the evidence base. Limited or no evidence related to discharge preparation to prevent hospital re-admission.**	
5	What best practices and/or care models exist for inpatient care for children and youth with medical complexity on the general paediatric inpatient unit?	For children with medical complexity (CMC), they should have an automatic pharmacist and dietician who reviews their care<br>For CMC, I would like to know if we can get feedback from parents on how we could have prevented or shorted the hospitalization stay; how we could have improved the stay (Patient/family oriented outcome measures)<br>How can weekend care be improved for medically complex children?<br>Does avoiding transfer out of NICU or PICU of medically complex children prevent medical errors or lead to better patient care?<br>Children with complexity now comprise a signficant proportion of inpatients - how has "traditional acute care" model been effectively modified to meet the needs of this acute/chronic cohort?<br>What is the best model of care to support children with neurocomplexities where there is no rulebook to follow?<br>Risks to infants and developmental care and management.<br>How do we communicate better with families and caregivers of CMC, particularly with respect to minimizing medical error and increasing quality of care?<br>complex care -best practice and transition common diagnosis common diagnoses are important and some questions remain<br>Most kids in hospital have complex conditions and less usual presentations.<br>How can children/youth with complex issues that combine mental	CPS statement: Guideline related to possible medication error and its prevention.<br>Huth K, Vandecruys P, Orkin J, Patel H. Medication safety for children with medical complexity. Paediatrics & child health. 2020 Nov;25(7):473-.<br>AAP guideline: Recognizes CMC as a special population but does not outline best practices or care models.<br>Kuo DZ, Houtrow AJ. Recognition and management of medical complexity. Pediatrics. 2016 Dec 1;138(6).<br>NICE guideline: No relevant guidelines.<br><br>Bradshaw, S., Bem, D., Shaw, K., Taylor, B., Chiswell, C., Salama, M., ... & Cummins, C. (2019). Improving health, wellbeing and parenting skills in parents of children with special health care needs and medical complexity–a scoping review. BMC pediatrics, 19(1), 1-11.<br><br>Verdict: Lack of evidence on best practices for CMC. **This question has not been addressed in the evidence base.**	1 x physician

© 2022 Gill PJ et al. *JAMA Network Open*.

		and physical health concerns be best cared for?<br>I would like to see questions about the standard of care for children with advanced technology needs (e.g. ventilators) on a general paediatrics ward. The practice is different across the country.<br>How can weekend care be improved for medically complex children?		
6	What best practices and/or care models exist around discharge for children and youth with medical complexity on the general paediatric inpatient unit?	For CMC, nurses and team should have training on how to discharge safely back to the community<br>For CMC, I would want a community pharmacy identified so that any new/changed medications are updated and ready to pick up at home.	CPS statement: No relevant guidelines<br>AAP guideline: No relevant guidelines<br>NICE guideline: No relevant guidelines<br><br>Mantler, T., Jackson, K. T., Baer, J., White, J., Ache, B., Shillington, K., & Ncube, N. (2020). Changes in Care-A Systematic Scoping Review of Transitions for Children with Medical Complexities. Current pediatric reviews, 16(3), 165-175.<br>An Integrated Telemedicine-Home Visitation Program to Increase Outcomes for Children With Medical Complexity https://clinicaltrials.gov/ct2/show/NCT03590509<br>Bradshaw, S., Bem, D., Shaw, K., Taylor, B., Chiswell, C., Salama, M., ... & Cummins, C. (2019). Improving health, wellbeing and parenting skills in parents of children with special health care needs and medical complexity–a scoping review. BMC pediatrics, 19(1), 1-11.<br>Breneol, S., Belliveau, J., Cassidy, C., & Curran, J. A. (2017). Strategies to support transitions from hospital to home for children with medical complexity: a scoping review. International journal of nursing studies, 72, 91-104.<br><br>Verdict: There are some evidences for potential interventions, but there is not clarity around which intervention should be implemented. The issue of medical complexity and their safe discharge has received attention, but concrete evidences are not available. **This question has been partially addressed in the evidence base.**	12 x physician<br>2 x nurse
7	What methods of communication are most effective between patients, caregivers and health care providers on a general paediatric inpatient unit?	Every interaction was very quick. The information would be relayed so quickly that I couldn't think of a response until after the physician/ nurse left the room.<br>Why do i only see my child's doctor randomly when I sit by their bedside? It is hard to be there all the time.<br>It would be great to do research that streamlines communication, or perhaps, enhance communication by being transparent with patients and families as to why you need to here the same information over and over, and how the information is used to inform care.<br>Would like to be told in advance about the risks of Botox with green tendon transfer Also No one could explain why hair on the arm under the cast grew so long<br>How can we decrease hospital duration in medically complex children by coordinating care by multiple specialists in a more timely manner?<br>I feel that there needs to be better communication between healthcare professionals in regards to their patients.<br>What are the barriers and enablers to providing assessment, treatment planning and the communication is this to the patient +/- family in an integrated ways?<br>Communication between HCPS and patients and families<br>Would care be better and would families learn more from informal interactions with other families if children with similar conditions were grouped together-even if all on a general paeds unit?	CPS statement: Guidelines are available (using interpreters for cross-cultural communication)<br>Ladha T, Zubairi M, Hunter A, Audcent T, Johnstone J. Cross-cultural communication: Tools for working with families and children. Paediatrics & child health. 2018 Feb 15;23(1):66-9.<br>Williams RC, Biscaro A, Clinton J. Relationships matter: How clinicians can support positive parenting in the early years. Paediatrics & child health. 2019 Jul 25;24(5):340-7.<br>Hendson L, Davies D. Supporting and communicating with families experiencing a perinatal loss. Paediatrics & child health. 2018 Nov 19;23(8):549-.<br>AAP guideline: Guidelines are available.<br>Levetown M. Communicating with children and families: from everyday interactions to skill in conveying distressing information. Pediatrics. 2008 May 1;121(5):e1441-60.<br>NICE guideline: No relevant guidelines.<br><br>Xafis, V., Wilkinson, D., & Sullivan, J. (2015). What information do parents need when facing end-of-life decisions for their child? A meta-synthesis of parental feedback. BMC palliative care, 14(1), 1-11.<br>Chan, H. K., Hassali, M. A., Lim, C. J., Saleem, F., & Tan, W. L. (2015). Using pictograms to assist caregivers in liquid medication administration: a systematic review. Journal of clinical pharmacy and therapeutics, 40(3), 266-272.<br>Brett, J., Staniszewska, S., Newburn, M., Jones, N., & Taylor, L. (2011). A systematic mapping review of effective interventions for communicating with, supporting and providing information to parents of preterm infants. BMJ open, 1(1).	12 x friend/family<br>6 x parent/caregiver<br>5 x physcian<br>3 x nurse

© 2022 Gill PJ et al. *JAMA Network Open*.

		How does a community doctor knows when their patient is hospitalized and how can they best align their care in continuity with the hospital care?<br>More opportunities to talk to doctors<br>I Always found that we were asked the same questions over and over again, and it seemed that no one was communicating information with one another or that the information that was communicated wasn't trusted.<br>Miscommunication leading to longer hospital stays, incorrect treatments, unsafe patient care, frustrated families and inefficient care.<br>how to communicate with families in a teaching environment that does not add anxiety for the family or the learner.<br>Why certain antibiotics were given?<br>Why NICU nurses weren't putting the iv in my 4 week old?<br>Parents need time to ask questions and kids need patience in their treatments to make experiences less traumatic<br>Who can we contact if her condition changes overnight?<br>Who can we contact if we have a question about meds?<br>Why do i only see my child's doctor randomly when I sit by their bedside? It is hard to be there all the time.<br>I would like to know how my child will be cared for by the hospital staff. I would like to know when to expect a doctor will see my child during their stay. So often, a doctor will do their "rounds" just at the exact moment a parent leaves the room for 5 minutes. In that time period, the doctor comes and goes and is missed by the parent. Then the parent has no opportunity to speak to the doctor. I would be most helpful for the parents to have a schedule knowing when doctors do their rounds.<br>why can some kids walk around the hospital while admitted and others can't<br>When a wheelchair is brought to the room when being discharged is it normal practice to let the child walk from the bed to the wheelchair? This happened to my son before and he fell. The person who brought the wheelchair just watched him fall while I tried to grab him.<br>Sharing order sets for common pediatric conditions (i.e. Kawasaki Disease, Sickle Cell, Sepsis, etc). --> NOT RELATED<br>Which decisions made by paediatricians are evidenced-based and which are not?<br>Why ultrasounds were taken?	Henderson, A., Young, J., Herbert, A., Bradford, N., & Pedersen, L. A. (2017). Preparing pediatric healthcare professionals for end-of-life care discussions: an exploratory study. Journal of palliative medicine, 20(6), 662-666.<br>Oja, C., Edbom, T., Nager, A., Månsson, J., & Ekblad, S. (2020). Informing children of their parent's illness: A systematic review of intervention programs with child outcomes in all health care settings globally from inception to 2019. PloS one, 15(5), e0233696.<br>Ekberg, S., Bradford, N., Herbert, A., Danby, S., & Yates, P. (2015). Healthcare users' experiences of communicating with healthcare professionals about children who have life-limiting conditions: a qualitative systematic review protocol. JBI Evidence Synthesis, 13(11), 33-42.<br>Gentles, S. J., Lokker, C., & McKibbon, K. A. (2010). Health information technology to facilitate communication involving health care providers, caregivers, and pediatric patients: a scoping review. Journal of medical Internet research, 12(2), e22.<br>Ye, J., Rust, G., Fry-Johnson, Y., & Strothers, H. (2010). E-mail in patient–provider communication: A systematic review. Patient education and counseling, 80(2), 266-273.<br>Goyal, A. A., Tur, K., Mann, J., Townsend, W., Flanders, S. A., & Chopra, V. (2017). Do Bedside Visual Tools Improve Patient and Caregiver Satisfaction? A Systematic Review of the Literature. Journal of hospital medicine, 12(11), 930-936.<br><br>Verdict: There are several interventions related to effective communication that have been identified by Brett et al. but they are limited to certain pediatric populations (e.g. pre-term infants). There are some principles that could be implemented, but overall it is not clear which method is truly effective in the GPIU. **This question has been partially addressed in the evidence base**.	
8	Do communication systems or tools that connect healthcare providers on the general paediatric inpatient unit with community providers improve care of patients?	How do we connect community providers better to these wards as follow up care<br>We need to do better in communication with community professionals who are part of the child's health sphere.<br>How can we communicate more effectively as a health care team for seamless transition to the community?<br>How well to we communicate with primary care providers and could there be an intervention to do better	CPS statement: No unique system was mentioned other than use of interpreters.<br>Ladha T, Zubairi M, Hunter A, Audcent T, Johnstone J. Cross-cultural communication: Tools for working with families and children. Paediatrics & child health. 2018 Feb 15;23(1):66-9.<br>AAP guideline: Guidelines on the use of telemedicine and audiotapes<br>Levetown M. Communicating with children and families: from everyday interactions to skill in conveying distressing information. Pediatrics. 2008 May 1;121(5):e1441-60.<br>NICE guideline: No relevant guidelines.<br><br>Gentles, S. J., Lokker, C., & McKibbon, K. A. (2010). Health information technology to facilitate communication involving health care providers, caregivers, and pediatric patients: a scoping review. Journal of medical Internet research, 12(2), e22.	2 x physician<br>1 x occupational therapist

© 2022 Gill PJ et al. *JAMA Network Open*.

			Ye, J., Rust, G., Fry-Johnson, Y., & Strothers, H. (2010). E-mail in patient–provider communication: A systematic review. Patient education and counseling, 80(2), 266-273.<br>Goyal, A. A., Tur, K., Mann, J., Townsend, W., Flanders, S. A., & Chopra, V. (2017). Do Bedside Visual Tools Improve Patient and Caregiver Satisfaction? A Systematic Review of the Literature. Journal of hospital medicine, 12(11), 930-936.<br>Chan, H. K., Hassali, M. A., Lim, C. J., Saleem, F., & Tan, W. L. (2015). Using pictograms to assist caregivers in liquid medication administration: a systematic review. Journal of clinical pharmacy and therapeutics, 40(3), 266-272.<br>Brett, J., Staniszewska, S., Newburn, M., Jones, N., & Taylor, L. (2011). A systematic mapping review of effective interventions for communicating with, supporting and providing information to parents of preterm infants. BMJ open, 1(1).<br><br>Verdict: Some communication systems have been identified, but none of them seem to be designed to connect inpatient healthcare providers with community providers. **This question has been not been addressed in the evidence base.**	
9	What are the most effective communication methods (e.g. handover, rounds, etc.) between healthcare providers on a general paediatric inpatient unit?	lack of communication between specialists<br>lack of communication between nursing staff post surgery and specialist team<br>Many! There is a very big lack of communication between doctors and nurses. Nurses at the same time don't communicate effectively to parents and just give orders. When it comes to children, surgery and anesthesia it is extremely important to educate the parent and build a plan together as per meals including water and other drinks. Nurses don't like to give information to parents and it's cruel to ask a parent not to feed their children for days specially when they still breast feed and are in pain.<br>Lack of professional communication between staff RN to RN and also to other members of health care teams and disciplines<br>The nurses were incredible. They obviously cared greatly for our child and what she was going through. They were the biggest source of information for us throughout the week we were staying in hospital, and made sure we understood everything they were doing, and why. They explained everything they were doing and WHY, often detailing different research information while explaining things to us.<br>How efficient is Epic compared to other systems used in hospitals?<br>How can we utilize connect care for communication between teams?<br>Since Covid, opportunities for interprofessional discussion at rounds is more difficult. Is there a way to improve this?<br>How can we have better processes/structures to ensure better flow of information (between staff and families) throughout a hospital stay and beyond (after discharge)?<br>Longitudinal discussion can help clarify what works/doesn't work in real life and help incorporate new evidence where applicable multiD handover and rounds how it is run and the best way to run to incorp family centered care<br>Why do most centres claim to offer "family centred care" but spend most of their time speaking for and about patients on rounds?<br>What is parent's perspective of bedside nursing handover?	CPS statement: No relevant guidelines.<br>AAP guideline: No relevant guidelines.<br>NICE guideline: No relevant guidelines.<br><br>Brett, J., Staniszewska, S., Newburn, M., Jones, N., & Taylor, L. (2011). A systematic mapping review of effective interventions for communicating with, supporting and providing information to parents of preterm infants. BMJ open, 1(1).<br><br>Verdict: Did not find evidence that looks at communication between provider teams in the GPIU. While there is some evidence related to the IPASS handover system, there are no systematic reviews that include this tool. **This question has not been addressed in the evidence base**	3 x friend/family member<br>2 x nurse<br>1 x parent/caregiver<br>1 x youth<br>1 x occupational therapist<br>4 x physician
10	What communication or information sharing methods are effective	Most tests are not explained before they are done. What are they looking for? What could be the implications? An x-ray machine was wheeled into our room, but no one had told us it was coming,	CPS statement: No relevant guidelines.<br>AAP guideline: No relevant guidelines.<br>NICE guideline: No relevant guidelines.	2 x parent/caregiver<br>1 x nurse

© 2022 Gill PJ et al. *JAMA Network Open*.

	at explaining tests to patients and caregivers on the general paediatric inpatient unit?	or why. When the results were received, we were only told it was "clear", but never informed as to what they were looking for.<br>Tests should be explained before they happen, in order to prepare the family ahead of time, especially those who have children who will have questions themselves. If our baby hadn't been pre-verbal, explaining why the giant machine was there to look at her insides with no information would've been quite difficult, and possibly scary.<br>Most of the doctors were very quickly in and out, with little time for explanation, except to tell us they'd ordered tests. Only one stayed to give us a more in-depth reason as to the testing they were requesting, and what it might mean for our baby's health in future. This took all of five minutes, but was incredibly reassuring.<br>Due to poor communication, there have been unnecessary tests ordered, delays in patient care and poor plan of care execution.<br>Many! There is a very big lack of communication between doctors and nurses. Nurses at the same time don't communicate effectively to parents and just give orders. When it comes to children, surgery and anesthesia it is extremely important to educate the parent and build a plan together as per meals including water and other drinks. Nurses don't like to give information to parents and it's cruel to ask a parent not to feed their children for days specially when they still breast feed and are in pain.<br>I felt some doctors/nurses would explain why certain test were done or what my child's Illness was exactly. I always felt like I was rushed and couldn't ask my questions.	Verdict: Evidence is limited to communication around testing. **This question has not been addressed in the evidence base**	1 x friend/family member
11	What is the most effective route (i.e. IV vs PO) and duration of antibiotic therapy for children and youth with bacteremia (of known origin e.g. UTI vs unknown source) on a general paediatric inpatient unit?	duration of treatment for neonatal UTI<br>Treatment of neonatal febrile UTI. IV vs PO and duration of treatment<br>2) Use of oral antibiotics in neonates with urinary tract infections<br>Treatment for infants with bacteremic UTI<br>Duration of intravenous antibiotics for infant under 30 days of ager with urinary tract infection.<br>UTI prophylaxis - still wide variation in practice especially between urology and pediatrics - who needs prophylaxis?<br>2) Use of oral antibiotics in neonates with urinary tract infections<br>Treatment of neonatal febrile UTI. IV vs PO and duration of treatment<br>duration of IV/total Rx for pyelo<br>duration of treatment for neonatal UTI	CPS statement: Management guidelines focused on optimum antibiotic use<br>Gerber JS, Jackson MA, Tamma PD, Zaoutis TE, COMMITTEE ON INFECTIOUS DISEASES. Antibiotic stewardship in pediatrics. Pediatrics. 2021 Jan 1;147(1).<br>Le Saux N, Canadian Paediatric Society, Infectious Diseases and Immunization Committee. Antimicrobial stewardship in daily practice: Managing an important resource. Paediatrics & child health. 2014 May 1;19(4):261-5.<br>Le Saux N, Robinson JL, Canadian Paediatric Society, Infectious Diseases and Immunization Committee. Uncomplicated pneumonia in healthy Canadian children and youth: practice points for management. Paediatrics & child health. 2015 Dec 4;20(8):441-5.<br>AAP guideline: Specific instructions suggesting oral over IV antibiotic therapy (for CAP)<br>Kronman MP, Hersh AL, Newland JG, Gerber JS. Getting over our inpatient oral antibiotic aversion. Pediatrics. 2018 Dec 1;142(6).<br>NICE guideline: No relevant guidelines.<br><br>Havey, T. C., Fowler, R. A., & Daneman, N. (2011). Duration of antibiotic therapy for bacteremia: a systematic review and meta-analysis. Critical Care, 15(6), 1-11.<br><br>Verdict: Evidence is limited to specific clinical conditions; the overall evidence is unclear. Systematic review by Havey et al, is the best available evidence. **This question has been partially addressed in the evidence base**	6 x physician
12	What is the most effective route (i.e. IV vs PO) and duration of antibiotic therapy for urinary tract infections (UTIs) with and	duration of treatment for neonatal UTI<br>Treatment of neonatal febrile UTI. IV vs PO and duration of treatment<br>2) Use of oral antibiotics in neonates with urinary tract infections<br>Treatment for infants with bacteremic UTI<br>Duration of intravenous antibiotics for infant under 30 days of ager with urinary tract infection.	CPS statement: No significant difference between IV and PO antibiotics; no difference between short and along-course antibiotics (> 2 months of age)<br>Robinson JL, Finlay JC, Lang ME, Bortolussi R, Canadian Paediatric Society, Community Paediatrics Committee, Infectious Diseases and Immunization Committee. Prophylactic antibiotics for children with recurrent urinary tract infections. Paediatrics & child health. 2015 Feb 6;20(1):45-7.<br>Robinson JL, Finlay JC, Lang ME, Bortolussi R, Canadian Paediatric Society,	9 x physician

© 2022 Gill PJ et al. *JAMA Network Open*.

	without a negative blood culture?	UTI prophylaxis - still wide variation in practice especially between urology and pediatrics - who needs prophylaxis?<br>2) Use of oral antibiotics in neonates with urinary tract infections<br>Treatment of neonatal febrile UTI. IV vs PO and duration of treatment<br>duration of IV/total Rx for pyelo<br>duration of treatment for neonatal UTI	Community Paediatrics Committee, Infectious Diseases and Immunization Committee. Urinary tract infection in infants and children: Diagnosis and management. Paediatrics & child health. 2014 Jun 13;19(6):315-9.<br>AAP guideline: Clinician should base the choice of route of administration on practical considerations: initiating treatment orally or parenterally is equally efficacious.<br>Subcommittee on Urinary Tract Infection. Reaffirmation of AAP clinical practice guideline: the diagnosis and management of the initial urinary tract infection in febrile infants and young children 2-24 months of age. Pedatrics. 2016 Dec; 138(6):e20163026<br>NICE guideline: Recommends VI for <3 months; PO as first line for > 3 months, if vomiting exists then IV. Recommends 7-10-day antibiotic course also identifies that no difference between IV and PO routes.<br>National Institute for Clinical Excellence. Pyelonephritis (acute): antimicrobial prescribing. England: Public Health England. 2018.<br><br>Fitzgerald, A., Mori, R., Lakhanpaul, M., & Tullus, K. (2012). Antibiotics for treating lower urinary tract infection in children. Cochrane Database of Systematic Reviews, (8).<br>Nickavar, A., & Sotoudeh, K. (2011). Treatment and prophylaxis in pediatric urinary tract infection. International journal of preventive medicine, 2(1), 4.<br>Sutton, A. G., Chandler, N., & Roberts, K. B. (2017). Recent studies on the care of first febrile urinary tract infection in infants and children for the pediatric hospitalist. Reviews on recent clinical trials, 12(4), 269-276.<br>Havey, T. C., Fowler, R. A., & Daneman, N. (2011). Duration of antibiotic therapy for bacteremia: a systematic review and meta-analysis. Critical Care, 15(6), 1-11.<br>Schroeder, A. R., Lucas, B. P., Garber, M. D., McCulloh, R. J., Joshi-Patel, A. A., Neuman, M. I., & Chaudhari, P. P. Diagnosis and Management of UTI in Febrile Infants Age 0–2 Months: Applicability of the AAP Guideline.<br><br>Verdict: The strongest evidence in this area is likely to be the study by Fitzgeral et al - this is a systematic review currently registered with Cochrane. Uncertainty related to less than 2 months of age for both positive and negative blood culture; iover 2 months of age with positive blood culture. **This question has been partially addressed in the evidence base.**	
13	What is the most effective way to use oxygen monitoring (e.g. intermittent, continuous) for hospitalized children with common respiratory illnesses (e.g. bronchiolitis) on the general peadiatric inpatient unit?	Continuous oximetry monitoring has become commonplace on the pediatric ward. Kids move around a lot and there can be a lot of false 'desats' and these can lead to alarm fatigue and missed real 'desats.' I'm curious about whether routine continuous oximetry is overused on the general pediatric ward. We know from ICU experience that it can lead to alarm fatigue (especially evidence in NICU.)<br>Should this target be different on admission, during signs of significant respiratory distress, and later, when hospital discharge is considered? Is it 95%, 92%, 90%?<br>Also there is a huge discrepancy in SpO2 monitoring with the current patient room monitors vs the model used for overnight oximetries. This was brought to Respirology's attention. look at the procurement of hospital equipment ...does it involve the right professions for feedback at purchase time. An example .... new ventilators just recently purchased .... is cheaper always better ????<br>Are there adverse events related to alarm fatigue where we would be better off using continuous SpO2 monitoring more judiciously?<br>What is the oxygen saturation to aim for in the case of acute respiratory failure (acute viral bronchiolitis in infants, pneumonia,	CPS statement: Topic of continuous versus intermittent monitoring of oxygen saturation is controversial, with lack of clear recommendations.<br>Friedman JN, Rieder MJ, Walton JM, Canadian Paediatric Society, Acute Care Committee, Drug Therapy and Hazardous Substances Committee. Bronchiolitis: recommendations for diagnosis, monitoring and management of children one to 24 months of age. Paediatrics & child health. 2014 Nov 3;19(9):485-91.<br>AAP guideline: Clinicians may choose not to use continuous pulse oximetry for infants and children with a diagnosis of bronchiolitis (Evidence Quality: C; Recommendation Strength: Weak Recommendation [based on lower level evidence]).<br>Ralston SL, Lieberthal AS, Meissner HC, Alverson BK, Baley JE, Gadomski AM, Johnson DW, Light MJ, Maraqa NF, Mendonca EA, Phelan KJ. Clinical practice guideline: the diagnosis, management, and prevention of bronchiolitis. Pediatrics. 2014 Nov 1;134(5):e1474-502.<br>NICE guideline: No relevant guidelines.<br><br>Verdict: Could not find any relevant systematic reviews that addresses this query. Checked references from CPS statement and AAP guideline, but references are either outdated or inconclusive. Also checked a recently published clinical trial (Mahant et al. 2021) but no systematic review.	4 x respiratory therapist<br>5 x physician<br>1 x nurse

© 2022 Gill PJ et al. *JAMA Network Open*.

		asthma attack, ...<br>Are we intervening before adverse events because of this monitoring?<br>Do the monitors get over used?<br>Intermittent vs continuous oxygen use<br>When does a child really need to be given oxygen as an intervention for desaturation - different views by physicians.<br>Readmission rates for respiratory illnesses like asthma, bronchiolitis.<br>Optimal management of most common conditions, bronchiolitis, asthma, etc while maintaining efficiency, safety, patient satisfaction	Mahant S, Wahi G, Giglia L, Pound C, Kanani R, Bayliss A, Roy M, Sakran M, Kozlowski N, Breen-Reid K, Lavigne M. Intermittent versus continuous oxygen saturation monitoring for infants hospitalised with bronchiolitis: study protocol for a pragmatic randomised controlled trial. BMJ open. 2018 Apr 1;8(4):e022707.<br>**his question has not been addressed in the evidence base.**	
14	What is the most effective way to wean respiratory support (e.g. oxygen, heated high flow nasal cannula) in hospitalized children and youth with respiratory illnesses (e.g. bronchiolitis, asthma) on a general paediatric inpatient unit?	4. Standardizing weaning of O2: we all too frequently leave O2 prongs on the child/infant when the O2 has been turned off. This increases resistance to breathing which may decrease the likelihood that they will manage well on room air. Could we test weaning with and without the prongs on a see if it makes a difference?	CPS statement: No relevant guidelines.<br>AAP statement: Discussed weaning from continuous positive airway pressure (CPAP) to heated high flow nasal cannula (HHFNC) but no specific recommendations on weaning from HFFNC.<br>Cummings JJ, Polin RA. Noninvasive respiratory support. Pediatrics. 2016 Jan 1;137(1).<br>NICE guideline: No relevant guidelines.<br><br>Morris, L., Cook, N., Ramsey, A., Alacapa, J. V., Smith, L. E., Gray, C., ... & Christensen, M. (2020). Weaning humidified high flow oxygen therapy among paediatric patients: an integrative review of literature. Journal of pediatric nursing, 50, 37-45.<br>Rose, L., Schultz, M. J., Cardwell, C. R., Jouvet, P., McAuley, D. F., & Blackwood, B. (2013). Automated versus non-automated weaning for reducing the duration of mechanical ventilation for critically ill adults and children. Cochrane Database of Systematic Reviews, (6).<br>van Delft, B., Van Ginderdeuren, F., Lefevere, J., van Delft, C., & Cools, F. (2020). Weaning strategies for the withdrawal of non-invasive respiratory support applying continuous positive airway pressure in preterm infants: a systematic review and meta-analysis. BMJ paediatrics open, 4(1).<br><br>Verdict: The evidence available is based on ICU level care and not the GPIU or inpatient ward; limited evidence to answer the question. **This question has NOT been addressed in the evidence base**	1 x physical therapist
15	What is the most effective way (i.e. population, illness, initiation, weaning) to utilize heated high flow nasal cannula (HHFNC) in hospitalized children and youth on the general paediatric inpatient unit?	High Flow Nasal Cannula needs a closer look as there is belief that due to a lack of RT resources able to attend rounds, there is a delay in weaning of O2 and transition to Nasal prongs to room air....again not cost effective and delay in patient discharge.<br>who benefits from high flow	CPS: There is one guideline available that outlines some instructions on how to use heated high flow nasal cannula (HHFNC) effectively.<br>Chauvin-Kimoff L, DeCaen A. Use of high-flow nasal cannula oxygen therapy in infants and children. Paediatrics & child health. 2018 Nov 19;23(8):555-.<br>AAP: No relevant guidelines. only one paper that looked at HFNC practices in pediatric ward<br>Kalburgi S, Halley T. High-flow nasal cannula use outside of the ICU setting. Pediatrics. 2020 Nov 1;146(5).<br>NICE: No relevant guidelines.<br><br>Beggs, S., Wong, Z. H., Kaul, S., Ogden, K. J., & Walters, J. A. (2014). High-flow nasal cannula therapy for infants with bronchiolitis. Cochrane Database of Systematic Reviews, (1).<br>Lin, J., Zhang, Y., Xiong, L., Liu, S., Gong, C., & Dai, J. (2019). High-flow nasal cannula therapy for children with bronchiolitis: a systematic review and meta-analysis. Archives of disease in childhood, 104(6), 564-576.<br>Moreel, L., & Proesmans, M. (2020). High flow nasal cannula as respiratory support in treating infant bronchiolitis: a systematic review. European journal of pediatrics, 179(5), 711-718.	1 x respiratory therapist<br>1 x physician

			Ma, L., Liu, C. Q., Gu, X. H., & Liu, X. J. (2014). The efficacy and safety of heated humidified high-flow nasal cannula for prevention of extubation failure in neonates. J Matern Fetal Neonatal Med, 27, 208-209. (ARTICLE IN CHINESE)<br>Feng, Z. T., Yang, Z. M., Gu, D. F., & Yang, X. L. (2015). Clinical efficacy of heated humidified high-flow nasal cannula in preventing extubation failure in neonates: a meta analysis. Zhongguo Dang dai er ke za zhi= Chinese Journal of Contemporary Pediatrics, 17(12), 1327-1332. (ARTICLE IN CHINESE)<br><br>Verdict: Studies identified focused on effectiveness of HHFNC and not on why it is effective, or which way is most effective. Evidence is present but not sufficient to answer the question. **This question has NOT been addressed in the evidence base.**	
16	What is the most appropriate way to provide fluid therapy (e.g. IV, nasogastric tube) to children and youth hospitalized with bronchiolitis on a general paediatric inpatient unit?	How long should an infant with bronchiolitis have sats >= 90 on room air prior to discharge, to prevent readmission - one night, one nap, 4 hours, 12 hours?<br>What is the most appropriate way to provide fluids to hospitalized children with bronchiolitis?<br>2. Bronchiolitis care: is NG hydration superior to IV hydration? Seems plausible that it should be preferred (generally fewer attempts, less hyponatremia, no need to do daily lytes and provides calories). But is it better, or at least non-inferior to IV? (in terms of clinical outcomes, LOS, weight loss during hospital stay, etc)<br>The effectiveness of hypertonic saline in secretion clearance of bronchiolitis. Is this an unnecessary step?<br>Are there negative outcomes associated with the use of nebulized bronchodilators in treatment of children admitted for acute bronchiolitis?<br>Also there is a huge discrepancy in SpO2 monitoring with the current patient room monitors vs the model used for overnight oximetries. This was brought to Respirology's attention. look at the procurement of hospital equipment ...does it involve the right professions for feedback at purchase time. An example .... new ventilators just recently purchased .... is cheaper always better ????	CPS: Bronchiolitis guideline available which states that both routes of fluid therapy equally effective.<br>Ralston SL, Lieberthal AS, Meissner HC, Alverson BK, Baley JE, Gadomski AM, Johnson DW, Light MJ, Maraqa NF, Mendonca EA, Phelan KJ. Clinical practice guideline: the diagnosis, management, and prevention of bronchiolitis. Pediatrics. 2014 Nov 1;134(5):e1474-502.<br>AAP: Guideline available, but does not clarify which route is superior.<br>Friedman JN, Rieder MJ, Walton JM, Canadian Paediatric Society, Acute Care Committee, Drug Therapy and Hazardous Substances Committee. Bronchiolitis: recommendations for diagnosis, monitoring and management of children one to 24 months of age. Paediatrics & child health. 2014 Nov 3;19(9):485-91.<br>NICE: Guideline suggests using enteral tube fluid, but if not possible then parental fluids.<br>https://www.nice.org.uk/guidance/ng9<br><br>Gill, P. J., Parkin, P., & Mahant, S. (2020). Parenteral versus enteral fluid therapy for children hospitalised with bronchiolitis. Cochrane Database of Systematic Reviews, (3). [protocol]<br><br>Verdict: There are no systematic reviews, only 1 published protocol. **This question has NOT been addressed in the evidence base.**	2 x physician<br>2 x nurse<br>1 x respiratory therapist
17	What is the most effective route (i.e. IV vs PO), optimal indications, duration of antibiotic therapy for children and youth hospitalized with cellulitis on a general paediatric inpatient unit?	Early step down to oral in cellulitis.<br>How long should children with orbital cellulitis treated with IV antibiotics?<br>2) Duration of antibiotics for almost every infection we commonly treat (e.g. pneumonia, osteomyelitis/septic arthritis, UTI, mastoiditis, orbital/periorbital cellulitis, other skin/soft tissue infections (e.g. adenitis))<br>Prolonged courses of antibiotics when there is no evidence to the contrary.<br>Broadly, under what circumstances can antibiotics safely be minimized or withheld?<br>Optimal treatment durations.	CPS: Guideline focused on management of methacillin resistant Staph aureus (MRSA)<br>Robinson JL, Salvadori MI, Canadian Paediatric Society, Infectious Diseases and Immunization Committee. Management of community-associated methicillin-resistant Staphylococcus aureus skin abscesses in children. Paediatrics & child health. 2011 Feb 1;16(2):115-6.<br>AAP: No relevant guidelines. The one found is specific to orbital cellulitis<br>Seltz LB, Smith J, Durairaj VD, Enzenauer R, Todd J. Microbiology and antibiotic management of orbital cellulitis. Pediatrics. 2011 Mar 1;127(3):e566-72.<br>NICE: No relevant guidelines.<br><br>Verdict: There are no systematic reviews. The only relevant evidence found was a guideline specific to orbital cellulitis. The gudieline also doesn't clarify if IV or PO is the effective route . **The question has NOT been ddressed in the evidence base.**	6 x physician
18	What are effective treatments for children and youth hospitalized with an acute asthma exacerbation on a general paediatric inpatient unit?	more efficatious medications for asthma relief;<br>Duration of oral corticosteroïdes for patients admitted for acute asthma exacerbation<br>Withdrawal protocol for administration of ventolin during asthma exacerbation attacks.<br>Several: - treatment of asthma -<br>Best Treatments for Asthma Screening<br>new asthma guidelines.<br>Some patient care pathways (ex. asthma pathway used at CHEO)	CPS statement: Available, but no specific recommendations focused on inpatient management; mainly focuses on ED care.<br>Ortiz-Alvarez O, Mikrogianakis A, Canadian Paediatric Society, Acute Care Committee. Managing the paediatric patient with an acute asthma exacerbation. Paediatrics & child health. 2012 May 1;17(5):251-5.<br>AAP guideline: No relevant guideline.<br>NICE guideline: People admitted to hospital with an acute exacerbation of asthma should have a structured review by a member of a specialist respiratory team before discharge. No other details have been provided.	2 x friend/family member<br>4 x physician<br>2 x nurse

		can be difficult to understand and follow, especially for new nurses, and sometimes this leads to improper treatment and increased time for recovery/discharge.<br>my son has asthma and has been hospitalized many times with a need for steroids and oxygen. There are many exercises that staff try to get him to do to open his lungs. The most I have ever seen him improve though, due to laughter, and opening his lungs, was when child life specialists or the clown have come and played in a silly way. Are there studies that support the use of these programs? I firmly believe that they had a large impact on the speed of my son's recovery.	National Institute for Health and Care Excellence (Great Britain). Asthma: diagnosis, monitoring and chronic asthma management. National Institute for Health and Care Excellence (NICE); 2017.<br><br>Albuali, W. H. (2014). The use of intravenous and inhaled magnesium sulphate in management of children with bronchial asthma. The Journal of Maternal-Fetal & Neonatal Medicine, 27(17), 1809-1815.<br>Castro-Rodriguez, J. A., Pincheira, M. A., Escobar-Serna, D. P., Sossa-Briceño, M. P., & Rodriguez-Martinez, C. E. (2020). Adding nebulized corticosteroids to systemic corticosteroids for acute asthma in children: A systematic review with meta-analysis. Pediatric Pulmonology, 55(10), 2508-2517.<br>Gray, C. S., Powell, C. V., Babl, F. E., Dalziel, S. R., & Craig, S. (2019). Variability of outcome measures in trials of intravenous therapy in acute severe paediatric asthma: a systematic review. Emergency Medicine Journal, 36(4), 225-230.<br>Jat, K. R., & Chawla, D. (2012). Ketamine for management of acute exacerbations of asthma in children. Cochrane Database of Systematic Reviews, (11). (insufficient evidence)<br>Lin, C. Y., Yeh, T. L., Liu, S. J., Lin, H. H., Cheng, Y. J., Hung, H. H., ... & Lei, W. T. (2018). Effects of macrolide treatment during the hospitalization of children with childhood wheezing disease: a systematic review and meta-analysis. Journal of clinical medicine, 7(11), 432. (insufficient evidence)<br>Normansell, R., Sayer, B., Waterson, S., Dennett, E. J., Del Forno, M., & Dunleavy, A. (2018). Antibiotics for exacerbations of asthma. Cochrane Database of Systematic Reviews, (6). (insufficient evidence)<br>Rodrigo, G. J., & Castro-Rodriguez, J. A. (2014). Heliox-driven β2-agonists nebulization for children and adults with acute asthma: a systematic review with meta-analysis. Annals of Allergy, Asthma & Immunology, 112(1), 29-34.<br>Sawanyawisuth, K., Chattakul, P., Khamsai, S., Boonsawat, W., Ladla, A., Chotmongkol, V., ... & Sawanyawisuth, K. (2020). Role of inhaled corticosteroids for asthma exacerbation in children: An updated meta-analysis. Journal of Emergencies, Trauma, and Shock, 13(2), 161.<br>Travers, A. H., Jones, A. P., Camargo Jr, C. A., Milan, S. J., & Rowe, B. H. (2012). Intravenous beta 2-agonists versus intravenous aminophylline for acute asthma. Cochrane Database of Systematic Reviews, (12). (insufficient evidence)<br>Watts, K., & Chavasse, R. J. (2012). Leukotriene receptor antagonists in addition to usual care for acute asthma in adults and children. Cochrane Database of Systematic Reviews, (5).<br><br>Verdict: Multiple Cochrane systematic reviews were completed but each look at a different aspect of treatment, with limited evidence focused on the GPIU. **The question has been partially addressed in the evidence base.** [Since, management of acute asthma has multiple steps and most of the evidence is limited to the ED, it is difficult to conclude the status of the evidence.]	
19	Are additional doses of corticosteroids effective in hospitalized children and youth with croup on a general paediatric inpatient unit?	Treatment of a patient with croup hospitalized in general pediatrics (after first dose of steroid;?	CPS: Guideline discussed first dose of corticosteroid (ED based management)<br>Ortiz-Alvarez O. Acute management of croup in the emergency department. Paediatrics & child health. 2017 Jun 1;22(3):166-9.<br>AAP: Guideline discussed only single dose of corticosteroid.<br>Malhotra A, Krilov LR. Viral croup. Pediatrics in review. 2001 Jan 1;22(1):5-12.<br>NICE: No relevant guidelines.<br><br>Russell KF, Liang Y, O'Gorman K, Johnson DW, Klassen TP. Glucocorticoids for croup. Cochrane Database of Systematic Reviews. 2011(1).<br>Roked F, Atkinson M, Hartshorn S. G95 (P) Best practice: one or two doses of dexamethasone for the treatment of croup?.<br>Gates A, Gates M, Vandermeer B, Johnson C, Hartling L, Johnson DW, Klassen TP.	1 x physician

© 2022 Gill PJ et al. *JAMA Network Open*.

			Glucocorticoids for croup in children. Cochrane Database of Systematic Reviews. 2018(8).<br><br>Verdict: Evidence exists on the use of corticosteroid on croup focused on the initial dose. It does not suggest if an additional dose is effective. **The question has NOT been addressed in the evidence base.**	
20	What are the most effective and safe intravenous fluids to use on children and youth hospitalized in the general paediatric inpatient unit?	At what age is it acceptable to have kids NPO without having IV fluids running ?<br>Use of plasmalyte in DKA or more broadly in Pediatrics, Diabetes Guidelines regarding IV fluids .<br>what type of fluid and rate, in each age.<br>Are we monitoring patients with IV fluids for electrolytes imbalances.<br>Do IVs need to be TKVO or can they be saline locked (small study in London ON already)?<br>Utility of daily electrolytes for children on IV fluids.<br>improvement of being able to offer outpatient IV management of longer courses of antibiotics, say 7 days, at home instead of in hospital ,<br>we are starting to use Kitelok in our outpatients with long term central lines, I think this would be worth a multicentre randomized trial in inpatients re outcomes of line infection and occlusion even for shorter term in patient use. If you do this project then please reach out to me to participate as I would like to be collaborator.	CPS: Guidelines are present<br>Beck CE, Choong K, Puligandla PS, Hartfield D, Holland J, Lacroix J, Friedman JN. Avoiding hypotonic solutions in paediatrics: Keeping our patients safe. Paediatrics & child health. 2013 Feb 1;18(2):94-5.<br>Friedman JN, Canadian Paediatric Society, Acute Care Committee. Risk of acute hyponatremia in hospitalized children and youth receiving maintenance intravenous fluids. Paediatrics & child health. 2013 Feb 1;18(2):102-4.<br>AAP: Guidelines is present<br>Feld LG, Neuspiel DR, Foster BA, Leu MG, Garber MD, Austin K, Basu RK, Conway EE, Fehr JJ, Hawkins C, Kaplan RL. Clinical practice guideline: maintenance intravenous fluids in children. Pediatrics. 2018 Dec 1;142(6).<br>NICE: Guideline does recommend use of IV fluid but does not specify the type that is appropriate.<br>National Institute for Health and Care Excellence. Intravenous fluid therapy in children and young people in hospital. NG29. www.nice.org.uk/guidance/ng29<br><br>Foster, B. A., Tom, D., & Hill, V. (2014). Hypotonic versus isotonic fluids in hospitalized children: a systematic review and meta-analysis. The Journal of pediatrics, 165(1), 163-169.<br>Padua, A. P., Macaraya, J. R. G., Dans, L. F., & Anacleto, F. E. (2015). Isotonic versus hypotonic saline solution for maintenance intravenous fluid therapy in children: a systematic review. Pediatric nephrology, 30(7), 1163-1172.<br>Wang, J., Xu, E., & Xiao, Y. (2014). Isotonic versus hypotonic maintenance IV fluids in hospitalized children: a meta-analysis. Pediatrics, 133(1), 105-113.<br>McNab, S., Ware, R. S., Neville, K. A., Choong, K., Coulthard, M. G., Duke, T., ... & Dorofaeff, T. (2014). Isotonic versus hypotonic solutions for maintenance intravenous fluid administration in children. Cochrane Database of Systematic Reviews, (12).<br>Moritz ML, Ayus JC. Maintenance intravenous fluids in acutely ill patients. New England Journal of Medicine. 2015 Oct 1;373(14):1350-60.<br>Maisa Saddik, Salwa Farooqi. Systematic review and meta-analysis of low-chloride versus normal saline IV fluids in pediatric patients. PROSPERO 2017 CRD42017058633 Available from: https://www.crd.york.ac.uk/prospero/display_record.php?ID=CRD42017058633 [PROSPERO- in progress]<br><br>Verdict: In the ICU, hypotonic fluid leads to increased rates of hyponatremia compared to isotonic. For the GPIU, there is some evidence of similar increases in hyponatremia. Although there is no single IV fluid composition that is ideal for all children, an isotonic saline solution does appear to be the safer choice when maintenance IV fluid therapy is used in the general pediatric population.<br>Isotonic fluids are safer than hypotonic fluids in hospitalized children requiring maintenance IV fluid therapy in terms of plasma sodium levels.<br>Isotonic intravenous maintenance fluids with sodium concentrations similar to that of plasma reduce the risk of hyponatraemia when compared with hypotonic intravenous fluids. **The question has been partially addressed in the evidence base.**<br><br>Overall, the evidence suggests that isotonic fluid is safer than hypotonic fluid. The evidence gaps are mainly related to types of isotonic fluid compositions.	1 x nurse<br>8 x physician

© 2022 Gill PJ et al. *JAMA Network Open*.

21	What is the utility of routine blood cultures children and youth in hospitalized with common condition on a general paediatric inpatient unit?	The yield, and thus the usefulness of blood culture in different context, like urinary track infection, pneumonia, fever,,,,	CPS: No relevant guidelines.<br>AAP: No relevant guidelines.<br>NICE: No relevant guidelines.<br><br>Tam, P. Y. I., Bernstein, E., Ma, X., & Ferrieri, P. (2015). Blood culture in evaluation of pediatric community-acquired pneumonia: a systematic review and meta-analysis. Hospital pediatrics, 5(6), 324-336.<br><br>Verdict: Evidence on benefits of routine blood culture is limited. The only evidence found was focused on community acquire pneumonia (CAP). **The question has NOT been addressed in the evidence base.**	1 x physician
22	What is the most effective way to obtain and maintain IV access in children and youth hospitalized on the general paediatric inpatient unit?	Do patient/families prefer blood work/IV starts to be done in their rooms or in a separate treatment room?<br>Or why they didn't use NICU butterfly iv?<br>Why NICU nurses weren't putting the iv in my 4 week old?<br>What is the most appropriate IV maintenance fluid?<br>Which method to preserve IVs, saline lock or TKVO, is more effective?<br>Does IV TKVO improve PIV patency vs saline lock in children less than 2 years of age?	CPS: Guideline makes recommendations on positioning of child, but is limited overall.<br>Trottier ED, Doré-Bergeron MJ, Chauvin-Kimoff L, Baerg K, Ali S. Managing pain and distress in children undergoing brief diagnostic and therapeutic procedures. Paediatrics & child health. 2019 Dec 9;24(8):509-21.<br>AAP: No relevant guidelines.<br>NICE: No relevant guidelines.<br><br>Parker, S. I., Benzies, K. M., & Hayden, K. A. (2017). A systematic review: effectiveness of pediatric peripheral intravenous catheterization strategies. Journal of advanced nursing, 73(7), 1570-1582.<br>Qin, K. R., Ensor, N., Barnes, R., Englin, A., Nataraja, R. M., & Pacilli, M. (2020). Long peripheral catheters for intravenous access in adults and children: A systematic review of the literature. The Journal of Vascular Access, 1129729820927272.<br><br>Verdict: There was insufficient evidence to support the use of ultrasound, infrared light or transillumination. Interventions to reduce children's pain did not decrease first attempt success. **The question has NOT been addressed in the evidence base.**	3 x nurse<br>1 x physician<br>1 friend/family member
23	What are potential uses of point of care ultrasound in the general paediatric inpatient unit?	Potential uses of pocus in the pediatric inpatient setting<br>Applications of point of care ultrasound<br>introduction of POCUS ultrasound for IV insertion, PICC lines and LPs<br>Point of Care Ultrasound (POCUS) - training, national module.	CPS: No relevant guidelines.<br>AAP: Policy statement focused on pediatric emergency medicine but not GPIU care<br>American Academy of Pediatrics, Committee on Pediatric Emergency Medicine, American College of Emergency Physicians, Pediatric Emergency Medicine Committee. Point-of-care ultrasonography by pediatric emergency medicine physicians. Pediatrics. 2015 Apr 1;135(4):e1097-104.<br>NICE: No relevant guidelines.<br><br>Benabbas, R., Hanna, M., Shah, J., & Sinert, R. (2017). Diagnostic Accuracy of History, Physical Examination, Laboratory Tests, and Point-of-care Ultrasound for Pediatric Acute Appendicitis in the Emergency Department: A Systematic Review and Meta-analysis. Academic Emergency Medicine, 24(5), 523-551.<br>Hom, J., Kaplan, C., Fowler, S., Messina, C., Chandran, L., & Kunkov, S. (2020). Evidence-Based Diagnostic Test Accuracy of History, Physical Examination, and Imaging for Intussusception: A Systematic Review and Meta-analysis. Pediatric Emergency Care.<br>Lin-Martore, M., Kornblith, A. E., Kohn, M. A., & Gottlieb, M. (2020). Diagnostic Accuracy of Point-of-Care Ultrasound for Intussusception in Children Presenting to the Emergency Department: A Systematic Review and Meta-analysis. Western Journal of Emergency Medicine, 21(4), 1008.<br>MacMillan, B. (2018). Is point-of-care lung ultrasound more accurate than chest radiography for diagnosis of community acquired pneumonia in acutely dyspneic pediatric patients?. Canadian Journal of Respiratory Therapy, 54(2).<br>Simba, J., Sinha, I., Mburugu, P., Kaleem, M., Emadau, C., Harave, K., & Nabwera, H. (2020). Effect of point-of-care lung ultrasound on treatment outcomes in children: A systematic review. (DID NOT FIND)	4 x physician

© 2022 Gill PJ et al. *JAMA Network Open*.

			Wang, Y. H., Lee, C. C., Tsou, P. Y., & Ma, Y. K. (2019). Diagnostic accuracy of abdominal ultrasonography for intussusception in children: a systematic review and meta-analysis. (DID NOT FIND)<br><br>Verdict: A larger number of studiets and relevant evidence was identified which mostly focuses on POCUS use in the ED. There appears to be some evidence on the benefits of wide range of conditions but limited evidence focused on its use in the GPIU. **The question has NOT been ddressed in the evidence base.**	
24	What are effective strategies to mitigate the impacts of prolonged inpatient hospitalizations on general paediatric inpatient units?<br><br>e.g. addressing unmet needs, prolonged separation from family	Understanding the impact of prolonged hospitalization of Inuit children and youth from Nunavut on their families and especially on the caregivers. Isuues include: family separation due to the 1 escort policy, isolation, mental health challenges, emotional breakdown due to displacement and lack of support etc...).<br>What unmet needs do long term patients/families have<br>Does length of stay affect functional and rehabilitation outcomes?<br>How can healthcare providers best limit that negative impact or change hospitalization into a positive experience for children and their families?<br>What are the perceptions of patients and families of their inpatient experience after they have settled at home?<br>8) What are the experiences and needs of hospitalized families, especially those who experience multiple hospitalizations?	CPS: No relevant guideline.<br>AAP: No relevant guideline.<br>NICE: No relevant guideline.<br><br>Hahn, E., Seltzer, R., Wright-Sexton, L., Boss, R., & Henderson, C. (2017). The Intensive Care Unit Isn't Right, but There Is Nowhere Else to Go: Family Experience of Recurrent and Prolonged Pediatric Hospitalizations: A Systematic Review (S766). Journal of Pain and Symptom Management, 53(2), 446-447.<br><br>Verdict: Only one systematic review was identified, but it is the abstract only and focused on care based in the ICU. **The question has NOT been addressed in the evidence base.**	3 x physician<br>1 x speech language pathologist<br>1 x nurse
25	What warning systems/monitoring tools are effective at improving outcomes for hospitalized children and youth on a general paediatric inpatient unit?	Standardization of Paediatric Early Warning Systems (PEWS) across the country innovative models to move patients into the community and shorten stays and expediate discharges<br>b) Best practices for early recognition of the deteriorating child<br>How often children under specialized services (ENT, ortho,) require SPOT team assistance for management as compared to patients under gen peds (ratios).<br>Overuse of the SPOT team (CCRT) for basics that should be covered with Nursing educator and lack of accountability of MRP when recommendations are made.<br>Level of acuity of patients kept on the wards when they need PICU or NICU and there are no available beds.	CPS: No relevant guideline.<br>AAP: No relevant guideline.<br>NICE: No relevant guideline.<br><br>Lambert, V., Matthews, A., MacDonell, R., & Fitzsimons, J. (2017). Paediatric early warning systems for detecting and responding to clinical deterioration in children: a systematic review. BMJ open, 7(3).<br>Le Lagadec, M. D., & Dwyer, T. (2017). Scoping review: the use of early warning systems for the identification of in-hospital patients at risk of deterioration. Australian Critical Care, 30(4), 211-218.<br>McNeill, G., & Bryden, D. (2013). Do either early warning systems or emergency response teams improve hospital patient survival? A systematic review. Resuscitation, 84(12), 1652-1667.<br>Thomas-Jones, E., Lloyd, A., Roland, D., Sefton, G., Tume, L., Hood, K., ... & Powell, C. (2018). A prospective, mixed-methods, before and after study to identify the evidence base for the core components of an effective Paediatric Early Warning System and the development of an implementation package containing those core recommendations for use in the UK: Paediatric early warning system–utilisation and mortality avoidance–the PUMA study protocol. BMC pediatrics, 18(1), 1-13.<br>Trubey, R., Huang, C., Lugg-Widger, F. V., Hood, K., Allen, D., Edwards, D., ... & Powell, C. (2019). Validity and effectiveness of paediatric early warning systems and track and trigger tools for identifying and reducing clinical deterioration in hospitalised children: a systematic review. BMJ open, 9(5), e022105.<br>Department of Health (2016, V2). The Irish Paediatric Early Warning System (PEWS) (NCEC National Clinical Guideline No. 12). Available at: http://health.gov.ie/national-patient-safety-office/ncec/<br>Kerenza Hood,.et al. Review of Paediatric Early Warning Systems (PEWS) and scores for clinical deterioration of children in hospital: their development and validation, effectiveness and factors associated with implementation and generative mechanisms.. PROSPERO 2015 CRD42015015326 Available from: https://www.crd.york.ac.uk/prospero/display_record.php?ID=CRD42015015326 [in progress]	2 x respiratory therapist<br>1 x nurse<br>1 x patient safety research/web content editor

© 2022 Gill PJ et al. *JAMA Network Open*.

			Verdict: There are wide range of warning systems and monitoring tools in the published literature. There is some evidences but at present, there are no systematic reviews which identify which ones are effective. **The question has been partially addressed in the evidence base.**<br>[Findings revealed that PEWS are extensively used internationally in paediatric inpatient hospital settings. However, robust empirical evidence on which PEWS is most effective was limited. The studies examined did however highlight some evidence of positive directional trends in improving clinical and process-based outcomes for clinically deteriorating children<br>There is low level quantitative evidence that EWS improve patient outcomes and strong anecdotal evidence that they augment the ability of the clinical staff to recognise and respond to patient decline, thus reducing the incidence of severe adverse events.]<br>It is unclear as to what specific tools.	
26	What are effective interventions for children and youth hospitalized with cyclic vomiting on the general paediatric inpatient unit?	Severe, refractory cyclic vomiting syndrome: establish a national network/database to help inform care decisions for this challenging patient population	CPS: No guideline found<br>AAP: No guideline found<br>NICE: No guideline found<br><br>Gui, S., Patel, N., Issenman, R., & Kam, A. J. (2019). Acute management of pediatric cyclic vomiting syndrome: a systematic review. The Journal of pediatrics, 214, 158-164.<br>Lee, L. Y., Abbott, L., Mahlangu, B., Moodie, S. J., & Anderson, S. (2012). The management of cyclic vomiting syndrome: a systematic review. European journal of gastroenterology & hepatology, 24(9), 1001-1006.<br><br>Verdict: Studies found are based on findings in acute care setting or ED. While ondansetron is an effective pharmacotherapy, overall, t**he question has NOT been addressed in the evidence base**	1 x physician
27	What are the most effective interventions in infants, children and youth with gastroesophageal reflux disease (GERD) on the general peadiatric inpatient unit?	Typical practices for the assessment of swallowing and GERD as well as management of GERD and 'silent aspiration' in ex preterm neonates<br>Indication to repeat the dose, etc.) / Assessment and treatment of reflux	CPS: No guideline found<br>AAP: Practice guideline identified but the evidence is unclear.<br>Randel A. AAP releases guideline for the management of gastroesophageal reflux in children. American family physician. 2014 Mar 1;89(5):395-7.<br>Lightdale JR, Gremse DA. Gastroesophageal reflux: management guidance for the pediatrician. Pediatrics. 2013 May 1;131(5):e1684-95.<br>NICE: Guideline available<br>Gastro-oesophageal reflux disease in children and young people: diagnosis and management [https://www.nice.org.uk/guidance/ng1/chapter/1-Recommendations#enteral-tube-feeding-for-gord]<br><br>Jancelewicz, T., Lopez, M. E., Downard, C. D., Islam, S., Baird, R., Rangel, S. J., ... & Goldin, A. B. (2017). Surgical management of gastroesophageal reflux disease (GERD) in children: A systematic review. Journal of pediatric surgery, 52(8), 1228-1238. [ARTICLE NOT FOUND]<br>Mauritz, F. A., van Herwaarden-Lindeboom, M. Y., Stomp, W., Zwaveling, S., Fischer, K., Houwen, R. H., ... & van der Zee, D. C. (2011). The effects and efficacy of antireflux surgery in children with gastroesophageal reflux disease: a systematic review. Journal of Gastrointestinal Surgery, 15(10), 1872-1878.<br>Zhang, P., Tian, J., Jing, L., Wang, Q., Tian, J., & Lun, L. (2016). Laparoscopic vs. open Nissen's fundoplication for gastro-oesophageal reflux disease in children: a meta-analysis. International Journal of Surgery, 34, 10-16.<br><br>Verdict: Three studies identified but they focus on surgical interventions only. **The question has NOT been addressed in the evidence base**	2 x physician

© 2022 Gill PJ et al. *JAMA Network Open*.

28	What is the most effective way to standardize care and reduce unnecessary variation in care delivered to hospitalized children and youth on the general paediatric inpatient unit?	major differences in approach by specialists (some anxious to operate, others need to be encouraged to act);<br>Too much variability by practitioners.<br>Poor clinical evidence informed practice<br>I would like to see research geared to quality improvements on how to increase standardization to provide more consistent care across pediatric units and hospitals/programs and an improvement to the accessibility of up to date resources when nurses need them to support optimal and safe practice for nurses and all healthcare workers when providing care to pediatric patients.<br>Sharing order sets for common pediatric conditions (i.e. Kawasaki Disease, Sickle Cell, Sepsis, etc).<br>Which decisions made by paediatricians are evidenced-based and which are not?<br>Is consistent evidence-based care provided ? That is irregardless of who is "on" does a child with a defined clinical problem set receive the same care I fully expect there to be differences, but do these differences in care provide a 'natural' experiment to compare outcomes and tranlaste practices in centres with better outcomes to those with less robust outcomes.<br>Increased evidence in pediatrics surrounding common presentations	CPS: No guideline found<br>AAP: No guideline found<br>NICE: No guideline found<br><br>Lion KC, Wright DR, Spencer S, Zhou C, Del Beccaro M, Mangione-Smith R. Standardized clinical pathways for hospitalized children and outcomes. Pediatrics. 2016 Apr 1;137(4).<br><br>Verdict: The only article found reports that standardized care reduces cost, but does not specify the most effective way. **The question has NOT been addressed in the evidence base.**	1 x friend/family member<br>5 x physician<br>2 x nurse
29	What is the most effective treatment strategy for hospitalized children and youth with neurologic impairment who suffer from chronic respiratory illness on the general paediatric inpatient unit?	What are the best management strategies for respiratory symptoms / sialorrhea in children with neurological impairment?<br>Does the use of secretion thickening agents (ex. atropine) help with airway clearance OR the question could be what outcome measures should be used to determine the effectiveness of the use of secretions thickening agents<br>Use of Dornase/pulmozyme for patients with hypotonia to help with respiratory secretion clearance	CPS: No guideline found<br>AAP: No guideline found<br>NICE: No guideline found<br><br>No studies identified<br><br>Verdict: No studies identified. **The question has NOT been addressed in the evidence base.**	1 x physician<br>1 x physical therapist
30	How can we ensure that healthcare delivery in hospital meets the needs of children and youth with developmental disabilities on the general paediatric inpatient unit?<br><br>(or is there evidence that healthcare delivery needs to be different, unique for this population - needs presumes evidence)	My daughter had a rare genetic condition at birth. During her 57-day stay at the paediatric hospital, she was seen by many doctors and received many types of tests, the medical team was not able to give a precise medical diagnosis, let alone an effective treatment plan. For us (parents), everyday our question was - what would be the effective treatment plan for our daughter?<br>My largest concern is around the assumptions that healthcare workers make with non-neurotypical children. I feel that these incorrect assumptions have impacted the quality of my child's care.<br>Our other concern is our child is diagnosed with ASD. When he was younger getting him to take the inhalers while it was still manageable a challenge and I found not everyone was equipped with the knowledge to help with those challenges.	CPS: Guideline found does not clarify the uncertainity<br>AAP: No guidelines were identified.<br>NICE: Guideline found on Autism Spectrum disorder (ASD) and conduct disorder but does not specify the details on care in GPIU.<br>National Institute for Health & Care Excellence. Autism spectrum disorder in under 19s: support and management. NICE guidelines [CG170], August. Available at: https://www. nice. org. uk/guidance/cg170. 2013 Aug.<br><br>Belanger, R., Leroux, D., & Lefebvre, P. (2021). Supporting caregivers of children born prematurely in the development of language: A scoping review. Paediatrics & Child Health, 26(1), e17-e24.<br>Fang, Z., Barlow, J., & Zhang, C. (2020). Parenting programs that address physical abuse in childhood for families of children with developmental disabilities in mainland China: systematic review and meta-regression. Trauma, Violence, & Abuse, 1524838020915599.<br><br>Verdict: Studies identified do not address uncertainty. **The question has NOT been addressed in the evidence base.**	3 x friend/family member
31	What are effective support strategies for parents, families and children/youth	Support strategies for parents with a child with a recent acquired disability<br>No where to sleep.<br>interested in more research about day/night cycles and the impact	CPS: No guidelines were found<br>AAP: No guidelines were found<br>NICE: No guidelines were found	1 x recreation therapist<br>3 x parent/caregiver<br>1 x nurse<br>5 x physician

	hospitalized on the general paediatric inpatient unit?<br><br>E.g. support groups, private rooms/sleeping arrangements, breastfeeding support, physical activity, making the ward more adolescent-friendly, screen time, Indigenous communities and spiritual care	of disturbing these in inpatient settings.<br>Parent education/ comfort in caring for children is a huge area requiring more study .<br>the impact of the parent-partner to reduce the length of hospitalization recommendations for discharge and rehospitalization in the following mont<br>How can we make hospitals a more adolescent-friendly place?<br>Teens admitted in hospital going through mental health problems in hospital they don't necessarily want to discuss with parents there.<br>What is their satisfaction<br>why is it so expensive to be an inpatient? (parking, food, etc.)<br>allowing parents to help prepare their children for tests, including being present when they are given anesthetic.<br>other psychosocial aspects related to patient care and patient outcomes, such as illness adjustment, treatment adherence, parent-child relationship, parent or child mental health<br>3. What is the optimal clothing for hospitalization? Hospital gowns are uncomfortable; I usually wore pajamas but those also make ports difficult to access.<br>what are innovative models of nursing care to support this population especially considering ages and requirements for supportive care when care often is supported by parents<br>4) How much does the time of day and the day of week of an in-patient admission affect the extent and quality of the orientation? (In our experience, it seemed to have a big effect.)<br>Parent education/ comfort in caring for children is a huge area requiring more study - what works, how to support digitally<br>is there any way to prioritize sleep for in-patients.<br>What's the best strategy for staying somewhat active when admitted?<br>Determining the positive health benefit of providing psychosocial intervention to parents/caregivers during their child's hospitalization .<br>Effect size of psychosocial intervention<br>Improving CHEO's modelling of back to sleep guidelines (we currently have infants consistently positioned with head of bed raised, swaddled, multiple items in the cribs, orders for swaddling infants when this is contrary to current well baby guidelines, and babies still on monitors when discharge is imminent. Not sure what the research question needs to be, but infants should be sleeping consistently on their backs, head of bed flat, and nothing in the bed, not swaddled for the whole of their stay, unless medically indicated otherwise and then discharge orders need to be clear when advice for safe sleep at discharge is different than what is currently recommended for the general public.<br>Are the children living productive and healthy lives inside the ward, with contact to multiple outside people to ensure good social lives?	Brett, J., Staniszewska, S., Newburn, M., Jones, N., & Taylor, L. (2011).. BMJ open, 1(1).<br>Grimshaw, S. L., Taylor, N. F., & Shields, N. (2016). The feasibility of physical activity interventions during the intense treatment phase for children and adolescents with cancer: a systematic review. Pediatric blood & cancer, 63(9), 1586-1593.<br>do Lago, A. S. D., Zaffarani, C., Mendonça, J. F. B., Moran, C. A., Costa, D., & Gomes, E. L. D. F. D. (2020). Effects of physical exercise for children and adolescents undergoing hematopoietic stem cell transplantation: a systematic review and meta-analysis. Hematology, transfusion and cell therapy.<br>Rustler, V., Hagerty, M., Daeggelmann, J., Marjerrison, S., Bloch, W., & Baumann, F. T. (2017). Exercise interventions for patients with pediatric cancer during inpatient acute care: a systematic review of literature. Pediatric blood & cancer, 64(11), e26567.<br>Sperle, C. (2019, October). SEIZE THE DAY! EXERCISE FOR THE HOSPITALIZED CF PATIENT. In PEDIATRIC PULMONOLOGY (Vol. 54, pp. S144-S145). 111 RIVER ST, HOBOKEN 07030-5774, NJ USA: WILEY. [NOT FOUND]<br>Sridharan, K., & Sivaramakrishnan, G. (2017). Therapeutic clowns in pediatrics: a systematic review and meta-analysis of randomized controlled trials-corrigendum. European journal of pediatrics, 176(2), 289-289.<br><br>Verdict: Some studies have been conducted in this area, for example on physical exercise and therapeutic clown on certain groups of pediatric patients. However, the overall evidence-base is uncertain for this query. **The question has NOT been addressed in the evidence base**	3 x social worker<br>2 x youth<br>1 x graduate student
32	Can the nutritional needs of children/youth and their families be addressed as an essential part of the	Not provided food.<br>Assessment of nutritional status.<br>what are the benefits of a nutrition screening tool upon .<br>Food is not appropriate to the MEDICAL COMPLAINT that the youth is admitted for !!<br>what are the efficient tools to standardize length measurements,	CPS: No guidelines were found<br>AAP: No guidelines were found<br>NICE: No guidelines were found<br><br>Critch, J. N., Society, C. P., & Nutrition and Gastroenterology Committee. (2014). Nutrition for healthy term infants, six to 24 months: An overview. Paediatrics &	2 x parent/caregiver<br>4 x physician<br>2 x dietician<br>2 x nurse<br>3 x friend/family member

© 2022 Gill PJ et al. *JAMA Network Open*.

	therapeutic care plan on a general paediatric inpatient unit and what are effective methods to support children and families's nuritional needs?	Is there better ways we can assess pt's that have issues with feeding tolerance and can we create some sort of pathway for having MDs support nurses in addressing/supporting parents who are ++ hesitant to advance feeds. Seems to be a repeat issue leading to ++long admissions on 7d.<br>How well do we support the recommendations of good health with admitted patients - ie electronic time, nutrition, physical activitiy, etc. We should not just ask about medications but care and nutrition that is specific to the care of the child.<br>We should not just ask about medications but care and nutrition that is specific to the care of the child.<br>Mothers who are breastfeeding their child in hospital have 0 support.<br>Are there any specific coping mechanisms that healthcare professionals can help chronically ill children/families develop?<br>What are interventions to reduce food insecurity for families during the inpatient stay ?<br>Food choice and support for those who are experiencing food insecurity as a family during the stay ?<br>Food was such a high issue we have to request 4 times to only send chicken or vegetables and finally I told them to not to get food we will get it from outside or I will leave my daughter in hospital to go home to cook. I did not like wasting so much food someone else could have eaten it. Not very good menu. Food is not appropriate to the MEDICAL COMPLAINT that the youth is admitted for !! Not provided food.<br>Also, I should be allowed to get a coffee when I have not eaten for 24 hours.	child health, 19(10), 547.<br>Patro-Gołąb, B., Shamir, R., & Szajewska, H. (2015). Yogurt for treating acute gastroenteritis in children: systematic review and meta-analysis. Clinical Nutrition, 34(5), 818-824.<br><br>Verdict: Some studies were identified but many aspects of the questions remain unanswered. **The question has NOT been addressed in the evidence base**	
33	When it is appropriate to involve allied health care professionals (e.g. OT, PT, child life specialists) in the care of hospitalized children/youth on the general paediatric inpatient unit?	increase in the number of resources available for patients such as child life specialists and OT/PT support.<br>Access to Child Therapist during hospitalization and family overall experience on the general paediatric ward.<br>Are there any specific coping mechanisms that healthcare professionals can help chronically ill children/families develop?<br>BC Children's Hospital does not have a Speech-Language Pathologist for it's inpatient patients, is this affecting that outcomes or patient stay?<br>Should acute care patients have access to Speech Language Pathologist?<br>Respiratory ward : Respiratory Physiotherapy should be a very major part of this ward which is NO WHERE TO BE SEEN !! it's a real disgrace<br>example can we reduce the workload of the doctor by giving more latitude to the respiratory therapist?<br>Poor RT resource availability on wards that can improve patient outcomes better than NP and PA …<br>we are the experts in airway and ventilation ...especially in chronic care of trach ventilated patients. unbalance use of RT resources that can improve patient outcomes and D/C dates	CPS: No guidelines were found<br>AAP: No guidelines were found<br>NICE: No guidelines were found<br><br>Bryant-Lukosius, D., Carter, N., Reid, K., Donald, F., Martin-Misener, R., Kilpatrick, K., ... & DiCenso, A. (2015). The clinical effectiveness and cost-effectiveness of clinical nurse specialist-led hospital to home transitional care: a systematic review. Journal of evaluation in clinical practice, 21(5), 763-781.<br>Taylor, J., Booth, A., Beresford, B., Phillips, B., Wright, K., & Fraser, L. (2020). Specialist paediatric palliative care for children and young people with cancer: A mixed-methods systematic review. Palliative medicine, 34(6), 731-775.<br><br>Verdict: The only systematic review includes mainly adult studies, with only 3 on infants, and 1 in the NICU. There is very little pediatric specific evidence. **The question has NOT been addressed in the evidence base**	2 x nurse<br>2 x physician<br>2 x speech language pathologist<br>1 x friend/family member<br>2 x respiratory therapist
34	What are best practices and support strategies for Indigenous parents, families and children/youth on the	Understanding the impact of prolonged hospitalization of Inuit children and youth from Nunavut on their families and especially on the caregivers. Isuues include: family separation due to the 1 escort policy, isolation, mental health challenges, emotional breakdown due to displacement and lack of support etc...).<br>What is the impact of medical transport to a pediatric center on	CPS: There are relevant guidelines but not focused on GPIU care.<br>Giroux R, Blackstock C, Jetty R, Bennett S, Gander S. COVID-19 and Indigenous children in Canada: what can paediatricans do. CPS Blog. 2020 May 27.<br>Jetty R. Tuberculosis among First Nations, Inuit and Métis children and youth in Canada: Beyond medical management. Paediatrics & Child Health. 2021 Apr;26(2):e78-81.	3 x physician<br>1 x nurse

© 2022 Gill PJ et al. *JAMA Network Open*.

	general paediatric inpatient unit?	children youth and families from Nuanvut. What is the average length of stay. What are the factors that impact the longer length of stay?<br>How we can better take care of our indigenous patients/families, barriers to giving great care to this population.<br>What are the experiences of Inuit children, youth and their families from Nunavut who require hospitalization at CHEO.<br>What are the factors that cause a medically complex child to be placed into foster care instead returning back home to Nunavut?	AAP: No guidelines were found<br>NICE: No guidelines were found<br><br>He H, Xiao L, Torrie JE, Auger N, McHugh NG, Zoungrana H, Luo ZC. Disparities in infant hospitalizations in Indigenous and non-Indigenous populations in Quebec, Canada. CMAJ. 2017 May 29;189(21):E739-46.<br>Dossetor PJ, Martiniuk AL, Fitzpatrick JP, Oscar J, Carter M, Watkins R, Elliott EJ, Jeffery HE, Harley D. Pediatric hospital admissions in Indigenous children: a population-based study in remote Australia. BMC pediatrics. 2017 Dec;17(1):1-3.<br>Fuentes MM, Jimenez N, Apkon SD, Rivara FP. Functional outcomes during inpatient rehabilitation for American Indian and Alaska Native children with traumatic brain injury. Journal of pediatric rehabilitation medicine. 2016 Jan 1;9(2):133-41.<br><br>Verdict: Some of the identified articles studied interventions directed to indigenous children, but they were not systematic reviews and were not relevant to GPIU care.<br>**The question has NOT been addressed in the evidence base**	
35	What are ways to support breastfeeding mothers when their breastfed infant is hospitalized on the general paediatric inpatient unit?	1. What % of babies, admitted for diagnoses of failure to thrive or cow's milk protein allergy and admitted on breastfeeding or EBM are still receiving breast milk 3 months after discharge and what can we do to improve this rate<br>Mothers who are breastfeeding their child in hospital have 0 support.<br>Breastfeeding friendly hospitals- babies admitted with dehydration/needing top up since moms milk isn't in. Moms are not encouraged to use formula on post partum unit (in some cases the moms don't even want to breastfeed).<br>What are the barriers to breastfeeding mothers being supported when their breastfed infant is hospitalized?<br>Admission could have possibly been avoided had more education been provided to parents regarding intake/output/breastfeeding and topping up	CPS: The Baby-Friendly Hospital Initiative (BFHI)<br>Pound CM, Unger SL, Canadian Paediatric Society, Nutrition and Gastroenterology Committee, Hospital Paediatrics Section. The baby-friendly initiative: Protecting, promoting and supporting breastfeeding. Paediatrics & child health. 2012 Jun 1;17(6):317-21.<br>AAP: No guidelines were found<br>NICE: No guidelines were found<br><br>Beake S, Pellowe C, Dykes F, Schmied V, Bick D. A systematic review of structured versus non-structured breastfeeding programmes to support the initiation and duration of exclusive breastfeeding in acute and primary healthcare settings. JBI Evidence Synthesis. 2011 Jan 1;9(36):1471-508.<br>Alves E, Rodrigues C, Fraga S, Barros H, Silva S. Parents' views on factors that help or hinder breast milk supply in neonatal care units: systematic review. Archives of Disease in Childhood-Fetal and Neonatal Edition. 2013 Nov 1;98(6):F511-7.<br>Balogun OO, Dagvadorj A, Yourkavitch J, da Silva Lopes K, Suto M, Takemoto Y, Mori R, Rayco-Solon P, Ota E. Health facility staff training for improving breastfeeding outcome: A systematic review for step 2 of the Baby-Friendly Hospital initiative. Breastfeeding Medicine. 2017 Nov 1;12(9):537-46.<br>Pérez-Escamilla R, Martinez JL, Segura-Pérez S. Impact of the Baby-friendly Hospital Initiative on breastfeeding and child health outcomes: a systematic review. Maternal & child nutrition. 2016 Jul;12(3):402-17.<br>Moran VH, Morgan H, Rothnie K, MacLennan G, Stewart F, Thomson G, Crossland N, Tappin D, Campbell M, Hoddinott P. Incentives to promote breastfeeding: a systematic review. Pediatrics. 2015 Mar 1;135(3):e687-702.<br>McKinney CM, Glass RP, Coffey P, Rue T, Vaughn MG, Cunningham M. Feeding neonates by cup: A systematic review of the literature. Maternal and child health journal. 2016 Aug;20(8):1620-33.<br>Carroll G, Safon C, Buccini G, Vilar-Compte M, Teruel G, Pérez-Escamilla R. A systematic review of costing studies for implementing and scaling-up breastfeeding interventions: what do we know and what are the gaps?. Health policy and planning. 2020 May;35(4):461-501.<br>Yi CM, Man HS. The effectiveness of breastfeeding education on maternal breastfeeding self-efficacy and breastfeeding duration: a systematic review. The JBI Database of Systematic Reviews and Implementation Reports. 2011;9(64 Suppl):261-84.<br>Chepkirui D, Nzinga J, Jemutai J, Tsofa B, Jones C, Mwangome M. A scoping review of breastfeeding peer support models applied in hospital settings.	1 x dietician

© 2022 Gill PJ et al. *JAMA Network Open*.

			International Breastfeeding Journal. 2020 Dec;15(1):1-1.<br>Donovan TJ, Buchanan K. Medications for increasing milk supply in mothers expressing breastmilk for their preterm hospitalised infants. Cochrane Database of Systematic Reviews. 2012(3).<br>Gavine A, MacGillivray S, Renfrew MJ, Siebelt L, Haggi H, McFadden A. Education and training of healthcare staff in the knowledge, attitudes and skills needed to work effectively with breastfeeding women: a systematic review. International breastfeeding journal. 2016 Dec;12(1):1-0.<br><br>Verdict: There were several systematic reviews identified that guided initiation and continuation of breast feeding, but none of them were directed towards supporting mothers in the GPIU. Breast feeding peer support groups were also studies, but the outcome was based on health status of infant and not directly related to mother. **The question has NOT been addressed in the evidence base**	
36	What are effective ways to incorporate patients and families unique values, goals, and socio-demographic factors to optimize care on the general paediatric inpatient unit?	Part of equity, diversity, and inclusivity must be respect for individual and their health related values, goals, and preferences, as these reflect sociocultural norms for the individual.<br>I would like to see health care professionals have a better understanding of the social factors that contribute to illness in children. If we can improve these social problems, we can improve their physical and mental health.<br>Understanding the impact of prolonged hospitalization of Inuit children and youth from Nunavut on their families and especially on the caregivers. Isuues include: family separation due to the 1 escort policy, isolation, mental health challenges, emotional breakdown due to displacement and lack of support etc...).<br>7) How can we better address social determinants of health and the health inequities that affect hospitalized children (pre-during-post-hospitalization)	CPS: No guidelines were found<br>AAP: No guidelines were found<br>NICE: No guidelines were found<br><br>No studies identified<br><br>Verdict: No studies identified. **The question has NOT been addressed in the evidence base**	3 x physician
37	What is the impact of the patient's room/environment on health outcomes on the general paediatric inpatient unit?<br><br>E.g. noise, lights, private/shared room, window/no window	What is the evidence for environmental interventions to reduce stress for families and staff?<br>The benefits of private rooms vs shared rooms<br>Window bed vs no window bed - effects on recovery and mental health<br>Appropriate assignment of roommates in shared rooms<br>Security for patients and staff with potentially violent patients<br>Cleanliness and disinfecting rooms at the time of patient turn over<br>The effect of noise/lights in a general pediatric ward on the healing and wellbeing of children admitted to the ward<br>Effect of noise/lights at night on the pediatric ward for children admitted to the ward<br>could more space in hospital rooms to allow for mobility (such as dedicated playmat for time out of cribs for infants) decrease length of stay?	CPS: No guidelines were found<br>AAP: No guidelines were found<br>NICE: No guidelines were found<br><br>Ng, C. A., Ho, J. J., & Lee, Z. H. (2019). The effect of rooming-in on duration of breastfeeding: A systematic review of randomised and non-randomised prospective controlled studies. PloS one, 14(4), e0215869.<br>Norton-Westwood, D., Pearson, A., & Robertson-Malt, S. (2011). The ability of environmental healthcare design strategies to impact event related anxiety in paediatric patients: A comprehensive systematic review. JBI Evidence Synthesis, 9(44), 1828-1882.<br>van Veenendaal, N. R., Heideman, W. H., Limpens, J., van der Lee, J. H., van Goudoever, J. B., van Kempen, A. A., & van der Schoor, S. R. (2019). Hospitalising preterm infants in single family rooms versus open bay units: a systematic review and meta-analysis. The Lancet Child & Adolescent Health, 3(3), 147-157.<br>van Veenendaal, N. R., van Kempen, A. A., Franck, L. S., O'Brien, K., Limpens, J., van der Lee, J. H., ... & van der Schoor, S. R. (2020). Hospitalising preterm infants in single family rooms versus open bay units: A systematic review and meta-analysis of impact on parents. EClinicalMedicine, 23, 100388. [PARENTS]<br><br>Verdict: Norton-westwood et al. study does identify the impact of environment on outcomes. However, most of the studies identified were qualitative in nature, and the only outcome studied was anxiety in children. The review did cover all aspects like noise, light, shared room, presence of window, etc. Overall, given the small number of patients available, there are several areas that warrant future research. **The question has been partially addressed in the evidence base**	3 x physician<br>1 x friend/family member<br>1 x physical therapist

© 2022 Gill PJ et al. *JAMA Network Open*.

38	What are ways to structure multiple clinical assessments and consultations to minimize discomfort and reduce unnecessary tests or treatments on children hospitalized on the general paediatric inpatient unit ?	Sometimes I recognize that many visits were necessary because my child was acutely sick. But sometimes a resident or other would come, wake my son, in the middle of the night, 20 minutes after the last exam, done by someone else. .<br>Is there a way to put these exams together so that once kids fall asleep, they are able to get some quality sleep.<br>Unnecessary investigations for certain patients<br>Unnecessary monitoring of patients rather than clinical observations.<br>Unnecessary admissions, tests and treatments ,<br>Overuse of X-Ray in Bronchiolitis and Asthma<br>can we reduce orders for vital signs In stable patients with a small education intervention or order set?<br>Looking at how often investigations are actually changing management<br>When an innocent child strays onto the hospital ward they are at risk for many interventions...many possibly most non evidence based the longer a child is an inpatient the greater odd of being jabbed with a sharp implement to expensively generate a number that will have no bearing on anything or... her or his thorax irradiated for chest Xray with zero impact So, I'd love a list of top ten non evidence-based things we do all the time that we don't need to strict ins and outs, vitals q4h, electrolytes, CBC because a child is hospitalized doesn't mean we have to..get to assault them with our technology plus, many in patient can probably go home 12-36 hrs sooner than they do now with no increased risk people are driven by theoretical catastrophes that are in the vast majority of cases, non existent so, a hard look at what we do in house..and doing nothing perfectly acceptable...and impact of discharging earlier. have been giving my cell number to parents as they head out the door for as long as I've had one and 0 readmits...clearly not discharging soon enough!	No relevant guidelines	1 x friend/family member<br>6 x physician
39	What are effective ways to incorporate shared decision-making with parents and children/youth hospitalized on the general paediatric inpatient unit?<br><br>"Effectiveness" defined as length of stay, caregiver confidence	Attention needs to be paid to treatment options that families want explored, whether conventional, complementary, or traditional in origin.<br>multiD handover and rounds how it is run and the best way to run to incorp family centered care.<br>5) Do rounds that genuinely include parents shorten stays, increase the knowledge and confidence of parents at discharge, or help families participate more competently and confidently in the child's care?<br>Involving children and youth in decision making about their health and treatment,<br>Parental involvement with care while in hospital effects on outcomes.<br>4) How can we better partner with families/caregivers for optimal and effective shared decision making .<br>How can parents and caregivers be better engaged in the process of discharge?<br>How much did patients understand about their hospitalization/discharge?	CPS: A position statment is present which provides some guidance, but it is not specific to GPIU.<br>Coughlin KW. Medical decision-making in paediatrics: infancy to adolescence. Paediatrics & child health. 2018 Apr 12;23(2):138-46.<br>AAP: Few articles were found, but no guidelines.<br>Opel DJ. A 4-step framework for shared decision-making in pediatrics. Pediatrics. 2018 Nov 1;142(Supplement 3):S149-56.<br>Adams RC, Levy SE. Shared decision-making and children with disabilities: pathways to consensus. Pediatrics. 2017 Jun 1;139(6).<br>NICE: In process of developing a shared decision making guideline (age category: unspecified; tentative publication 17 June 2021)<br><br>Malone, H., Biggar, S., Javadpour, S., Edworthy, Z., Sheaf, G., & Coyne, I. (2019). Interventions for promoting participation in shared decision-making for children and adolescents with cystic fibrosis. The Cochrane database of systematic reviews, 5(5), CD012578. https://doi.org/10.1002/14651858.CD012578.pub2<br>Coyne, I., O'Mathúna, D. P., Gibson, F., Shields, L., Leclercq, E., & Sheaf, G. (2016). Interventions for promoting participation in shared decision-making for children with cancer. The Cochrane database of systematic reviews, 11(11), CD008970. https://doi.org/10.1002/14651858.CD008970.pub3<br>Kew, K. M., Malik, P., Aniruddhan, K., & Normansell, R. (2017). Shared decision-making for people with asthma. Cochrane Database of Systematic Reviews, (10).<br>Liverpool, S., Pereira, B., Hayes, D., Wolpert, M., & Edbrooke-Childs, J. (2020). A	3 x physician<br>2 x parent/caregiver<br>2 x nurse

© 2022 Gill PJ et al. *JAMA Network Open*.

			scoping review and assessment of essential elements of shared decision-making of parent-involved interventions in child and adolescent mental health. European child & adolescent psychiatry, 1-20.<br>Wyatt, K. D., List, B., Brinkman, W. B., Lopez, G. P., Asi, N., Erwin, P., ... & LeBlanc, A. (2015). Shared decision making in pediatrics: a systematic review and meta-analysis. Academic pediatrics, 15(6), 573-583.<br>Yamaji, N., Suto, M., Takemoto, Y., Suzuki, D., Lopes, K. D. S., & Ota, E. (2020). Supporting the decision making of children with cancer: a meta-synthesis. Journal of Pediatric Oncology Nursing, 37(6), 431-443.<br><br>Verdict: Most of the research identified focuses on specific populations, and not on those on GPIU. The one systematic review by Wyatt et al outlines that there is an impact on parents (but no data on outcomes) and few if any on children. Overall, the **question has been partially addressed in the evidence base**	
40	What is the most effective way to conduct medical rounds, including how to involve caregivers and patients in the decision making while on the general paediatric inpatient unit?	how to evaluate if bedside rounds are actually working for patients/ nurses/ learner<br>multiD handover and rounds how it is run and the best way to run to incorp family centered care<br>Why do most centres claim to offer "family centred care" but spend most of their time speaking for and about patients on rounds?	CPS: No guideline identified.<br>AAP: No guideline identified.<br>NICE: No guideline identified.<br><br>Ratelle, J. T., Sawatsky, A. P., Kashiwagi, D. T., Schouten, W. M., Erwin, P. J., Gonzalo, J. D., ... & West, C. P. (2019). Implementing bedside rounds to improve patient-centred outcomes: a systematic review. BMJ quality & safety, 28(4), 317-326.<br><br>Verdict: Available evidence does not address the specific question. **The question has NOT been addressed in the evidence base.**	3 x physician
41	How does the integration of virtual care on the general paediatric inpatient unit help to limit communication barriers affecting the outcome in patient and family experience?	looking towards virtual and innovative ways to provide this<br>I would love to know the 1) cost-effectiveness of the increased shift to virtual health<br>2) the impact on children and their families of conducting healthcare in this manner.<br>esp how to even further safely reduce LOS (least amount of treatment, supporting patients with FU clinics/ virtual care.<br>Tertiary- regional connections for inpatient care- virtual supports to keep kids in regional sites rather than use tertiary beds that are in short supply- expanding the reach of tertiary resources using Virtual health (evaluating a program for this now but it has been very challanging to develop) communication / barriers to communication	CPS: Guidance specific to telehealth in physician's office.<br>Sharon R. Providing virtual care during a pandemic: a guide to telemedicine in the paediatric office [blog]. Ottawa: Canadian Paediatric Society. 2020 Mar 26.<br>AAP: Guidance related to the COVID-19 pandemic<br>American Academy of Pediatrics. Guidance on the Necessary Use of Telehealth During the COVID-19 Pandemic. Avaialble online at: https://services. aap. org/en/pages/2019-novel-coronavirus-covid-19-infections/clinical-guidance/guidance-on-the-necessary-use-of-telehealth-during-the-covid-19-pandemic/<br>NICE: Guidance available but not specific to inpatient care<br>England NH, Improvement NH. Clinical guide for the management of remote consultations and remote working in secondary care during the coronavirus pandemic.<br><br>No studies identified<br><br>Verdict: No evidence found. **The question has NOT been addressed in the evidence base**	1 x physical therapist<br>1 x nurse<br>1 x physician
42	Does collecting and assessing patient-reported and patient-important outcomes for all hospitalized children/youth on a general paediatric inpatient unit improve outcomes?	Defining patient important outcomes from the get go	CPS: No guidelines identified.<br>AAP: No guidelines identified.<br>NICE: No guidelines identified.<br><br>Bele, S., Chugh, A., Mohamed, B., Teela, L., Haverman, L., & Santana, M. J. (2020). Patient-Reported Outcome Measures in Routine Pediatric Clinical Care: A Systematic Review. Frontiers in pediatrics, 8, 364.<br>Klaassen, R. J., Kinahan, J. Y., Graham, J. M., Hébert, Y. V., & O'Hearn, K. (2019). Patient-Reported Outcome Measures (PROMs) in Pediatric Non-Malignant Hematology: A Systematic Review.<br>Mistry, P., Stirling, H., Callens, C., Hodson, J., Batchelor, H., & SPaeDD-UK project (2018). Evaluation of patient-reported outcome measurements as a reliable tool to	1 x physician

© 2022 Gill PJ et al. *JAMA Network Open*.

			measure acceptability of the taste of paediatric medicines in an inpatient paediatric population. BMJ open, 8(7), e021961. https://doi.org/10.1136/bmjopen-2018-021961<br><br>Verdict: The strongest evidence comes from review by Bele et al. But the results are inconclusive. The study by Mistry et. al, is not a review, but it establishes the use of PROMs as a tool. Included studies are not focused on inpatient setting. So, no evidence. **The question has NOT been addressed in the evidence base**	
43	What are best practices when using intravenous immune globulin (IVIG) in hospitalized children/youth on the general paediatric inpatient unit?	Is it really necessary to flush the iv tubing line between ivig with different lot numbers? Since delayed infusion reactions can happen and you wouldnt be able to tell if it was a delayed reaction from the first lot number or a reaction from the second.	CPS: No guidelines identified.<br>AAP: No guidelines identified.<br>NICE: No guidelines identified.<br><br>Ohlsson, A., & Lacy, J. B. (2013). Intravenous immunoglobulin for preventing infection in preterm and/or low birth weight infants. Cochrane Database of Systematic Reviews, (7).<br>Robinson, J., Hartling, L., Vandermeer, B., Sebastianski, M., & Klassen, T. P. (2020). Intravenous immunoglobulin for presumed viral myocarditis in children and adults. Cochrane Database of Systematic Reviews, (8).<br>Iro, M. A., Martin, N. G., Absoud, M., & Pollard, A. J. (2017). Intravenous immunoglobulin for the treatment of childhood encephalitis. Cochrane Database of Systematic Reviews, (10).<br><br>Verdict: Reviews were found that looked at use of IVIG on various clinical conditions, but no studies were found that focused on best practices. Hence, this query was not addressed in the evidence base. **The question has NOT been addressed in the evidence base**	1 x nurse
44	Does the implementation of quality indicators lead to improved safety and reduced medical errors in the general paediatric inpatient unit?<br><br>(e.g. communication with medical team, medication errors, patient safety reviews, etc.)	best practice safety team<br>3. What quality & safety indicators should paediatric units across Canada be measuring regularly? Readmission rates? Medication errors? Compliance with follow-up? Communication with the community physician? Delay to obtain certain diagnostic testing? etc.<br>Are there any common illnesses/complications that arise in young people being treated on a general paediatric unit?<br>What can be done to ensure the most appropriate safety measures are maintained while providing safe medical care?<br>Regarding the the care of children in hospital on the general paediatric ward, I believe research should focus on the area of Paediatric Patient Safety and in particular: a) Best practices for eliminating hospital acquired conditions (HACs)<br>How to reduce medical errors,<br>Lack of quality assurance work<br>how can rural hospitals ensure they are providing adequate care Is there a way to measure the quality of the communication between disciplines in patient care?	CPS: No guidelines identified.<br>AAP: No guidelines identified.<br>NICE: No guidelines identified.<br><br>Jabbari, H., Sabet, S. A., & Heidarzadeh, M. (2015). Hospital Care for Newborn Babies: Quality Assessment, A Systematic Review. Iranian journal of pediatrics, 25(5).<br>Stelfox, H. T., Bobranska-Artiuch, B., Nathens, A., & Straus, S. E. (2010). A systematic review of quality indicators for evaluating pediatric trauma care. Critical care medicine, 38(4), 1187-1196.<br><br>Verdict: Articles founds only identify that quality indicators are necessary and list them out, but do not provide evidence on the benefits of its implementation. **The question has NOT been addressed in the evidence base**	3 x nurse<br>2 x physician<br>1 x youth<br>1 x patient safety research/web content editor<br>1 physical therapist
45	What are effective alternatives to shorten length of stay for hospitalized children/youth?<br><br>Eg, hospitalization at home, early discharge with close and regular follow-up), in the	How can we shorten LOS in a safe way ? Could we try home hospitalization in paediatric as it is done in adult ?<br>which inpatients could be safely and effectively cared for as outpatients with very close (up to daily) follow up care?<br>Does length of stay affect functional and rehabilitation outcomes?	CPS: no guidelines found<br>AAP: no guidelines found<br>NICE: no guidelines found<br><br>Miah, R. (2013). Does transitional care improve neonatal and maternal health outcomes? A systematic review. British Journal of Midwifery, 21(9), 634-646.<br>Segers, E., Ockhuijsen, H., Baarendse, P., van Eerden, I., & van den Hoogen, A. (2019). The impact of family centred care interventions in a neonatal or paediatric intensive care unit on parents' satisfaction and length of stay: a systematic review. Intensive and Critical Care Nursing, 50, 63-70.	2 x physician<br>1 x speech language pathologist

© 2022 Gill PJ et al. *JAMA Network Open*.

	general paediatric in-patient unit		Verdict: Some evidence was identified to support transitional care for neonates, but the studies focus on NICU or PICU. **The question has NOT been addressed in the evidence base**	
46	What is the most effective way to perform vital sign measurements while ensuring patient and family comfort on the general paediatric inpatient unit?	During one of my admissions, my vitals had to be checked every half hour by a nurse. This made it difficult to for both me and my parent to sleep. Is there a way to perform these checks at night without waking up the patient and parent?<br>FREQUENCY OF VITALS MONITORING, ESPECIALLY OVERNIGHT<br>how can we minimize resources utilized for inpatients without sacrificing safety-ie checking vital signs at night, weighing diapers, use of cardioresp monitors?<br>What should be the reasoning for putting a patient on a cardiac monitor?	CPS: no guidelines found<br>AAP: no guidelines found<br>NICE: no guidelines found<br><br>Van Kuiken, D., & Huth, M. M. (2013). What is 'normal?' Evaluating vital signs. Pediatric nursing, 39(5), 216–224.<br><br>Verdict: The only identified systematic review identifies limited evidence, and that further research is needed on this topic. **The question has NOT been addressed in the evidence base**	1 x youth<br>2 x nurse<br>1 x physician
47	What mental health supports can be provided to parents, families and children/youth while hospitalized on the general paediatric inpatient unit?	can we improve comfort for baby/ anxiety for parents In bronchiolitis ,<br>How hospitalization impacts anxiety levels in children - short and long term<br>How can we meet mental health needs of children and youth admitted to hospital?<br>what mental health supports can we put in place for kids before they need it?<br>Overall support of parent and mental health of child. It can be very isolating, especially if infection procedures in place<br>What help can we provide for parent for their mental health during this stressful time?<br>I wonder how we can reduce the stress of parents when a child is admitted with a complex formula (mix of powdered formula, modular +) +/- treated with medication (example of Kayexalate to remove potassium)<br>How are we socially supporting children and youth during this difficult period? i.e encouraging social groups or friendship formation?<br>2. Model of care whereby we get infants out of their cribs to play & move rather than providing screen time (for which psychologists recommend 0 screen time under the age of 2 years (we could measure how much screen time they were getting before and after the intervention)<br>Deconditioning is a reality of patients during their hospital stay.<br>4. At one point during my treatment, I was convinced that the "hospital smell" was choking me whenever I entered the building. What are some strategies that nurses, doctors, child life specialists and therapists can use to help kids with hospital anxiety or imaginary fears?	CPS: no guidelines found<br>AAP: no guidelines found<br>NICE: no guidelines found<br><br>McCormick, R. (2017). Does access to green space impact the mental well-being of children: A systematic review. Journal of Pediatric Nursing, 37, 3-7.<br>Mirghafourvand, M., Ouladsahebmadarek, E., Hosseini, M. B., Heidarabadi, S., Asghari-Jafarabadi, M., & Hasanpour, S. (2017). The effect of creating opportunities for parent empowerment program on parent's mental health: A systematic review. Iranian Journal of Pediatrics, 27(2). [NICU based]<br>Zhang, Q., Wu, J., Sheng, X., & Ni, Z. (2021). Empowerment programs for parental mental health of preterm infants: A meta-analysis. Patient Education and Counseling.<br>Bieleninik, Ł., Konieczna-Nowak, L., Knapik-Szweda, S., & Kwaśniok, J. (2020). Evaluating feasibility of the LongSTEP (Longitudinal Study of music Therapy's Effectiveness for Premature infants and their caregivers) protocol with a Polish cohort. Nordic Journal of Music Therapy, 29(5), 437-459.<br>Kasparian, N. A., Kan, J. M., Sood, E., Wray, J., Pincus, H. A., & Newburger, J. W. (2019). Mental health care for parents of babies with congenital heart disease during intensive care unit admission: Systematic review and statement of best practice. Early human development, 139, 104837. [ICU based]<br>Bernie C, Mitchell M, Williams K, May T. Parent-directed intervention versus controls whilst their child waits for diagnostic assessment: a systematic review protocol. Systematic reviews. 2021 Dec;10(1):1-8.<br>Facchini, M., & Ruini, C. (2020). The role of Music Therapy in the treatment of children with cancer: a systematic review of literature. Complementary Therapies in Clinical Practice, 101289.<br><br>Verdict: The identified reviews suggest that green space and music therapy seem to have some benefit. However, all the identified studies are focused on critical care and McCormick study is focused on green space outside hospital. **The question has NOT been addressed in the evidence base**	2 x physician<br>1 x nurse practitioner<br>2 x friend/family member<br>1 x dietician<br>1 x parent<br>1 x physical therapist<br>1 x kinesiologist<br>1 x youth
48	What are evidence-based protocols for the safe management and discharge of hospitalized infants with jaundice on the general paediatric inpatient unit?	When can babies with jaundice be discharged without concern for rebound (a lot of American data in this area but few use it...could be KT study)?<br>management of the infant who is admitted for physiologic jaundice - level to treat, frequency of testing, follow up care and testing.<br>management of physiologic jaundice in the newborn infant - appropriate level to treat, risk factors, frequency of testing, follow up care and testing	CPS: guideline available<br>Barrington KJ, Sankaran K, Canadian Paediatric Society, Fetus and Newborn Committee. Guidelines for detection, management and prevention of hyperbilirubinemia in term and late preterm newborn infants. Paediatrics & Child Health. 2007 Jun 1;12(suppl_B):1B-2B.<br>AAP: guideline available<br>American Academy of Pediatrics Subcommittee on Hyperbilirubinemia. Management of hyperbilirubinemia in the newborn infant 35 or more weeks of gestation. Pediatrics. 2004 Jul;114(1):297-316.<br>NICE: guideline available	2 x physician

© 2022 Gill PJ et al. *JAMA Network Open*.

			National Institute for Health and Care Excellence (Great Britain). Jaundice in newborn babies under 28 days. National Institute for Health and Care Excellence (NICE); 2010. (https://www.nice.org.uk/guidance/cg98)<br><br>Lee Wan Fei, S., & Abdullah, K. L. (2015). Effect of turning vs. supine position under phototherapy on neonates with hyperbilirubinemia: a systematic review. Journal of clinical nursing, 24(5-6), 672-682.<br>Deshmukh, J., Deshmukh, M., & Patole, S. (2019). Probiotics for the management of neonatal hyperbilirubinemia: a systematic review of randomized controlled trials. The Journal of Maternal-Fetal & Neonatal Medicine, 32(1), 154-163.<br>Chen, Z., Zhang, L., Zeng, L., Yang, X., Jiang, L., Gui, G., & Zhang, Z. (2017). Probiotics supplementation therapy for pathological neonatal jaundice: a systematic review and meta-analysis. Frontiers in pharmacology, 8, 432.<br>Van Rostenberghe, H., Ho, J. J., Lim, C. H., & Abd Hamid, I. J. (2020). Use of reflective materials during phototherapy for newborn infants with unconjugated hyperbilirubinaemia. Cochrane Database of Systematic Reviews, (7).<br><br>Verdict: The guidelines clearly outline management and discharge strategies. The systematic reviews identified in the search were from before 2010. **The question has been addressed in the evidence base**	
49	What are effective uses of albumin infusions in hospitalized children/youth hospitalized on the general paediatric inpatient unit?	When is the use of albumin indicated for inpatients with low albumin, or generalized edema?	CPS: no guidelines identified<br>AAP: no guidelines identified<br>NICE: no guidelines identified<br><br>No studies identified<br><br>Verdict: No evidence was found relevant to the question. **The question has NOT been addressed in the evidence base**	1 x physician
50	What are best practices during respiratory viral season to reduce spread of nosocomial infections amongst and prevent outbreaks hospitalized children/youth on the general paediatric inpatient unit?	During viral season is it OK to make patients share rooms if they are hospitalized with different viral infections?<br>And if so, what tests or screening could be used to prevent outbreaks?	CPS: Guideline found is limited to physician's office.<br>Moore DL. Infection prevention and control in paediatric office settings. Paediatrics & child health. 2018 Nov 19;23(8):e176-90.<br>AAP: no guidelines found<br>NICE: no guidelines found<br><br>Curtis A, Moore Z, Patton D, O'Connor T, Nugent L. Does using a cellular mobile phone increase the risk of nosocomial infections in the Neonatal Intensive Care Unit: A systematic review. Journal of Neonatal Nursing. 2018 Oct 1;24(5):247-52.<br>Moffa M, Guo W, Li T, Cronk R, Abebe LS, Bartram J. A systematic review of nosocomial waterborne infections in neonates and mothers. International journal of hygiene and environmental health. 2017 Nov 1;220(8):1199-206.<br>Stapleton PJ, Murphy M, McCallion N, Brennan M, Cunney R, Drew RJ. Outbreaks of extended spectrum beta-lactamase-producing Enterobacteriaceae in neonatal intensive care units: a systematic review. Archives of Disease in Childhood-Fetal and Neonatal Edition. 2016 Jan 1;101(1):72-8.<br><br>Verdict: There are several systematic reviews which focus on the prevention and spread of nosocomial infection (bacterial and fungal), but none of them are specific to virus or viral season. Hence, the evidence was not found to address this query. **The question has NOT been addressed in the evidence base**	1 x youth<br>1 x respiratory therapist
51	When should physicians complete nasopharyngeal swabs for respiratory viral infections in children/youth	When is the use of a nasopharyngeal swab indicated on hospitalized children?	CPS: Guidelines related to COVID-19 were identified.<br>Nicole Le Saux, Canadian Paediatric Society; Current epidemiology and guidance for COVID-19 caused by SARS-CoV-2 virus, in children: March 2020; https://www.cps.ca/documents/position/current-epidemiology-and-guidance-for-covid-19-march-2020<br>Michael Narvey, Canadian Paediatric Society, Fetus and Newborn Committee; NICU	1 x physician

	hospitalized on a general paediatric inpatient unit?		care for infants born to mothers with suspected or confirmed COVID-19; https://www.cps.ca/documents/position/nicu-care-for-infants-born-to-mothers-with-suspected-or-proven-covid-19<br>AAP: no guidelines found<br>NICE: no guidelines found<br><br>Verdict: COVID-19 related guidelines were constantly changing and left to the discretion of each province. according to one guideline, "obtain a nasopharyngeal swab within 2 h of birth if SARS-CoV-2 has been confirmed in the mother, regardless of infant symptomatology". No evidence found for other viral infections. **The question has NOT been addressed in the evidence base**	
52	Can a national EMR system improve care on a general paediatric ward?	PROVINCIAL/NATIONAL EMR please :)<br>The trajectory of care prior to hospitalization (how often were they seen - by whom)<br>I wonder a lot about the trajectory of patients, before and especially after hospitalization, in terms of efficiency and quality of follow-up<br>Sharing order sets for common pediatric conditions (i.e. Kawasaki Disease, Sickle Cell, Sepsis, etc). --> NOT RELATED	CPS: no guidelines identified<br>AAP: no guidelines identified<br>NICE: no guidelines identified<br><br>Despins, L. A. (2017). Automated detection of sepsis using electronic medical record data: a systematic review. The Journal for Healthcare Quality (JHQ), 39(6), 322-333.<br><br>Verdict: The review by Despin, does attempt to address the question to some extent, but the results are inconclusive. The focus is also limited to sepsis care and does not employ a national EMR. **The question has NOT been addressed in the evidence base.**	4 x parent/caregiver
53	Does ensuring continuity of care of healthcare professionals improve the care and outcomes of hospitalized children/youth on the general paediatric inpatient unit?	9) How can we have better processes/structures to ensure the continuity of care beyond hospitalization?<br>there was very little continuity in the nurses who looked after our child?	CPS: Guideline on continuity of care: https://www.cpso.on.ca/admin/CPSO/media/Documents/public/public-information/coc-guide/coc-guide-patients-caregivers.pdf<br>AAP: no guidelines identified<br>NICE: no guidelines identified<br><br>Connolly M., Fortuna R.J., Snyder E.D. & Weppner W.G. (2019). Impacts of improved continuity of care in resident primary care clinics on patient outcomes: A systematic review. Journal of General Internal Medicine, 34, S254. https://doi.org/10.1007/11606.1525-1497 [ABSTRACT]<br>Lenton-Brym, T., Rodrigues, A., Johnson, N., Couturier, J., & Toulany, A. (2020). A scoping review of the role of primary care providers and primary care-based interventions in the treatment of pediatric eating disorders. Eating disorders, 28(1), 47-66.<br><br>Verdict: Evidences identified does not focus on the GPIU. **The question has NOT been addressed in the evidence base.**	2 x parent/caregiver
54	What are the adverse effects of sedation on children/youth hospitalized on a general paediatric ward?	Do the risks of sedation for a procedure outweigh the risks of developing trauma from a procedure in Pediatrics? How does one know?	CPS: Relevant position statement<br>Krmpotic K, Rieder MJ, Rosen D. Recommendations for procedural sedation in infants, children, and adolescents. Paediatrics & Child Health. 2021 Apr;26(2):128-.<br>AAP: no guidelines identified<br>NICE: guideline present but does not discuss adverse effects.<br>National Institute of health and excellence. Sedation in under 19s: using sedation for diagnostic and therapeutic procedures; www.nice.org.uk/guidance/cg112<br><br>Azzam, M. A., Elngar, E. F., Touny, S. A. A., Shaffik, A. T., Hussein, M. H., Sabahy, K. A., & Kamhawy, G. A. (2019). Daily sedation interruption versus routine sedation in critically ill children: A systematic review and meta-analysis. Egyptian Journal of Anaesthesia, 35(1), 77-85. [based on ICU]<br>Grant, M. J. C., Balas, M. C., Curley, M. A., & Team, R. I. (2013). Defining sedation-related adverse events in the pediatric intensive care unit. Heart & Lung, 42(3), 171-176. [based on ICU]	1 x nurse

© 2022 Gill PJ et al. *JAMA Network Open*.

			Verdict: Both systematic reviews are based on ICU population; none addressed the GPIU. **The question has NOT been addressed in the evidence base**	
55	What are the most effective diagnostic tests to diagnose meningitis in children/youth hospitalized on a general paediatric inpatient unit?	Use of MRI in meningitis - review of practices across the country. How do findings impact outcomes (in our centre many treatment alterations due to findings but are they clinically relevant? If they are should more centres use MRI routinely in meningitis)	CPS: Diagnosis guideline available<br>Le Saux N, Canadian Paediatric Society, Infectious Diseases and Immunization Committee. Guidelines for the management of suspected and confirmed bacterial meningitis in Canadian children older than one month of age. Paediatrics & child health. 2014 Mar 3;19(3):141-6.<br>Jefferies AL. Management of term infants at increased risk for early-onset bacterial sepsis. Paediatrics & child health. 2017 Jul 1;22(4):223-8.<br>AAP: no guidelines identified<br>NICE: no guidelines identified<br><br>Khandaker, G., Heron, L., Rashid, H., Li-Kim-Moy, J., Lester-Smith, D., Kesson, A., ... & Booy, R. (2013). Comparing the use of, and considering the need for, lumbar puncture in children with influenza or other respiratory virus infections. Influenza and other respiratory viruses, 7(6), 932-937.<br>Kulik, D. M., Uleryk, E. M., & Maguire, J. L. (2013). Does this child have bacterial meningitis? A systematic review of clinical prediction rules for children with suspected bacterial meningitis. The Journal of emergency medicine, 45(4), 508-519.<br>Mansouri, F., Sabhaney, V., Meckler, G., Doan, Q., & Burstein, B. (2020). 78 CONCOMITANT MENINGITIS AMONG FEBRILE YOUNG INFANTS 29-60 DAYS OLD WITH A POSITIVE URINALYSIS AND THE NEED FOR LUMBAR PUNCTURE: A SYSTEMATIC REVIEW AND META-ANALYSIS. Paediatrics & Child Health, 25(Supplement_2), e32-e33.<br>Heeyeon Kim, Yoon Ho No, Seo Hee Yoon. Blood procalcitonin level as a diagnostic marker of pediatric bacterial meningitis: a systematic review and meta-analysis. PROSPERO 2021 CRD42021186913 Available from: https://www.crd.york.ac.uk/prospero/display_record.php?ID=CRD42021186913<br>Monisha R, Sourabh Dutta, Praveen Kumar, Ashutosh Aggarwal. Diagnostic accuracy of cerebrospinal fluid c-reactive protein, procalcitonin and interleukin-6 for meningitis in neonates and young infants < 90 days old: a systematic review and meta-analysis. PROSPERO 2019 CRD42019126957 Available from: https://www.crd.york.ac.uk/prospero/display_record.php?ID=CRD42019126957<br><br>Verdict: Other then the CPS guideline, there is a lack of strong evidence to identify the most effective diagnostic test. No systematic review wa found that could support the question but several studies appear to be in progress. **The question has NOT been addressed in the evidence base.**	1 x physician
56	What are effective treatments for children/youth with acute kidney disease hospitalized on a general paediatric inpatient unit?	We see many children with acute kidney injuries, nephrotic syndrome, pyelonephritis and other.<br>I would like to see more research and treatment options for renal disease.	CPS: no guidelines identified<br>AAP: no guidelines identified<br>NICE: no guidelines identified<br><br>Alabbas, A., Kirpalani, A., Morgan, C., Mammen, C., Licht, C., Phan, V., ... & Lemaire, M. (2021). Canadian Association of Paediatric Nephrologists COVID-19 Rapid Response: Guidelines for Management of Acute Kidney Injury in Children. Canadian Journal of Kidney Health and Disease, 8, 2054358121990135.<br><br>Verdict: No guidelines or systematic review addressed this question. **The question has NOT been addressed in the evidence base**	1 x nurse
57	What is the most effective treatment for children/youth with complicated pneumonia	5) Management of large but minimally symptomatic complicated effusions<br>What is the appropriate post-pleurocentesis management for complicated pneumonia (when and how to Efficacy and safety of azithromycin combined with glucocorticoid on refractory	CPS: One guideline was identified focused on empyema<br>Chibuk TK, Cohen E, Robinson JL, Mahant S, Hartfield DS, Canadian Paediatric Society. Paediatric complicated pneumonia: diagnosis and management of empyema. Paediatrics & child health. 2011 Sep 1;16(7):425-7.<br>AAP: no guidelines identified	1 x physician

© 2022 Gill PJ et al. *JAMA Network Open*.

	hospitalized on a general paediatric inpatient unit?	Mycoplasma pneumoniae pneumonia in children: A PRISMA-compliant systematic review and meta-analysisreimage, clamp, go to straight drainage)	NICE: no guidelines identified<br><br>Al Saeedy, D., Gillani, S. W., Al-Salloum, J., Moosvi, A., Eissa, M., & Gulam, S. M. (2020). Comparative Efficacy of Beta-Lactams and Macrolides in the Treatment of Pediatric Pneumonia: A Systematic Review. Current pediatric reviews, 16(4), 307-313. [FULL article not found]<br>Das, R. R., & Singh, M. (2013). Treatment of severe community-acquired pneumonia with oral amoxicillin in under-five children in developing country: a systematic review. PloS one, 8(6), e66232.<br>Das, R. R., Singh, M., Panigrahi, I., & Naik, S. S. (2013). Vitamin D supplementation for the treatment of acute childhood pneumonia: a systematic review. International Scholarly Research Notices, 2013.<br>Haider, B. A., Lassi, Z. S., Ahmed, A., & Bhutta, Z. A. (2011). Zinc supplementation as an adjunct to antibiotics in the treatment of pneumonia in children 2 to 59 months of age. Cochrane database of systematic reviews, (10).<br>Tie, H. T., Tan, Q., Luo, M. Z., Li, Q., Yu, J. L., & Wu, Q. C. (2016). Zinc as an adjunct to antibiotics for the treatment of severe pneumonia in children< 5 years: a meta-analysis of randomised-controlled trials. British Journal of Nutrition, 115(5), 807-816.<br>Hemilä, H., & Louhiala, P. (2013). Vitamin C for preventing and treating pneumonia. Cochrane database of systematic reviews, (8).<br>Hu, N., Li, Q. B., & Zou, S. Y. (2018). Effect of vitamin A as an adjuvant therapy for pneumonia in children: a meta analysis. Zhongguo Dang dai er ke za zhi= Chinese Journal of Contemporary Pediatrics, 20(2), 146-153. [In Chinese]<br>Wu, T., Ni, J., & Wei, J. (2005). Vitamin A for non-measles pneumonia in children. Cochrane Database of Systematic Reviews, (3).<br>Mu, S. Y., Zou, Y. X., Zhai, J., & Yao, G. H. (2020). Efficacy and safety of vitamin D as adjuvant therapy for childhood pneumonia: a Meta analysis. Zhongguo Dang dai er ke za zhi= Chinese Journal of Contemporary Pediatrics, 22(2), 124-129.[In Chinese]<br>Qiu, J. L., Huang, L., Shao, M. Y., Chai, Y. N., Zhang, H. J., Li, X. F., ... & Zhai, W. S. (2020). Efficacy and safety of azithromycin combined with glucocorticoid on refractory Mycoplasma pneumoniae pneumonia in children: A PRISMA-compliant systematic review and meta-analysis. Medicine, 99(22), e20121.<br>Zhang, G. M., Huang, Z. Y., Sun, R., Ye, S. L., & Feng, Q. (2020). Xiao’er Xiaoji Zhike Oral Liquid Combined with Azithromycin for Mycoplasma pneumoniae Pneumonia in Children: A Systematic Review and Meta-Analysis. Evidence-Based Complementary and Alternative Medicine, 2020.<br>Kim, H. S., Sol, I. S., Li, D., Choi, M., Choi, Y. J., Lee, K. S., ... & Kim, H. H. (2019). Efficacy of glucocorticoids for the treatment of macrolide refractory mycoplasma pneumonia in children: meta-analysis of randomized controlled trials. BMC pulmonary medicine, 19(1), 1-14.<br>Lodha, R., Kabra, S. K., & Pandey, R. M. (2013). Antibiotics for community-acquired pneumonia in children. Cochrane Database of Systematic Reviews, (6).<br>Lodha, R., Randev, S., & Kabra, S. K. (2016). Oral antibiotics for community–acquired pneumonia with chest-indrawing in children aged below five years: A Systematic Review. Indian pediatrics, 53(6), 489-495.<br>Mendonça, K., Chaves, G., Guerra, I., Freitas, D., Macêdo, T., Mendes, R., ... & Fregonezi, G. (2014). Chest physiotherapy for children with pneumonia: A systematic review. European Respiratory Journal, 44(Suppl 58).<br>Redden M.D., Chin T.Y. & van Driel M.L. (2017). Surgical versus non-surgical management for pleural empyema. Cochrane Database of Systematic Reviews, 2017, no pagination. https://doi.org/10.1002/14651858.CD010651.pub2<br><br>Verdict: Several systematic reviews were identified which focus on medications for	

© 2022 Gill PJ et al. *JAMA Network Open*.

			pneumonia in children. Other reviews study vitamins, mainly related to developing countries, including Vitamin A, D, C, Zinc, and Azithromycin. Another review by Redden et al. identifies that video-assisted thoracoscopic surgery may reduce length of hospital stay compared to thoracostomy drainage alone. However, the question of medical vs interventional therapy has not been addressed. **The question has NOT been addressed in the evidence base**	
58	What is the most effective treatment for children/youth with aspiration pneumonia hospitalized on a general paediatric inpatient unit?	What is the appropriate treatment for aspiration pneumonia (if any) including choice of antibiotics and length of therapy.	CPS: no guidelines identified<br>AAP: no guidelines identified<br>NICE: no guideline identified<br><br>Liu, B., Li, S. Q., Zhang, S. M., Xu, P., Zhang, X., Zhang, Y. H., ... & Zhang, W. H. (2013). Risk factors of ventilator-associated pneumonia in pediatric intensive care unit: a systematic review and meta-analysis. Journal of thoracic disease, 5(4), 525.<br>de Neef, M., Bakker, L., Dijkstra, S., Raymakers-Janssen, P., Vileito, A., & Ista, E. (2019). Effectiveness of a ventilator care bundle to prevent ventilator-associated pneumonia at the PICU: a systematic review and meta-analysis. Pediatric Critical Care Medicine| Society of Critical Care Medicine, 20(5), 474-480.<br>Rose, L., Adhikari, N. K., Leasa, D., Fergusson, D. A., & Mckim, D. (2017). Cough augmentation techniques for extubation or weaning critically ill patients from mechanical ventilation. Cochrane Database of Systematic Reviews, (1).<br><br>Verdict: The identified studies focus primarily on ventilator induced pneumonia, which focuses on the ICU. None are relevant to GPIU and aspiration pneumonia. **The question has NOT been addressed in the evidence base**	1 x physician
59	Does including interpreters for all communications between healthcare professionals and families/patients improve care for hospitalized children on the general paediatric inpatient unit?	One of the most challenging aspects of my work is communicating with people who do not speak either English or French. Ottawa has a big immigrant population and yes the interpreters are present for admission procedures but day to day care, communication, directions and support are difficult to provide.<br>Availability of care in the preferred language of family (ex: French speaking staff for French speaking families).	CPS: Guideline present on crosscultural communication<br>Ladha T, Zubairi M, Hunter A, Audcent T, Johnstone J. Cross-cultural communication: Tools for working with families and children. Paediatrics & child health. 2018 Feb 15;23(1):66-9.<br>AAP: no guidelines identified<br>NICE: no guideline identified<br><br>Boylen, S., Cherian, S., Gill, F. J., Leslie, G. D., & Wilson, S. (2020). Impact of professional interpreters on outcomes for hospitalized children from migrant and refugee families with limited English proficiency: a systematic review. JBI Evidence Synthesis, 18(7), 1360-1388.<br><br>Verdict: There is evidence that use of ad hoc interpreters or no interpreter is inferior to use of professional interpreters of any mode. **The question has been addressed in the evidence base**	2 x nurse
60	What are adverse effects of nebulized epinephrine on patients, caregivers and healthcare professionals on the general paediatric inpatient unit?	The Second hand effect of inhalation epinephrine on the Nurse or parent holding the nebulizer mask. Is it safe in pregnancy? Does it increase caregivers HR? Cause headaches? Cause sleeplessness later in day? How can these risks be minimized?	CPS: Guideline available that suggests epinephrine has no adverse effects on patients (it is rather indicated for croup management).<br>Ortiz-Alvarez O. Acute management of croup in the emergency department. Paediatrics & child health. 2017 Jun 1;22(3):166-9<br>AAP: no guidelines identified<br>NICE: no guidelines identified<br><br>Baggott, C., Hardy, J., Sparks, J., Sabbagh, D., Beasley, R., Weatherall, M., & Fingleton, J. (2020). Systematic review of the comparison between adrenaline (epinephrine) and selective ß2-agonist in the setting of adults or children with acute asthma. [full article not found]<br>Bjornson, C., Russell, K. F., Vandermeer, B., Durec, T., Klassen, T. P., & Johnson, D. W. (2011). Nebulized epinephrine for croup in children. Cochrane database of systematic reviews, (2).<br>Chime, N. O., Riese, V. G., Scherzer, D. J., Perretta, J. S., McNamara, L., Rosen, M. A., & Hunt, E. A. (2017). Epinephrine auto-injector versus drawn up epinephrine for	1 x nurse

© 2022 Gill PJ et al. *JAMA Network Open*.

			anaphylaxis management: a scoping review. Pediatric Critical Care Medicine, 18(8), 764-769.<br>Isayama, T., Mildenhall, L., Schmölzer, G. M., Kim, H. S., Rabi, Y., Ziegler, C., & Liley, H. G. (2020). The route, dose, and interval of epinephrine for neonatal resuscitation: a systematic review. Pediatrics, 146(4).<br>Ohshimo, S., Wang, C. H., Couto, T. B., Bingham, R., Mok, Y. H., Kleinman, M., ... & Meaney, P. A. (2021). Pediatric timing of epinephrine doses: A systematic review. Resuscitation.<br><br>Verdict: There have been several systematic reviews focused on the use of nebulised epinephrine. In terms of adverse effect the review by Bjornson et. al. suggests there were no adverse effect to report. But that evidence is limited to children suffering from croup. There is limited evidence on the safety for healthcare professionals and caregivers. **The question has been PARTIALLY addressed in the evidence base**	
61	How are ethical principles and guidelines influencing the care of hospitalized children on the general paediatric inpatient unit?	More research on ethical dilemmas in pediatrics (quality of life, etc).	CPS: no guidelines identified<br>AAP: no guidelines identified<br>NICE: no guidelines identified<br><br>Janvier, A., Prentice, T., Gillian, L., & Davis, P. (2016). E03-A Who is the victim? A systematic review and ethical analysis of moral distress in pediatric intensive care units. Journal of Pain and Symptom Management, 52(6), e39-e40.<br>Mooney-Doyle, K., & Ulrich, C. M. (2020). Parent moral distress in serious pediatric illness: A dimensional analysis. Nursing ethics, 27(3), 821-837.<br>Prentice, T., Janvier, A., Gillam, L., & Davis, P. G. (2016). Moral distress within neonatal and paediatric intensive care units: a systematic review. Archives of disease in childhood, 101(8), 701-708.<br><br>Verdict: All the current studies are focused on PICU patients, none on children in GPIU. **The question has NOT been addressed in the evidence base**	1 x nurse
62	What is the effectiveness of oseltamivir for hospitalized children/youth with influenza on a general paediatric ward?	Which hospitalized children with influenza benefit from oseltamivir?	CPS: guideline suggest they are effective anti-virals<br>Upton D. Allen; Canadian Pediatric Society, Infectious Diseases and Immunization Committe. The use of antiviral drugs for influenza: Guidance forpractitioners. https://www.cps.ca/documents/position/antiviral-drugs-for-influenza<br>AAP: guideline suggest they are effective anti-virals<br>Committee on Infectious Diseases. Recommendations for prevention and control of influenza in children, 2018–2019. Pediatrics. 2018 Oct 1;142(4).<br>NICE: guideline suggest they are effective anti-virals.<br>National institue of Helath and care excellence. Amantadine, oseltamivir and zanamivirAmantadine, oseltamivir and zanamivirfor the treatment of influenzafor the treatment of influenza; www.nice.org.uk/guidance/ta168<br><br>Jefferson, T., Jones, M., Doshi, P., Spencer, E. A., Onakpoya, I., & Heneghan, C. J. (2014). Oseltamivir for influenza in adults and children: systematic review of clinical study reports and summary of regulatory comments. Bmj, 348.<br>Michiels, B., Van Puyenbroeck, K., Verhoeven, V., Vermeire, E., & Coenen, S. (2013). The value of neuraminidase inhibitors for the prevention and treatment of seasonal influenza: a systematic review of systematic reviews. PloS one, 8(4), e60348.<br>Shim, S., Chan, M., Owens, L., Jaffe, A., Prentice, B., & Homaira, N. (2020, June). OSELTAMIVIR FOR INFLUENZA TREATMENT IN CHILDREN WITH CHRONIC LUNG DISEASES. In RESPIROLOGY (Vol. 25, pp. 225-225). 111 RIVER ST, HOBOKEN 07030-5774, NJ USA: WILEY. [no article found]<br>Smieja, M. (2012). Oseltamivir relieves symptoms but does not reduce hospitalizations in influenza. Annals of internal medicine, 157(6), JC3-5.<br>Ryan E. Malosh, Emily T. Martin. Oseltamivir treatment for influenza in children: a	1 x physician

© 2022 Gill PJ et al. *JAMA Network Open*.

			meta-analysis of randomised controlled trials. PROSPERO 2016 CRD42016038982 Available from: https://www.crd.york.ac.uk/prospero/display_record.php?ID=CRD42016038982 [in progress]<br><br>Verdict: There seems sufficient work has been done in this field and oseltamivir is effective in children hospitalized with influenza. But, the strong evidence (e.g. RCTs) are. based on outpatient setting, and the inpatient setting is based on low quality observational, mainly retrospective studies. **The question has NOT been addressed in the evidence base**	
63	What are effective tools to increase adherence with recommended therapies for hospitalized children/youth on a general paediatric ward?	How to improve compliance with treatment.)	CPS: no guideline identified<br>AAP: no guideline identified<br>NICE: no guideline identified<br><br>Chistol, I., Tan, Z., Mohamed, S., Mohamed, S., Halim, N. A., & Morris, V. (2019). P163 Adherence to the bronchiolitis guidelines-a departmental study between November 2018-january 2019.<br>Coffman, J. M., Cabana, M. D., Bekmezian, A., Cho, C., Hersh, A., Okumura, M., & Patel, M. R. (2010). A Systematic Review Of Interventions To Improve The Prescribing Of Asthma Medications To Children. D40. INNOVATIVE APPROACHES AND NEW DEVELOPMENTS IN LUNG HEALTH RESEARCH, A5801-A5801. [article not found]<br>Frogel, M. (2010). A systematic review of compliance with palivizumab administration for RSV immunoprophylaxis. Journal of Managed Care Pharmacy, 16(1), 46-58.<br>Okelo, S. O., Butz, A. M., Sharma, R., Diette, G. B., Pitts, S. I., King, T. M., ... & Robinson, K. A. (2013). Interventions to modify health care provider adherence to asthma guidelines: a systematic review. Pediatrics, 132(3), 517-534.<br><br>Verdict: The available evidence does not provide any direction to which tools increase adherence. **The question has NOT been addressed in the evidence base**	1 x physician
64	What is the effectiveness of procalcitonin in diagnosing a serious bacterial infection in children/youth hospitalized in a general paediatric inpatient unit?	Is procalcitonin effective to diagnose serious bacterial infections?	CPS: procalcitonin does diagnose early onset of bacterial sepsis.<br>Jefferies AL. Management of term infants at increased risk for early-onset bacterial sepsis. Paediatrics & child health. 2017 Jul 1;22(4):223-8.<br>AAP: no guideline identified<br>NICE: guideline found but limited to ED and ICU<br><br>Bell, J. M., Shields, M. D., Agus, A., Dunlop, K., Bourke, T., Kee, F., & Lynn, F. (2015). Clinical and cost-effectiveness of procalcitonin test for prodromal meningococcal Disease–A meta-analysis. PLoS One, 10(6), e0128993.<br>Ritchie, B., Porritt, K., Marin, T., & Williams, N. (2020). Diagnostic test accuracy of serum measurement of procalcitonin and C-reactive protein for bone and joint infection in children and adolescents: a systematic review protocol. JBI evidence synthesis, 18(3), 564-570. [protocol]<br>Schuetz, P., Muller, B., Christ-Crain, M., Stolz, D., Tamm, M., Bouadma, L., ... & Briel, M. (2013). Procalcitonin to initiate or discontinue antibiotics in acute respiratory tract infections. Evidence-Based Child Health: A Cochrane Review Journal, 8(4), 1297-1371. [does not cover inpatient care]<br><br>Verdict: For certain infections (meningitis) evidence has been found that suggests procalcitonin is effective in the initial diagnosis stage. There is some evidence for meningitis, procalcitonin can be helpful, but evidence is limited for other conditions, and regarding its use in the GPIU. **The question has been PARTIALLY addressed in the evidence base**	1 x physician

© 2022 Gill PJ et al. *JAMA Network Open*.

65	What are medication-related information management system or tool improves outcomes of children/youth hospitalized in a general paediatric inpatient unit?	Pharmacokinetics of drugs in children.<br>Who can we contact if we have a question about meds?	CPS: no guidelines identified<br>AAP: no guidelines identified<br>NICE: no guidelines identified<br><br>No studies identified<br><br>Verdict: No studies identified. **The question has NOT been addressed in the evidence base**	1 x physician<br>1 x parent/caregiver
66	Can a national network improve care for children/youth on the general paediatric inpatient unit?	A national collaborative could help to improve quality care of hospitalized children nationwide.	CPS: no guidelines identified<br>AAP: no guidelines identified<br>NICE: no guidelines identified<br><br>No studies identified<br><br>Verdict: No studies identified. The question has NOT been addressed in the evidence base	1 x physician
67	What are the most effective methods for oral and nasal suctioning (i.e aggressive vs minimalist, cough assist vs deep suctioning) of hospitalized children/youth on a general paediatric inpatient unit?	could cough assist (in/exsulflator) be used more instead of deep suctioning to help clear secretions?<br>In particular, what is the value of aggressive vs. minimalist approaches to suctioning.	CPS: suction during resuscitation and Bronchiolitis management<br>Friedman JN, Rieder MJ, Walton JM, Canadian Paediatric Society, Acute Care Committee, Drug Therapy and Hazardous Substances Committee. Bronchiolitis: recommendations for diagnosis, monitoring and management of children one to 24 months of age. Paediatrics & child health. 2014 Nov 3;19(9):485-91.<br>Finan E, Campbell DM, Aziz K, McNamara PJ. Update for Canadian NRP providers: A case-based review. Paediatrics & child health. 2017 Sep 1;22(6):351-3.<br>AAP: no guidelines identified<br>NICE: acknowledges that there is no evidence<br><br>No studies identified<br><br>Verdict: No studies identified. The question has NOT been addressed in the evidence base	2 x physician
68	What are non-antimicrobial therapies for hospitalized children/youth with chronic otitis media on the general paediatric inpatient unit?	alternative options to antibiotic for children with Chronic ear infections	CPS: guideline found is on acute otitis media. Nothing found on chronic<br>Le Saux N, Robinson JL, Canadian Paediatric Society, Infectious Diseases and Immunization Committee. Management of acute otitis media in children six months of age and older. Paediatrics & child health. 2016 Feb 5;21(1):39-44.<br>AAP: guideline found is on acute otitis media. Nothing found on chronic<br>NICE: guideline found is on acute otitis media. Nothing found on chronic<br>National Institute of Health and Care Excellence; Otitis media (acute): antimicrobial prescribing; www.nice.org.uk/guidance/ng91<br><br>Head, K., Chong, L. Y., Bhutta, M. F., Morris, P. S., Vijayasekaran, S., Burton, M. J., Schilder, A. G., & Brennan-Jones, C. G. (2020). Topical antiseptics for chronic suppurative otitis media. The Cochrane database of systematic reviews, 1(1), CD013055.<br>Ranakusuma, R. W., Pitoyo, Y., Safitri, E. D., Thorning, S., Beller, E. M., Sastroasmoro, S., & Del Mar, C. B. (2018). Systemic corticosteroids for acute otitis media in children. The Cochrane database of systematic reviews, 3(3), CD012289. https://doi.org/10.1002/14651858.CD012289.pub2<br><br>Verdict: Only one systematic review found that looked at use of antiseptics. But the evidence is limited and are based on outpatient care. **The question has NOT been addressed in the evidence base**	1 x parent/caregiver
69	What are effective treatments for children/youth and	Eating disorders,<br>the organized diagnostic approach of eating disorders, for example, crying on food and many others	CPS: guideline are not directed towards inpatient care (family based treatment)<br>Norris ML, Hiebert JD, Katzman DK. Determining treatment goal weights for children and adolescents with anorexia nervosa. Paediatrics & child health. 2018 Nov	1 x nurse<br>1 x parent/caregiver

© 2022 Gill PJ et al. *JAMA Network Open*.

	adolescents hospitalized with eating disorders on the general paediatric inpatient unit?		19;23(8):551-.<br>Findlay S, Pinzon J, Taddeo D, Katzman DK, Canadian Paediatric Society, Adolescent Health Committee. Family-based treatment of children and adolescents with anorexia nervosa: Guidelines for the community physician. Paediatrics & child health. 2010 Jan 1;15(1):31-5.<br>AAP: no guideline identified<br>NICE: detailed guideline available but treatments are not confined to inpatient care<br>NICE guideline, Eating disorders: recognition and treatment; http://www.nice.org.uk/guidance/ng69<br><br>Gelin, Z., Cook-Darzens, S., Simon, Y., & Hendrick, S. (2016). Two models of multiple family therapy in the treatment of adolescent anorexia nervosa: a systematic review. Eating and Weight Disorders-Studies on Anorexia, Bulimia and Obesity, 21(1), 19-30. [family therapy models, not inpatient care]<br>Hay, P. J., Touyz, S., Claudino, A. M., Lujic, S., Smith, C. A., & Madden, S. (2019). Inpatient versus outpatient care, partial hospitalisation and waiting list for people with eating disorders. Cochrane Database of Systematic Reviews, (1).<br>Herpertz, S., Hagenah, U., Vocks, S., von Wietersheim, J., Cuntz, U., & Zeeck, A. (2011). The diagnosis and treatment of eating disorders. Deutsches Ärzteblatt International, 108(40), 678. [not a systematic review]<br>Isserlin, L., Spettigue, W., Norris, M., & Couturier, J. (2020). Outcomes of inpatient psychological treatments for children and adolescents with eating disorders at time of discharge: a systematic review. Journal of eating disorders, 8(1), 1-17.<br>Lyza Norton, Joy Parkinson, Neil Harris, Laura Hart, Morgan Cole. Food communication practices for the prevention of disordered eating (“problems with eating, body image and weight”) in children – a systematic review.. PROSPERO 2020 CRD42020201141 Available from: https://www.crd.york.ac.uk/prospero/display_record.php?ID=CRD42020201141 [In-progress]<br>Tora Margrethe Hustad, Ida Maria Brennhaugen, Benjamin Andersen Sandoval, Ingrid Borren, Astrid Austvoll-Dahlgren, Silje Steinsbekk. Effects of interventions for eating disorders in children and adolescents: An overview of systematic reviews.. PROSPERO 2020 CRD42020169210 Available from: https://www.crd.york.ac.uk/prospero/display_record.php?ID=CRD42020169210 [In-progress]<br>Laura Reale, Carlotta Cirone, Benedetto Vitiello, Federico Amianto, Chiara Davico, Federica Ricci. Antipsychotic drugs for eating disorders in children and adolescents: a systematic review and meta-analysis. PROSPERO 2019 CRD42019145595 Available from: https://www.crd.york.ac.uk/prospero/display_record.php?ID=CRD42019145595 [In-progress]<br><br>Verdict: Though some work has been done in this field, the question is still unanswered. There seems to be a lot of variation in treatment and no systematic review has identified the most appropriate modality. **The question has NOT been addressed in the evidence base**	
70	What tools or techniques can increase the success rate of lumbar punctures in children and youth hospitalized on a general paediatric inpatient unit?	Predictors of difficult LP and/or use of POCUS to optimize LP success rates	CPS: no guidelines identified, other than focused on pain management<br>Trottier ED, Doré-Bergeron MJ, Chauvin-Kimoff L, Baerg K, Ali S. Managing pain and distress in children undergoing brief diagnostic and therapeutic procedures. Paediatrics & child health. 2019 Dec 9;24(8):509-21.<br>AAP: no guideline identified.<br>NICE: no guideline identified<br><br>Zummer, J., Desjardins, M. P., Séguin, J., Roy, M., & Gravel, J. (2020). Emergency physician performed ultrasound-assisted lumbar puncture in children: A randomized	1 x physician

© 2022 Gill PJ et al. *JAMA Network Open*.

			controlled trial. The American journal of emergency medicine. [not a systematic review]—ultrasound -> high success<br>Foster, J. P., Taylor, C., & Spence, K. (2017). Topical anaesthesia for needle-related pain in newborn infants. Cochrane Database of Systematic Reviews, (2). [Systematic review] -> results are inconclusive<br>Kirschner, J., & Hunter, B. (2019). Ultrasound-assisted lumbar puncture (LP) does not increase procedural success but reduces traumatic LPs. Acp Journal Club, 170(2). [review]<br>Gottlieb, M., Holladay, D., & Peksa, G. D. (2019). Ultrasound-assisted lumbar punctures: a systematic review and meta-analysis. Academic Emergency Medicine, 26(1), 85-96.<br>Shaikh F, Brzezinski J, Alexander S, Arzola C, Carvalho JC, Beyene J, Sung L. Ultrasound imaging for lumbar punctures and epidural catheterisations: systematic review and meta-analysis. Bmj. 2013 Mar 26;346.<br>Abiola Olowoyeye, Charles Okwundu, Opeyemi Fadahunsi, Jerome Okudo, Kare Opaneye. Ultrasound imaging compared with anatomical for pediatric lumbar puncture. PROSPERO 2017 CRD42017055800 Available from: https://www.crd.york.ac.uk/prospero/display_record.php?ID=CRD42017055800 [In-progress]<br><br>Verdict: Some work has been done, but the results are inconclusive. The systematic review by Shaikh does find some evidence in favour of ultrasound assisted lumbar puncture, but the pediatric population was negligible in the review. While the question has been studied, the overall evidence is uncertain. **TThe question has NOT been addressed in the evidence base**	
71	What are risk factors for serious underlying illnesses in children who are hospitalized with a brief resolved unexplained event (BRUE) on the general paediatric inpatient unit?	How can we differentiate between children who will have a one time BRUE and kids who have larger medical issues?	CPS: No guideline identified<br>AAP: Relevant guideline identified which outlines some risk factors, but based on limited evidence<br>Tieder JS, Bonkowsky JL, Etzel RA, Franklin WH, Gremse DA, Herman B, Katz ES, Krilov LR, Merritt JL, Norlin C, Percelay J. Brief resolved unexplained events (formerly apparent life-threatening events) and evaluation of lower-risk infants. Pediatrics. 2016 May 1;137(5).<br>NICE: no guidelines identified<br><br>Brand, D. A., & Fazzari, M. J. (2018). Risk of death in infants who have experienced a brief resolved unexplained event: a meta-analysis. The Journal of pediatrics, 197, 63-67.<br>Tieder, J. S., Altman, R. L., Bonkowsky, J. L., Brand, D. A., Claudius, I., Cunningham, D. J., ... & Smith, M. B. (2014). Management of apparent life-threatening events in infants: a systematic review. Pediatria Polska, 89(3), T15-T29.<br><br>Verdict: The evidence shows that risk of death is same as baseline risk. We did not find evidence on any other risk factors. **The question has NOT been addressed in the evidence base**	1 x friend/family member
72	What are effective interventions for hospitalized children and youth with sickle cell disease on the general paediatric inpatient unit?<br><br>E.g. Hyperhydration, oxygen, antibiotics, pain medication	1) Outcomes related to various common practices in the management of sick cell crises (e.g., hyperhydration, oxygen)	CPS: No guideline identified<br>AAP: No guideline identified<br>NICE: proposed treatment with voxelotor and crizanlizumab. Nothing published National Institute of Health and Care Excellence, Sickle cell acute painful episode Evidence Update May 2014; https://www.nice.org.uk/guidance/cg143/documents/cg143-sickle-cell-acute-painful-episode-evidence-update2<br><br>Alghamdi, M., & Al-Shahrani, M. (2020). 38. Ketamine for painful sickle cell vaso-occlusive crises: a systematic review and meta-analysis. European Journal of Emergency Medicine, 27(1), e14-e15. [results inconclusive for children]	1 x physician

© 2022 Gill PJ et al. *JAMA Network Open*.

			Dunlop, R., & Bennett, K. C. (2006). Pain management for sickle cell disease in children and adults. Cochrane Database of Systematic Reviews, (2). [results are inconclusive]<br>Mulaku, M., Opiyo, N., Karumbi, J., Kitonyi, G., Thoithi, G., & English, M. (2013). Evidence review of hydroxyurea for the prevention of sickle cell complications in low-income countries. Archives of disease in childhood, 98(11), 908-914.<br>Saramba, M. I., Shakya, S., & Zhao, D. (2020). Analgesic management of uncomplicated acute sickle-cell pain crisis in pediatrics: a systematic review and meta-analysis. Jornal de pediatria, 96(2), 142-158.<br>Yawn, B. P., Buchanan, G. R., Afenyi-Annan, A. N., Ballas, S. K., Hassell, K. L., James, A. H., ... & John-Sowah, J. (2014). Management of sickle cell disease: summary of the 2014 evidence-based report by expert panel members. Jama, 312(10), 1033-1048.<br><br>Verdict: Some evidence in favor of Hydroxyurea in management of pathogenesis of sickle cell. For analgesic, evidence is inconclusive. Hydroxyurea and transfusion therapy are strongly recommended for many individuals with SCD (moderate evidence in favor of children). The exact query was not addressed. Seems partial. **The question has been PARTIALLY addressed in the evidence base**	
73	How should children and youth with non-cancer related febrile neutropenia be managed on the general paediatric inpatient unit?	Treatment of children with non-cancer neutropenia and fever	CPS: No guideline identified<br>AAP: No guideline identified<br>NICE: No guideline identified<br><br>Sung, L. (2012). Initial Management of Low-Risk Pediatric Fever and Neutropenia: Efficacy and Safety, Costs, Quality-of-Life Considerations, and Preferences. American Society of Clinical Oncology Educational Book, 32(1), 570-574.<br>Jessica Morgan, Jemma Cleminson, Bob Phillips, Karl Atkin, Lesley Stewart. Systematic review of reductions in therapy for children with low risk febrile neutropenia. PROSPERO 2014 CRD42014005817 Available from: https://www.crd.york.ac.uk/prospero/display_record.php?ID=CRD42014005817 [In-progress]<br>Costa V, Ungar W. Caspofungin in the empiric treatment of febrile neutropenia in paediatric patients: A comparison with conventional and liposomal amphotericin B. Toronto: Technology Assessment at SickKids (TASK). Report No. 2008-01. 2008 [In-progress]<br>Sung L, Manji A, Beyene J, Dupuis LL, Alexander S, Phillips R, Lehrnbecher T. Fluoroquinolones in children with fever and neutropenia: a systematic review of prospective trials. Pediatric Infectious Disease Journal 2012; 31(5): 431-435 [In-progress]<br><br>Verdict: No evidence found but much work in progress. **The question has NOT been addressed in the evidence base**	1 x physician
74	What are evidence-based guidelines on the management of infants under 1 month / 3 month with fever without a source?	1. Febrile infants under 30 days old: can LOS be reduced from the traditional 48h if certain clinical and biologic criteria met? After how long are cultures (blood, CSF, urine) positive if pathogenic? (some research on this but little in Canada)<br>Q#2 Can there be a CPG for infants with fever without source (age <90 days)?	CPS: No guideline identified<br>AAP: No guideline identified<br>NICE: Guideline available on diagnosis and treatment but limited evidence referenced.<br>National Institute of Health and Care Excellence, Fever in under 5s: assessment and initial management; www.nice.org.uk/guidance/ng143<br><br>Purssell, E. (2011). Systematic review of studies comparing combined treatment with paracetamol and ibuprofen, with either drug alone. Archives of disease in childhood, 96(12), 1175-1179. [> 6 months]<br>Tan, E., Braithwaite, I., McKinlay, C. J., & Dalziel, S. R. (2020). Comparison of Acetaminophen (Paracetamol) With Ibuprofen for Treatment of Fever or Pain in Children Younger Than 2 Years: A Systematic Review and Meta-analysis. JAMA	2 x physician

© 2022 Gill PJ et al. *JAMA Network Open*.

			network open, 3(10), e2022398-e2022398. [<2 yrs]<br>Van den Bruel, A., Thompson, M. J., Haj-Hassan, T., Stevens, R., Moll, H., Lakhanpaul, M., & Mant, D. (2011). Diagnostic value of laboratory tests in identifying serious infections in febrile children: systematic review. Bmj, 342. [Ambulatory]<br>Wallace, S. S., Lopez, M. A., & Caviness, A. C. (2017). Impact of enterovirus testing on resource use in febrile young infants: a systematic review. Hospital pediatrics, 7(2), 96-102. [0 to 3 months]<br>Watts, R., & Robertson, J. (2012). Non-pharmacological management of fever in otherwise healthy children. JBI Evidence Synthesis, 10(28), 1634-1687. [3 months to 18 yrs]<br><br>Verdict: No evidence-based guidelines available. NICE guideline only suggests what symptoms to observe, what diagnostics to carry and to provide parenteral antibiotics based on certain criteria. **The question has NOT been addressed in the evidence base.**	
75	What are effective non-sedate (eg. feed and sleep) diagnostic tests (eg. CT scans) for children and youth admitted on a general paediatric inpatient unit?	What is the success rate of non-sedate (eg. feed and sleep) diagnostic tests (eg. CT scans) in children at different ages?<br>Are there non-invasive interventions which increase this success rate?	CPS: No guideline identified<br>AAP: No guideline identified<br>NICE: No guideline identified<br><br>Heye, P., Saavedra, J., Victoria, T., & Laje, P. (2020). Accuracy of unenhanced, non-sedated MRI in the diagnosis of acute appendicitis in children. Journal of pediatric surgery, 55(2), 253–256. https://doi.org/10.1016/j.jpedsurg.2019.10.039<br><br>Verdict: Only evidence found is focused on non-sedated MRI for a particular cohort of cases based on a review that was not a systematic review. **The question has NOT been addressed in the evidence base.**	1 x nurse

© 2022 Gill PJ et al. *JAMA Network Open*.

eMethods 1. Pediatric Hospital Care Priority Setting Partnership Protocol May 25, 2020 Version 2

1. Purpose of the PSP and background

The purpose of this protocol is to clearly set out the aims, objectives and commitments of the **Pediatric Hospital Care** Priority Setting Partnership (PSP) in line with James Lind Alliance (JLA) principles. The Protocol is a JLA requirement and will be published on the PSP's page of the JLA website. The Steering Group will review the Protocol regularly and any updated version will be sent to the JLA.

The JLA is a non-profit making initiative, established in 2004. It brings patients, carers and clinicians together in PSPs. These PSPs identify and prioritise the evidence uncertainties, or 'unanswered questions', that they agree are the most important for research in their topic area. Traditionally PSPs have focused on uncertainties about the effects of treatments, but some PSPs have chosen to broaden their scope beyond that. The aim of a PSP is to help ensure that those who fund health research are aware of what really matters to patients, carers and clinicians. The National Institute for Health Research (NIHR – www.nihr.ac.uk) coordinates the infrastructure of the JLA to oversee the processes for PSPs, based at the NIHR Evaluation, Trials and Studies Coordinating Centre (NETSCC), University of Southampton.

Pediatric Hospital Care Priority Setting Partnership

Optimal care of hospitalized children is essential to improve health outcomes. Admission to hospital can be a vulnerable period for children and families due to both the physical effects of serious illness and the psychologic effects of the illness and hospitalization. There are also negative economic implications including impacts on families due to work absences and the indirect costs of hospitalization (e.g. transportation). The largest population of children cared for in hospital are those with medical conditions managed in general pediatric inpatient units (GPIUs). In Canadian pediatric health centers, conditions cared for in GPIUs are considered to be the most common conditions leading to hospitalization. Children admitted to GPIUs may be previously healthy and be hospitalized for acute, common illness (e.g. gastroenteritis) or the first presentation of a chronic condition (e.g. epilepsy). They may also be children with a chronic disease (e.g. asthma) or medical complexity (e.g. cerebral palsy and gastrostomy feeding) who are hospitalized for an acute, common illness or exacerbation of their chronic disease.

Children admitted to GPIUs may not receive optimal health care due to a lack of high-quality research. We recently established the Canadian Pediatric Inpatient Research Network (PIRN), which is a hospital-based research network focused on generating scientific knowledge to improve the outcomes of hospitalized children in GPIUs through the conduct of patient-oriented research. PIRN includes hospital sites affiliated with all 15 academic children's hospitals across Canada as well as several large community hospitals. To guide future research priorities in pediatric hospital care and for PIRN, we aim to identify GPIU clinical management questions that are important to patients (i.e. children and youth), carers (i.e. parents, family and caregivers), and healthcare professionals (HCPs).

2. Aims, objectives and scope of the PSP

The aim of the **Pediatric Hospital Care** PSP is to identify the unanswered questions about **pediatric hospital care in general pediatric inpatient units (GPIUs)** from patient, carer and clinical perspectives and then prioritise those that patients, carers and clinicians agree are the most important for research to address.

The objectives of the PSP are to:

- work with patients, carers and clinicians to identify uncertainties about pediatric medical conditions managed in GPIUs
- agree by consensus a prioritised list of those uncertainties, for research
- publicise the results of the PSP and process
- take the results to research commissioning bodies to be considered for funding.

Scope of the PSP

The Pediatric Hospital Care PSP will cover clinical management questions relevant to the care of children (age between 0 and 18 years) hospitalized in a GPIU. Clinical management broadly refers to the diagnosis and treatment of conditions, which includes processes of care (e.g. how to promote better sleep in hospitalized children) and specific conditions (e.g. blood tests to diagnose infection, interventions for asthma care).

GPIUs may be located either on a hospital ward in a children's hospital or a general community hospital. While there is variation between Canadian hospitals regarding the types of conditions cared for in GPIUs, the PSP will focus on and include children with conditions where the management is led by general pediatricians. For example, children that are cared for in a GPIUs may be previously healthy and be hospitalized for acute, common illness (e.g. gastroenteritis) or the first presentation of a chronic condition (e.g. epilepsy). They may also be children with a chronic disease (e.g. asthma) or medical complexity (e.g. cerebral palsy and gastrostomy feeding) who are hospitalized for an acute, common illness or exacerbation of their chronic disease. The scope will include children with technology-dependent conditions.

Given the breadth of childhood mental health problems, we will exclude questions related to the management of known mental health problems in-hospital (e.g. depression) which would require specialist input (e.g. psychiatry). For example, questions related to diagnostic tests (e.g. blood tests, head imaging) of children who present with new onset hallucinations would be included, as these patients are generally cared for in a GPIU, but questions focused on the types of treatments used for hospitalized children with schizophrenia would be excluded. We will exclude questions related to chronic or pre-existing mental health problems of parents and caregivers.

As the scope is focused on care in a GPIU, we will exclude children who are seen in specialized settings of care, including the pediatric intensive care unit (PICU), the neonatal intensive care unit (NICU), medical subspecialty units (e.g. oncology), psychiatric, and surgical inpatient units. Similarly, we will exclude questions related to etiology or prognosis, and uncertainties related to healthcare organization and/or healthcare delivery (e.g. structure of inpatient unit, or staffing levels). We will also exclude uncertainties related to children who receive treatments outside of Canada.

Given the range of conditions, the scope will be further refined as needed by the Steering Group as the PSP progresses. The Steering Group is responsible for discussing what implications the scope of the PSP will have for the evidence-checking stage of the process. Resources and expertise will be put in place to do this evidence checking.

3. The Steering Group
The Steering Group includes membership of patients and carers and clinicians, as individuals or representatives from a relevant group.

The Pediatric Hospital Care PSP will be led and managed by a Steering Group involving the following:

Patient representative/s:
- Francine Buchanan, Co-Chair of the SickKids Research Family Advisory Counsel
- Michelle Quinlan, Co-Chair Children's Hospital of Eastern Ontario (CHEO) – Research Institute Family Leader Program Coordinator
- Shelley Frappier, Parent of a child who was hospitalized in a GPIU
- Kim De Castris-Garcia, Parent of a child who was hospitalized in a GPIU
- Mairead Green, Young person who was hospitalized in a GPIU

Clinical representatives:
General Pediatric representatives
- Sanjay Mahant, Academic General Pediatrician, Hospital for Sick Children
- Ann Bayliss, Community General Pediatrician, Trillium Health Partners

Nursing representatives
- Karen Breen-Reid, Registered Nurse and Manager Interprofessional Education, Hospital for Sick Children
- Aubrey Sozer, Registered Nurse and Interim Interprofessional Education Specialist for Paediatric Medicine, Hospital for Sick Children

Co-Chair of the Steering Group
- Peter Gill, Academic General Pediatrician, Hospital for Sick Children

James Lind Alliance Adviser and Co-Chair of the Steering Group:

- Katherine Cowan, Senior Advisor to the JLA

PSP Project Coordinator

- Dana Arafeh, Hospital for Sick Children

The Steering Group will agree the resources, including time and expertise that they will be able to contribute to each stage of the process, with input and advice from the JLA.

4. Partners

Organisations and individuals will be invited to be involved with the PSP as partners. Partners are organisations or groups who will commit to supporting the PSP, promoting the process and encouraging their represented groups or members to participate. Organisations which can reach and advocate for these groups will be invited to become involved in the PSP. Partners represent the following groups:

- people who have had conditions that required hospitalization in the general pediatric inpatient unit
- carers of people who have been hospitalized in a general pediatric inpatient unit
- medical doctors, nurses and allied health care professionals with clinical experience caring for hospitalized children in a general pediatric inpatient unit

It is important that all organisations, which can reach and advocate for these groups should be invited to become involved in the PSP. The JLA Adviser will take responsibility for ensuring the various stakeholder groups are able to contribute equally to the process.

Exclusion criteria

Some organisations may be judged by the JLA or the Steering Group to have conflicts of interest. These may be perceived to potentially cause unacceptable bias as a member of the Steering Group. As this is likely to affect the ultimate findings of the PSP, those organisations will not be invited to participate. It is possible, however, that interested parties may participate in a purely observational capacity when the Steering Group considers it may be helpful.

5. The methods the PSP will use

This section describes a schedule of proposed steps through which the PSP aims to meet its objectives. The process is iterative and dependent on the active participation and contribution of different groups. The methods used in any step will be agreed through consultation between the Steering Group members, guided by the PSP's aims and objectives. More details of the method are in the Guidebook section of the JLA website at www.jla.nihr.ac.uk where examples of the work of other JLA PSPs can be seen.

Step 1: Identification and invitation of potential partners

Potential partner organisations will be identified through a process of peer knowledge and consultation, through the Steering Group members' networks. Potential partners will be contacted and informed of the establishment and aims of the **Pediatric Hospital Care** PSP.

Step 2: Awareness raising

PSPs will need to raise awareness of their proposed activity among their patient, carer and clinician communities, in order to secure support and participation. Depending on budget, this may be done by a face-to-face meeting, or there may be other ways in which the process can be launched, e.g. via social media. It may be carried out as part of steps 1 and/or 3. The Steering Group should advise on when to do this. Awareness raising has several key objectives:

- to present the proposed plan for the PSP
- to generate support for the process
- to encourage participation in the process
- to initiate discussion, answer questions and address concerns.

Step 3: Identifying evidence uncertainties

The **Pediatric Hospital Care** PSP will carry out a consultation to gather uncertainties about clinical management from patients, carers and clinicians. The Steering Group will develop an online questionnaire in Research Electronic Data Capture (REDCap), a secure web-based application, to identify unanswered questions about pediatric hospital care in GPIUs. The questionnaire will include a section on demographic, an open-ended statement asking participants to identify questions about the care in GPIUs, and may include questions focused on specific topics previously identified as important (e.g. HCP-patient/carer communication). The questionnaire will be pilot tested and refined, and will be approved by the Steering Group before it is broadly disseminated through strategic partners. The survey will remain open for 3 months and will be monitored for diversity. We will also have limited printed copies of the questionnaire available with free return postage.

We aim to obtain a diverse range of responses based on geography, age, sex/gender, health state of child/youth (i.e. healthy, chronic illness, medical complexity), ethnicity and language (survey will be translated into French). We will also target vulnerable groups, including indigenous, non-native English speakers and high social risk groups. In order to ensure broad reach of all communities including marginalised populations, multiple recruitment approaches will be used to ensure diversity in those who are participating. The **Pediatric Hospital Care** PSP will be advertised through a website, social media, posters as well as flyers which will be circulated through well-known distribution lists and through partner organisations.

Existing sources of information about clinical management uncertainties will also be searched by examining published systematic reviews, clinical guidelines, protocols and on-going study registers.

Step 4: Refining questions and uncertainties
The consultation process will produce 'raw' questions and comments indicating patients', carers' and clinicians' areas of uncertainty. These raw questions will be categorised and refined by the PSP Project Coordinator into summary questions which are clear, addressable by research, and understandable to all. Similar or duplicate questions will be combined where appropriate. Out-of-scope and 'answered' submissions will be compiled separately. The Steering Group will have oversight of this process to ensure that the raw data is being interpreted appropriately and that the summary questions are being worded in a way that is understandable to all audiences. The JLA Adviser will observe to ensure accountability and transparency. The Steering Group will target 40-50 summary questions.

The summary questions will then be checked against evidence to determine whether they have already been answered by research. This will be done by an Information Specialist, who will complete the JLA Question Verification Form, which clearly describes the process used to verify the uncertainty of the questions, before starting prioritisation. The Question Verification Form includes details of the types and sources of evidence used to check uncertainty. We will work with an experienced librarian to search MEDLINE, EMBASE, CINAHL and the Cochrane Central Register of Controlled Trials to look for relevant studies. The Question Verification Form should be published on the JLA website as soon as it has been agreed to enable researchers and other stakeholders to understand how the PSP has decided that its questions are unanswered, and any limitations of this.

Questions will be considered unanswered (i.e. '*true uncertainties')* if there is no systematic review, if a recent systematic review indicated insufficient evidence, or if there is insufficient evidence outlined in position statements from the Canadian Paediatric Society and American Academy of Pediatrics. Questions which can be resolved by reference to existing literature will classified as '*answerable questions*' and dealt with separately. The Steering Group will consider how it will deal with submitted questions that have been answered, and questions that are out of scope.

Step 5: Prioritisation – interim and final stages
The aim of the final stage of the PSP is to prioritise through consensus the identified uncertainties about conditions cared for in GPIUs. The final step will involve input from patients, carers and clinicians. The JLA encourages PSPs to involve as wide a range of people as possible, including those who did and did not contribute to the first consultation. The format of the final prioritization process will be agreed by members of the Steering Group, which usually consists of two stages:

1. Interim prioritisation is the stage where the long list of questions is reduced to a shorter list that can be taken to the final priority setting workshop. This is aimed at a wide audience, and is done using similar methods to the first

consultation. With the JLA's guidance, the Steering Group will agree the method and consider how best to reach and engage patients, carers and clinicians in the process. The most highly ranked questions (around 25) will be taken to a final priority setting workshop. Where the interim prioritisation does not produce a clear ranking or cut off point, the Steering Group will decide which questions are taken forwards to the final prioritisation.

2. The final priority setting stage is generally a one-day workshop facilitated by the JLA. With guidance from the JLA and input from the Steering Group, up to 30 patients, carers and clinicians will be recruited to participate in a day of discussion and ranking, to determine the top 10 questions for research. All participants will declare their interests. The Steering Group will advise on any adaptations needed to ensure that the process is inclusive and accessible. The workshop will be led by JLA Advisor Katherine Cowan and use a modified nominal group technique, a well-established structured, multistep facilitated group meeting technique. Two additional JLA facilitators will manage the discussions, to ensure balanced contributions from carers and healthcare professionals.

6. Dissemination of results
Findings and research
The findings of the Pediatric Hospital Care PSP will be reported to funding and research agenda setting organisations such as the Canadian Institutes for Health Research, the Canadian Pediatric Society, Children's Healthcare Canada, and the Maternal Infant Child and Youth Research Network. We will disseminate the unanswered questions through our knowledge users and partners, and by posting the results on the JLA website. The Steering Group will determine how best to communicate the results and who will take responsibility for this. Steering Group members and partners are encouraged to develop the prioritised uncertainties into research questions, and to work to establish the research needs of those unanswered questions to use when approaching potential funders, or when allocating funding for research themselves, if applicable.

Publicity
As well as alerting funders, partners and Steering Group members are encouraged to publish the findings of the Pediatric Hospital Care PSP using both internal and external communication mechanisms. The Steering Group may capture and publicise the results through descriptive reports of the process itself in plain language. This exercise will be distinct from the production of an open-access academic paper, which will also be completed. However, production of an academic paper should not take precedence over publicising the final results. Additional potential outputs include infographics, conference presentations and videos for social media.

The JLA encourages PSPs to report back about any activities that have come about because of the PSP, including funded research. Please send any details to jla@soton.ac.uk.

7. Agreement of the Steering Group
The Pediatric Hospital Care PSP Steering Group agreed the content and direction of this Protocol on 28 May 2020.

Protocol posted online at: https://www.jla.nihr.ac.uk/documents/pediatric-hospital-care-canada-protocol/25037

eMethods 3. Phase 1 initial survey used in Pediatric Hospital Care Priority Setting Partnership

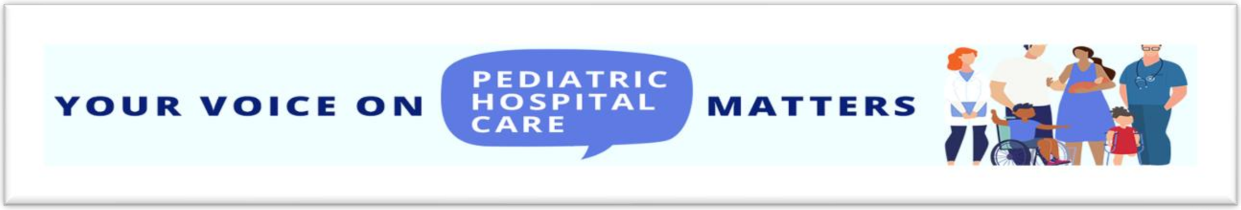

Pediatric Hospital Care Priority Setting Partnership Survey (English)

This survey will take 10-15 minutes to complete.

We are a team of parents, caregivers, youth, healthcare providers, and researchers who are working together to identify research priorities related to pediatric hospital care.

If you are (or used to be):

- A child or young person (0-18) who was hospitalized on a general pediatric ward
- A mother, father or caregiver to a child who was hospitalized on a general pediatric ward
- A family member of a child or teen who was hospitalized on ageneral pediatric ward
- A healthcare provider who takes care of children on a general pediatric ward

We want to hear from you!

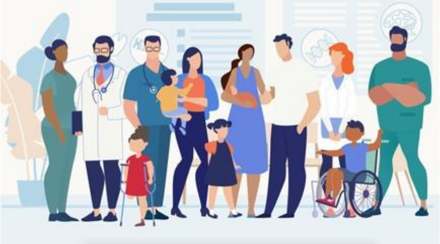

Think about your own personal or professional experience. What concerns, comments, or questions about the management of children or teenagers and their families would you like answered by research? Your questions will help us develop solutions that would improve the care of children and families in the future.

If you are unsure about what is or is not included in our study, please visit our website at www.pirncanada.com

Which of the following best describes you?

Please select only one.

◯ Child or young person who was admitted to a general pediatric ward
◯ Parent (Mother or Father) or caregiver of a child that was admitted to a general pediatric ward Friend/family
◯ member of someone admitted to a general pediatric ward
◯ Nurse Physician
◯ Dietician
◯ Child life specialist
◯ Respiratory therapist
◯ Pharmacist
◯ Social Worker
◯ Physiotherapist Occupational
◯ therapist
◯ Other
◯

If other, please specify

____

Section 1: Your Questions

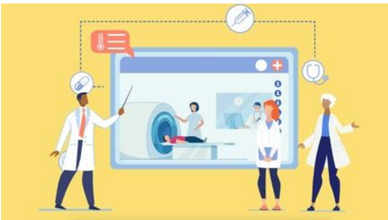

Are you a patient, parent or caregiver? Think about your experience during any admission to hospital under general pediatrics. Think about questions that you had during the hospital stay or after you left the hospital that were left unanswered, were confusing, or that you may have been curious about.

Are you a healthcare provider? Are there any uncertainties about patient care during the hospital stay in a general pediatric ward, or areas where you feel there should be more evidence to guide care?

Please think broadly about your questions, which may include questions about:

- **Treatments**, including medications (e.g. injections, pills, syrups, puffers, or ointments), diet, feeding, occupational/physiotherapy, etc.
- **Tests**, including blood tests, urine tests, or imaging such as x-rays, ultrasound, CT, MRI scans, etc.
- **Other aspects of care**, including communications, interactions with healthcare professionals, use of technology, patient safety, monitoring in hospital, etc.

Please share these questions with us! There are no right or wrong answers, and do not feel like you need to check if this question has already been answered by research.

Q#1: What concerns, comments or questions do you have about the care of children in hospital on the general pediatric ward that you would like answered by research?

Q#2: What other concerns, comments or questions do you have about the care of children in hospital on the general pediatric ward that you would like answered by research?

Q#3: What other concerns, comments or questions do you have about the care of children in hospital on the general pediatric ward that you would like answered by research?

Section 2: Background

1. What is your current age in years?

____

2. How do you identify

- ◯ Male
- ◯ Female
- ◯ Other
- ◯ Prefer not to say

If other, please Specify

____

3. What is your ethnic/cultural background? (Check all that apply)

- ☐ White - North American or European)
- ☐ First Nation/Indigenous/Aboriginal (Métis,First Nations, Inuit)
- ☐ European
- ☐ Caribbean
- ☐ Latin, Central or South American African
- ☐ Oceania (e.g. Australian)
- ☐ Asian (e.g. Middle Eastern, South, East, and Southeast
- ☐ Asian)
- ☐ Prefer not to say
- ☐ Other

If other, please specify

____

4. Language spoken at home:

- ☐ English
- ☐ French
- ☐ Other

If other, please specify

____

5. What province or territory do you live in?

- ◯ Alberta
- ◯ British Columbia
- ◯ Manitoba
- ◯ New Brunswick
- ◯ Nova Scotia
- ◯ Northwest Territories
- ◯ Newfoundland and Labrador
- ◯ Nunavut
- ◯ Ontario
- ◯ Prince Edward Island
- ◯ Quebec
- ◯ Saskatchewan
- ◯ Yukon

6. What best describes where you live?

- ◯ Urban
- ◯ Rural

7. How many times have you or your child been hospitalized?

- ◯ 1-2
- ◯ 3-5
- ◯ greater than 5 Not
- ◯ applicable

8. What was the age of you or your child when they admitted to hospital?	◯ Under 3 months<br>◯ 3 months to 2 years<br>◯ 2 to 5 years<br>◯ 5 to 9 years<br>◯ 10 to 14 years<br>◯ 15 to 18 years<br>◯ Prefer not to say<br>◯ Not applicable
9. In a few words, please describe the reason that you or your child was most recently admitted to hospital.	____________________________<br>(Please enter NA if not applicable)
10. For health and/or social care practitioners, what setting do you practice in (select all that apply)?	◯ Community hospital in urban/sub-urban area (e.g. greater Toronto area)<br>◯ Community hospital in rural area<br>◯ Academic children's hospital<br>◯ Prefer not to say<br>◯ Other<br>◯ Not applicable
If other, please specify	____________________________

Thank you for completing this questionnaire!

The next phase of the study involves putting the research topics identified from this questionnaire into order of importance. If you would like to go through the list of research topics and tell us which ones you think are most important, or if you would like to stay informed on project updates, please visit our website and provide your contact information or contact the study office by telephone, email or in writing to:

Email: pediatrichospitalcare.psp@sickkids.ca
Peter Gilgan Centre for Research and Learning
686 Bay Street, Toronto, ON M5G 0A4
Phone: 416-813-7654 (ext 228525)

For more information on PIRN, our project and timelines please visit us: www.pirncanada.com

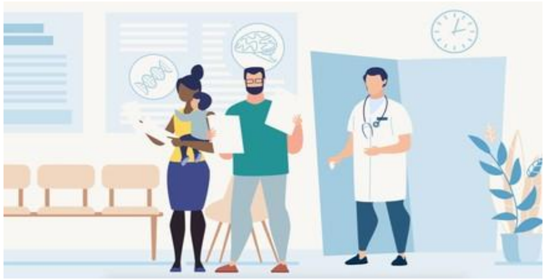

eMethods 2. Pediatric Hospital Care Priority Setting Partnership Question Verification Form

The purpose of this Question Verification Form is to enable Priority Setting Partnerships (PSPs) to describe clearly how they checked that their questions were unanswered, before starting the interim prioritisation stage of the process.

The JLA requires PSPs to be transparent and accountable in defining their own scope and evidence checking process. This will enable researchers and other stakeholders to understand how individual PSPs decided that their questions were unanswered, and any limitations of their evidence checking. For an example of this form completed by a PSP, please see http://www.jla.nihr.ac.uk/priority-setting-partnerships/hyperacusis/downloads/Hyperacusis-PSP-question-verification-form.pdf

**Name of the PSP**
Paediatric Hospital Care (Canada)
**Please describe the scope of the PSP**
The scope of the Pediatric Hospital Care PSP focuses on questions related to the clinical management of children (age between 0 and 18 years) hospitalized in a general pediatric inpatient unit (GPIU). Clinical management broadly refers to the diagnosis and treatment of conditions, which includes processes of care and specific conditions. While there is variation between Canadian hospitals regarding the types of conditions cared for in GPIUs, the PSP will focus on and include children with conditions where the management is led by general pediatricians. For example, children that are cared for in a GPIUs may be previously healthy and be hospitalized for acute, common illness (e.g. gastroenteritis) or the first presentation of a chronic condition (e.g. epilepsy). They may also be children with a chronic disease (e.g. asthma) or medical complexity (e.g. cerebral palsy and gastrostomy feeding) who are hospitalized for an acute, common illness or exacerbation of their chronic disease. The scope will include children with technology-dependent conditions.<br><br>The following topics are out of scope:<br>• Questions related to the management of known mental health problems in-hospital (e.g. depression) which would require specialist input (e.g. psychiatry)<br>• Questions related to chronic or pre-existing mental health problems of parents and caregivers.<br>• Questions related to the care of children seen in specialized settings of care, including the pediatric intensive care unit (PICU), the neonatal intensive care unit (NICU), medical subspecialty units (e.g. oncology), psychiatric, and surgical inpatient units.<br>• Questions related to etiology or prognosis<br>• Questions related to healthcare organization and/or healthcare delivery.<br>• Questions related to children who receive treatments outside of Canada.
**Please provide a brief overview of your approach to checking whether the questions were unanswered**
For reasons of practicality, highly focused and targeted searches were conducted to identify systematic reviews rather than a full systematic approach (which would have been impractical within the timeframe of the PSP). We also conducted focused keyword / free text only search that excluded the use of thesaurus terms.<br><br>Given the broad scope of our PSP, questions were considered unanswered if there was no systematic review, if a recent systematic review indicated insufficient evidence, or if there is insufficient evidence outlined in position statements from the Canadian Paediatric Society and American Academy of Pediatrics.
**Please list the type(s) of evidence you used to verify your questions as unanswered**
The evidence used to verify the questions are unanswered were as follows:<br>• Systematic reviews<br>• Clinical guidelines
**Please list the sources that you searched in order to identify that evidence**
The OVID interface at SickKids was used to search the following health and medical bibliographic databases:<br>• MEDLINE

• EMBASE<br>• The Cochrane Library<br><br>Other resources included in the search were as follows:<br>• Cumulative Index to Nursing and Allied Health Literature (CINAHL)<br>• American Academy of Pediatrics (AAP) and Canadian Paediatric Society (CPS) Guidelines<br>• NICE/SIGN guidelines<br>• TRIP Database<br>• Google Scholar<br>• Epistemonikos
**What search terms did you use?**
Groups of the following sets were combined depending on the type of question:<br><br>**Set 1:** Used always<br><br>(systematic review* or meta analys* or meta-analys* or metaanaly* or meta synthes* or meta-synthes* or scoping review* or rapid review*).ti,ab.<br><br>**Set 2:** Used always<br><br>(pediatric* or paediatric* or child* or newborn* or infan* or baby or babies or neonat* or premature birth* or juvenile* or teen* or youth* or adolesc*).ti. or (pediatric* or paediatric* or child* or newborn* or infan* or baby or babies or neonat* or premature birth* or juvenile* or teen* or youth* or adolesc*).ab. /freq=2 or (child* or adolesc* or pediat* or paediat*).jn.<br><br>**Set 3:** Used always<br><br>(hospital* or inpatient* or NICU or PICU or intensive care unit or patient admission* or patient discharge* or medical admission unit or observation unit* or short stay unit* or length of stay or hospital stay* or emergency department or emergency ward or emergency room or emergency unit or "Accident and Emergency" or emergency unit* or healthcare setting? or health care setting? or healthcare facilit* or health care facilit*).ti,ab.<br><br>**Set 4:** Used always - words to reflect the concepts or focus in the questions – searched in document titles and abstracts.
**Please describe the parameters of the search (eg time limits, excluded sources, country/language) and the rationale for any limitations**
Eligibility criteria used for the inclusion of evidence was as follows:<br>• To ensure retrieval of the most recent literature, searches were restricted to the last 10 years (2010 onwards).<br>• Search limited to English only articles
**Names of individuals who undertook the evidence checking**
Dr. Rashid Anwar
**On what date was the question verification process completed?**
December 2020 to April 2021
**Any other relevant information**
N/A

Version 10. Date 28 September 2021
