# Supplementary material for: Patient, Caregiver, and Clinician Participation in Prioritization of Research Questions in Pediatric Hospital Medicine
Source: JAMA Netw Open. 2022 Apr 26;5(4):e229085. doi: 10.1001/jamanetworkopen.2022.9085 (PMC9044112; doi:10.1001/jamanetworkopen.2022.9085)
Supplement: Supplement 2. — Nonauthor Collaborators. The Canadian Pediatric Inpatient Research Network (PIRN) [file jamanetwopen-e229085-s002.pdf]

Supplemental Online Content: Nonauthor Collaborators
*First name, last name, and suffix (if applicable) are required and will appear in PubMed.

| *Group Name(s): Canadian Pediatric Inpatient Research Network (PIRN) | | | | | | | |
|---|---|---|---|---|---|---|---|
| ***First Name and Middle Initial(s)** | ***Last Name** | ***Suffix (eg, Jr, III)** | **Academic Degrees** | **Institution** | **Location (city, state/province, country)** | **Role or Contribution, eg, chair, principal investigator** | **Group (if more than 1 Group listed in the byline) and/or Subgroup (eg, Steering Committee)** |
| Olivier | Drouin | | MD, MSc, MPH, FRCPC | Centre Hospitalier Universitaire Ste-Justine | Montreal, PQ, Canada | Co-investigator | Canadian Pediatric Inpatient Research Network (PIRN) |
| Catherine | Pound | | MD, FRCPC | Children's Hospital of Eastern Ontario | Ottawa, ON, Canada | Co-investigator | Canadian Pediatric Inpatient Research Network (PIRN) |
| Gita | Wahi | | MD, MSc, PhD, FRCPC | McMaster | Hamilton, ON, Canada | Co-investigator | Canadian Pediatric Inpatient Research Network (PIRN) |
| Gemma | Vomiero | | MD, FRCPC | Alberta Children's Hospital | Calgary, AB, Canada | Co-investigator | Canadian Pediatric Inpatient Research Network (PIRN) |
| Kim | Zhou | | MD, FRCPC | North York General Hospital | Toronto, ON, Canada | Co-investigator | Canadian Pediatric Inpatient Research Network (PIRN) |
| Mahmoud | Sakran | | MBBS, MD | Lakeridge Health Research Institute | Oshawa, ON, Canada | Co-investigator | Canadian Pediatric Inpatient Research Network (PIRN) |
| Anupam | Sehgal | | MB, DNB, FCCM | Queen's University | Kingston, ON, Canada | Co-investigator | Canadian Pediatric Inpatient Research Network (PIRN) |
| Thuy Mai | Luuy | | MD, MSc, FRCPC | Centre Hospitalier Universitaire Ste-Justine | Montreal, PQ, Canada | Co-investigator | Canadian Pediatric Inpatient Research Network (PIRN) |
| Karen | Forbes | | MD, FRCPC | PICU Stollery Children's Hospital | Edmonton, AB, Canada | Co-investigator | Canadian Pediatric Inpatient Research Network (PIRN) |

Supplemental Online Content: Nonauthor Collaborators
*First name, last name, and suffix (if applicable) are required and will appear in PubMed.

| *First Name and Middle Initial(s) | *Last Name | *Suffix (eg, Jr, III) | Academic Degrees | Institution | Location (city, state/province, country) | Role or Contribution, eg, chair, principal investigator | Group (if more than 1 Group listed in the byline) and/or Subgroup (eg, Steering Committee) |
|---|---|---|---|---|---|---|---|
| Tom | McLaughlin | | MD, MPH, FRCPC | BC Children's Hospital | Vancouver, BC, Canada | Co-investigator | Canadian Pediatric Inpatient Research Network (PIRN) |
| Krista | Baerg | | MD, FRCPC | Jim Pattison Children's Hospital | Saskatoon, SK, Canada | Co-investigator | Canadian Pediatric Inpatient Research Network (PIRN) |
| Geert | t Jong | | MD, PhD | Children's Hospital Research Institute of Manitoba | Winnipeg, MB, Canada | Co-investigator | Canadian Pediatric Inpatient Research Network (PIRN) |
| Sepi | Taheri | | MD, FRCPC | University of Western Ontario | London, ON, Canada | Co-investigator | Canadian Pediatric Inpatient Research Network (PIRN) |
| Patricia | Li | | MD, FRCPC | Research Institute, McGill University | Montreal, PQ, Canada | Co-investigator | Canadian Pediatric Inpatient Research Network (PIRN) |
| Evelyn | Constantin | | MD,MSc, FRCPC | Montreal Children's Hospital | Montreal, PQ, Canada | Co-investigator | Canadian Pediatric Inpatient Research Network (PIRN) |
| Joanna | Holland | | MD, FRCPC | IWK Health Centre | Halifax, NS, Canada | Co-investigator | Canadian Pediatric Inpatient Research Network (PIRN) |
| Leigh-Ann | Newhook | | MD, FRCPC | Janeway Pediatric Research Unit, Memorial Univeristy | St. John's, Nfld, Canada | Co-investigator | Canadian Pediatric Inpatient Research Network (PIRN) |
| Sean | Murray | | MD, FRCPC | Northern Ontario School of Medicine | Sudbury, ON, Canada | Co-investigator | Canadian Pediatric Inpatient Research Network (PIRN) |
